# *De novo* characterization of the gene-rich transcriptomes of two color-polymorphic spiders, *Theridion grallator* and *T. californicum* (Araneae: Theridiidae), with special reference to pigment genes

**DOI:** 10.1186/1471-2164-14-862

**Published:** 2013-12-08

**Authors:** Peter JP Croucher, Michael S Brewer, Christopher J Winchell, Geoff S Oxford, Rosemary G Gillespie

**Affiliations:** 1 Department of Environmental Science, Policy and Management, University of California, Berkeley, CA 94720-3114, USA; 2 Department of Molecular and Cell Biology, University of California, Berkeley, CA 94720-3200, USA; 3 Department of Biology (E019), University of York, Wentworth Way, Heslington, York YO10 5DD, UK

**Keywords:** Araneae, Spider, Color, Pigmentation, Polymorphism, Pteridine, Ommochrome, RNA-seq, cDNA

## Abstract

**Background:**

A number of spider species within the family Theridiidae exhibit a dramatic abdominal (opisthosomal) color polymorphism. The polymorphism is inherited in a broadly Mendelian fashion and in some species consists of dozens of discrete morphs that are convergent across taxa and populations. Few genomic resources exist for spiders. Here, as a first necessary step towards identifying the genetic basis for this trait we present the near complete transcriptomes of two species: the Hawaiian happy-face spider *Theridion grallator* and *Theridion californicum*. We mined the gene complement for pigment-pathway genes and examined differential expression (DE) between morphs that are unpatterned (plain yellow) and patterned (yellow with superimposed patches of red, white or very dark brown).

**Results:**

By deep sequencing both RNA-seq and normalized cDNA libraries from pooled specimens of each species we were able to assemble a comprehensive gene set for both species that we estimate to be 98-99% complete. It is likely that these species express more than 20,000 protein-coding genes, perhaps 4.5% (ca. 870) of which might be unique to spiders. Mining for pigment-associated *Drosophila melanogaster* genes indicated the presence of all ommochrome pathway genes and most pteridine pathway genes and DE analyses further indicate a possible role for the pteridine pathway in theridiid color patterning.

**Conclusions:**

Based upon our estimates, *T. grallator* and *T. californicum* express a large inventory of protein-coding genes. Our comprehensive assembly illustrates the continuing value of sequencing normalized cDNA libraries *in addition to* RNA-seq in order to generate a reference transcriptome for non-model species. The identification of pteridine-related genes and their possible involvement in color patterning is a novel finding in spiders and one that suggests a biochemical link between guanine deposits and the pigments exhibited by these species.

## Background

Visible polymorphisms provide tractable systems within which to examine the molecular basis of adaptation because of their often-simple patterns of inheritance and the general ease with which morph/allele frequencies can be estimated [1,2]. Many spider species show visible variation in color and pattern [3]. A number of unrelated species within the Theridiidae (cobweb spiders) exhibit a heritable color polymorphism. In most cases examined, the polymorphism consists of two or three morphs, as in the sister species *Enoplognatha ovata* Clerck and *Enoplognatha latimana* Hippa & Oksala [4,5]. Two other, distantly related species within the genus *Theridion* have become of particular interest because they exhibit a spectacular array of color morphs. The Hawaiian happy-face spider *Theridion grallator* Simon occurs in native forest on four of the Hawaiian islands and displays more than 20 discrete abdominal color patterns [6,7] while *Theridion californicum* is found along the Pacific coast of North-America and exhibits at least 12 discrete abdominal color patterns [8,9]. The morphs displayed by these species are remarkably similar, and in the case of *T. grallator* may have evolved repeatedly, subsequent to colonization of each of the Hawaiian islands [10]. Many of the morphs exhibited by these species are illustrated in references [9] and [9,11]. The most common morphs of each species are also convergent with those displayed by *Enoplognatha ovata*, *E. latimana* and some other polymorphic species in the Theridiidae. In all species examined there is a common Yellow morph that typically represents 60-70% of any population and that is recessive to all other morphs, with the Colored (patterned) morphs displaying a dominance hierarchy that broadly reflects the extent of pigmentation [3,4,8,9]. The morphs are created from a palette of yellow, red and dark-brown (almost black) pigments laid down on a reflective background of white guanine crystals and all the pigment appears to be ommochrome based [3,12].

These recurring patterns led Oxford [9] to propose that in the Theridiidae there is a common ground plan for patterning and that the occurrence of shared morphs across species implies canalization of the processes by which the color patterns are generated. Furthermore, studies in *E. ovata*, *T. grallator*, and *T. californicum* have shown that the inheritance of the color polymorphism follows a broadly Mendelian pattern with segregation at a single locus with multiple alleles [4,6,9]. The pattern of simple Mendelian inheritance is occasionally complicated by the presence of sex-limitation (see [3] for a review) in the expression of color morphs. Indeed, *T. grallator* individuals from the island of Hawaii, compared with those from Maui, have experienced a shift in the mode of inheritance of the polymorphism with possibly two loci involved and some color morphs exhibiting sex limitation [7,13]. This change in the pattern of inheritance has led to the suggestion that the color polymorphism has to some extent evolved independently on different islands [10,13].

### Chemical basis for spider coloration

Much of the pigment-based coloration in invertebrates results from products of the ommochrome, pteridine, papiliochrome, melanin and heme synthesis pathways [14]. To date only ommochrome and bilin-based pigments have been identified in spiders. Ommochromes, which are derivatives of the amino acid tryptophan, via kynurenine and 3-hydroxykynurenine, are the best-known spider pigments and are responsible for a wide range of colors from yellow through red to gold and very dark brown. The usual reduced form is red/brown and the oxidized form usually yellow [15]. Ommochromes have been the focus of considerable research in particular in the 1970s and 1980s [12,16-18]. More recent work on these pigments in spiders has largely been limited to color change in *Misumena vatia*, where it appears that color change is associated with a cyclic pattern of formation and degradation of pigment granules [19].

Bilins, which tend to be blue or green, have been found in the form of micromatabilin in the green huntsman spider *Micrommata virescens* (Sparassidae) [20,21]. In addition to these pigments, the purine-base guanine, a terminal excretory product in spiders, is often laid down in crystalline form in specialized guanocytes on the surface of the gut diverticula, directly beneath the hypodermis [3]). The guanine crystals produce a white or silvery coloration by reflection and scattering and are therefore structural colorants. However they are frequently directly associated with pigment-based colors, either contributing to the overall color pattern or acting as a reflective layer beneath pigmented areas [6,22-24].

Melanin is found, with a variety of structures, ubiquitously throughout the tree of life [14] and commonly plays a role in defense and isolation of infections. Melanin has however not been reported as a pigment in spiders [3]. Although identified in some mites [25,26], carotenoid pigments have also not been detected in spiders [3]. Perhaps most surprising is the apparent absence of pteridine-based pigments. The pteridine pathway is found in both plants and animals and a key compound in the pathway, tetrahydrobiopterin, acts as an essential cofactor in the degradation of phenylalanine and the synthesis of the neurotransmitters serotonin, melatonin, dopamine, norepinephrine and epinephrine [27]. Pteridine and ommochrome pigments form the basis of the visible eye-color variants of *Drosophila* and much of the variation in butterfly wing patterns, and have consequently been central to the development of genetics itself [28]. Indeed the plethora of observed eye-color mutants in *Drosophila* results from the complex spectral interactions of pteridine and ommochrome pigments. Given the use of guanine as a colorant in spiders, it is also interesting to note that this is the key substrate for the pteridine pathway (as opposed to tryptophan for the ommochrome pathway). Finally, many pigment proteins contain heme groups or result from conjugates of heme-containing compounds (e.g. bilins) [3,14].

The parallel evolution of genetically based adaptive changes amongst both unrelated species and the highly structured populations of these spiders (i.e. in *T. grallator*[10,29]) makes these systems ideal for examining evolution under balancing selection. Our ultimate aim is to elucidate the molecular basis of the evolutionary changes that have led to the parallel evolution of similar coloration in these species. However, a necessary step in this process is the determination of the pigment synthesis pathways that are present in these spiders and the gene sequences associated with them. Subsequently candidate genes associated with the allelic basis of the color polymorphism or that are differentially expressed among color morphs can be identified. The advent of next-generation sequencing technologies has permitted rapid profiling and *de novo* assembly of the complete set of expressed mRNA sequences in a specific tissue or whole organism (transcriptome sequencing, RNA-seq [30]). In addition to providing information on the structure of expressed gene transcripts (as *de novo* assembled “contigs”), the digital nature of RNA-seq facilitates the determination of both relative transcript expression levels within a tissue or organism and the differential expression of transcripts among tissues or experimental treatments. Using data generated through a combination of RNA-seq and the sequencing of normalized cDNA libraries to compensate for the under-sampling and poor assembly of rarer transcripts, we report on the *near-*complete whole-body expressed transcriptomes of two species of color-polymorphic spider, *Theridion californicum* and *T. grallator*. This represents the most extensive genomic data set for spiders so far available. We report on the gene complement of these species and highlight gene families that appear to have experienced expansion in the lineage leading to spiders. In particular we identify pigment-pathway genes in these spiders and we secondarily examine these, as well as the larger gene set, for evidence of differential expression between the common (double recessive) Yellow (unpatterned) morph and Colored (patterned) morphs.

## Results

### Sequencing and *de novo* assembly of two spider transcriptomes

The transcriptomes of the two spider species, *Theridion grallator* and *T. californicum*, were assembled from a combination of RNA-seq and normalized cDNA Illumina short-read data. The annotated contigs are available as Additional file 1 (*T. californicum* transcriptome) and Additional file 2 (*T. grallator* transcriptome). The trinity based assemblies returned a large number of contigs (or “isotigs”, i.e. transcript models) clustered into a number of components (“genes”) and the numbers of reads and contigs at each assembly step is outlined in Table 1. Although all contigs > 100 bp were retained by trinity, here we report the statistics and counts for all contigs > 200 bp and refer the reader to Tables 1 and 2 for full count information. The assembly for *T. californicum* consisted of 128,391 contigs (>200 bp) in 83,701 components and that for *T. grallator* of 104,481 contigs in 89,166 components. The maximum contig length for *T. californicum* was 24,235 bp and for *T. grallator* was 17,866 bp (both corresponding to twitchin/titin muscle proteins). The mean contig length for *T. californicum* was 606 bp and for *T. grallator* 601 bp and the N50 contig lengths were 901 bp and 926 bp respectively. The frequency distribution of contig lengths for each assembly is given in Additional file 3: Figure S1. The large number of contigs between 100 and 200 bp in length can be assumed to consist of both real short transcripts (that are difficult to annotate by blastx searches since they are so short) and many contigs that represent non-overlapping fragments of single genes - greatly inflating gene counts. The extent of this fragmentation was explored by using the 19,693 genes of the UniprotKB *Drosophila melanogaster* proteome as a target for blastx searches with each of the spider transcriptomes. Of the 4,641 *T. grallator* contigs >100 bp that generated blast hits to *D. melanogaster* genes 2,499 (54%) were unique best hits (i.e. the *D. melanogaster* protein was not the best hit for any additional contigs). When only contigs > 200 bp were considered 2,273 of 3,543 (64.15%) hits were unique. Similarly, for *T. californicum* contigs > 100 bp in length 2,783 of 5,161 (54%) of hits were unique and for contigs > 200 bp, 2,622 of 4,251 (62%) were unique. This increase in the proportion of unique hits (ca. 10%) when contigs 100–199 bp are excluded indicates that contigs of this length are likely highly fragmented.

**Table 1:** ***Theridion californicum *** **and *T. grallator *** **transcriptome sequencing and assembly statistics**

**Input**	***T. californicum***	***T. californicum***	***T. grallator***	***T. grallator***
	**“Yellow”**	**“Colored”**	**“Yellow”**	**“Colored”**
**Initial reads**^**1 **^**RNA-seq**	165,289,830	166,918,608	219,423,001	297,051,726
**Initial reads**^**1 **^**ncDNA**	111,126,430	102,037,432	241,426,000	187,430,684
**Total initial reads**^**1**^	276,416,260	268,956,040	460,849,001	484,482,410
**Preprocessed reads**	141,712,102	109,218,670	88,114,377	151,149,116
**Combined reads**	250,930,772		239,263,493	
**Reads entering assembly**^**2**^	171,894,396		168,943,057	
**Inchworm *K*** **mers**	885,888,079		820,735,751	
**Chrysalis contigs**	5,658,477		6,140,420	
**Butterfly contigs**	128,391 (389,967)^3^		104,481 (459,452)	
**Butterfly components (“Genes”)**	83,701 (295,585)		89,166 (427,020)	
**Mean contig length (bp)**	606 (289)		601 (235)	
**Median contig length (bp)**	344 (152)		332 (130)	
**N50 contig length (bp)**	901 (429)		926 (273)	
**Mean coverage depth (reads)**^**4**^	2284 (1722)		4493 (3001)	
**Median coverage depth (reads)**	99 (33)		329 (45)	
**Maximum transcript length (bp)**	24,235		17,866	

^1^All counts are expressed as “single” reads for comparative purposes.

^2^Subset due to RAM limitations.

^3^Values outside parentheses are for contigs > 200 bp, inside parentheses contigs > 100 bp.

^4^Coverage calculated using RSEM [31].

**Table 2:** ***Theridion californicum *** **and *T. grallator *** **transcriptome annotation and coding gene composition statistics**

	***T. californicum***	***T. grallator***
**blastx** **+ ve**^1^**Transcripts**	43,009 (54,777)	42,538 (76,610)
**blastx** **+ ve Components**	23,586 (33,789)	22,658 (68,541)
**Metazoan (“spider”) blastx** **+ ve Transcripts**	35,411 (47,179)	22,724 (34,062)
**Metazoan (“spider”) blastx** **+ ve Components**	20,611 (28,215)	18,868 (29,397)
**Mean coding contig length (bp)**	1090 (855)	1270 (892)
**Median coding contig length (bp)**	751 (459)	990 (443)
**N50 coding contig length (bp)**	1699 (1628)	1903 (1832)
**Coding transcriptome%GC**	36.43	35.17
**Coding transcriptome size (Mbp)**^**2**^	22.47 – 25.84	23.96 – 27.56

^1^blastx + ve = blastx-positive, i.e. those transcripts or components that had received a positive hit to a known and putatively homologous protein sequence.

^2^Mean transcript size multiplied by number of spider blastx-positive components (genes) – with and without an additional 15% of components based upon Markov-ORF analysis (see text).

### Functional annotation and filtering of putative contaminant organisms

The subset of putative protein-coding transcripts present in the assemblies was identified using two approaches. First, all the transcripts were subject to blastx homology searches against the entire NCBI non-redundant *nr* protein database. For *T. californicum* 43,009 contigs > 200 bp (in 23,586 components) and for *T. grallator* 42,538 contigs > 200 bp (22,658 components) had at least one blast hit with an expected E-value smaller than 1×10^-3^ (Table 2). Examination of the blast hits indicated that a significant proportion of the contigs in both species were likely to originate not from the spider *per se* but from parasitic, commensal and environmental contaminants (the “meta-transcriptome”). The contigs with blastx hits were therefore filtered into two sets based upon the blastx hit species tag, using the program megan 4[32]. All contigs that were assigned to the Metazoa (with the exception of Nematoda – because these species are likely to be infected with nematodes – see Methods) were designated as “spider” contigs and all others “non-spider”. This resulted in a final spider blastx-positive set of 35,411 contigs > 200 bp (20,611 components) for *T. californicum* and 22,724 contigs > 200 bp (18,868 components) for *T. grallator* (Table 2). In other words, 17.67% of the blastx-positive *T. californicum* contigs (>200 bp) are likely not to correspond to spider genes. The same is true of a remarkable 46.58% of *T. grallator* contigs (>200 bp). This resulted in the final number of spider blastx-positive components (“genes”) between the two species being only 8% different (20,611 versus 18,868). Relative abundances and taxonomic distributions of the non-spider taxa at the Phylum level and for bacterial classes are given in Additional file 3: Tables S1 and S2. Most of the non-spider taxa are bacteria or fungi indicating a rich microbiome that is more complex in the tropical species. The blast2go annotation pipeline rejected 1,737 *T. californicum* and 2,521 *T. grallator*blastx-positive contigs because the minimum *hsp* length was shorter than 33 aa. Overall, the E-values of the blastx hits were very low with 42,999 (94.62%) of *T. californicum* and 29,846 (94.63%) of *T. grallator* hits (considering *all* contigs >100 bp) having an E-value <1×10^-5^ (Additional file 3: Figure S2).

The top 20 taxa generating blastx hits to the spider contigs are illustrated in Additional file 3: Figure S3. Although this distribution partly reflects the biased composition of the NCBI *nr* database, 14 of the top 20 taxa were invertebrates, including three arachnids – the deer tick *Ixodes scapularis* (the top-hit taxon being hit by 21.22% of *T. californicum* and 21.13% of *T. grallator* sequences), the Gulf Coast tick *Amblyomma maculatum* (hit by 3.34% of *T. californicum* and 3.91% of *T. grallator* sequences), and the western black-widow spider *Latrodectus hesperus* (hit by 1.75% of *T. californicum* and 2.15% of *T. grallator* sequences). (Annotated protein sequences from the recently sequenced two-spotted spider mite *Tetranychus urticae*[33] were not available in the *nr* database and were therefore not used for annotation here). The overall distributions of the top blast hits were highly similar for both spider species (Additional file 3: Figure S3). The blastx hits were used for mapping the contigs and subsequently assigning gene ontology (GO) annotations using blast2go pro. In total GO annotations were assigned to 32,603 (69.10%) *T. californicum* and 22,825 (67.01%) *T. grallator* contigs (considering *all* contigs >100 bp).

The blastx homology searches (with subsequent filtering through megan 4) indicate the presence of a large protein-coding gene set in the two species – ca. 20,000 genes (*T. californicum*: 20,611; *T. grallator*: 18,868; 28,215 and 29,397 respectively if contigs 100–199 bp are included). Since the public databases currently contain relatively little gene-sequence information for spiders, we also employed a second approach to coding-gene identification using Markov-model prediction based upon geneid[34], as implemented in trinity. Only open reading frames ORFs greater than 100 aa (ca. 300 bp) were considered. This analysis identified a similar number of putative genes to the blastx analyses: 19,328 components (genes) in *T. californicum* and 17,380 components in *T. grallator*. A detailed analysis of the overlap among the various protein-coding gene set predictions is given in Supplemental Section 6, and Additional file 3: Figure S4. The results of the Markov-ORF prediction suggest that the two spider species might have ca. *4.5% more* protein-coding genes than predicted by blastx homology alone – i.e. *at least* 21,495 coding genes. The *protein coding* transcriptome size was estimated to be between ca. 23.43 Mbp to 27.30 Mbp and the GC content is low: *T. californicum* is 36.93% and for *T. grallator* 35.17% (Table 2).

### Comparative genomics and generation of orthologous gene clusters

**Figure 1:**
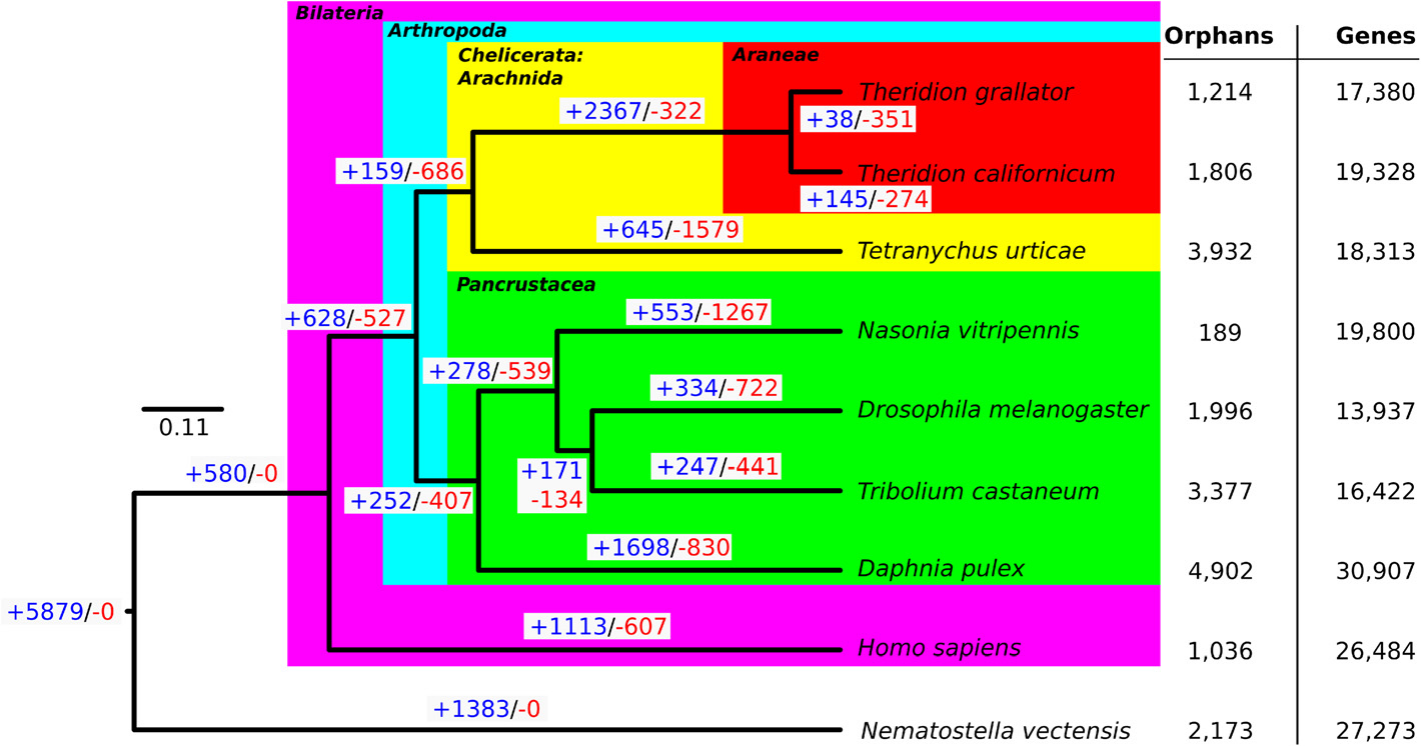
**Gene family gains and losses across the Metazoa.** Dollo parsimony phylogeny of select metazoans derived from complete protein sets, documenting the history of orthologous gene family gains and losses among the metazoans with particular respect to the Araneae. The anemone *Nematostella vectensis* acts as an outgroup for rooting the phylogeny. The number of genes for *T. californicum* and *T. grallator* is from Markov-model-based predictions for ORFs > 100 aa, and therefore excludes many shorter genes.

The most parsimonious reconstruction of gene family gain and loss is presented on the recovered phylogeny in Figure 1. The spider gene data was based upon the sets of Markov-predicted ORFs (see above). The phylogeny was supported by high bootstrap values (all nodes = 100%). Our gene family evolution results are largely congruent with those presented by Grbíc *et al.*[33] in which the genome of the mite *Tetranychus urticae* was described, and any discrepancies are likely due to recent updates of several of the predicted protein datasets. In our analysis, the ancestor of the arachnids had 6033 gene families. The lineage leading to the mite *T. urticae* gained 645 gene families while apparently losing 1,579. *T. urticae* represents the smallest arthropod genome sequenced, at ~90 Mb, and is of atypical size for arachnids. In contrast, the unfinished genome of the tick *Ixodes scapularis*, also a member of the Acari, is much larger (~2,100 Mb) [33]. It is important to note that in these analyses, and also in the pigment-pathway associated gene search, that when we state that a gene (or gene family) was not detected this does not necessarily mean that the gene is absent; it may merely be that we failed to detect the contig because of weak expression, low sequence similarity, lack of expression in adult females, or environment specific expression.

**Figure 2:**
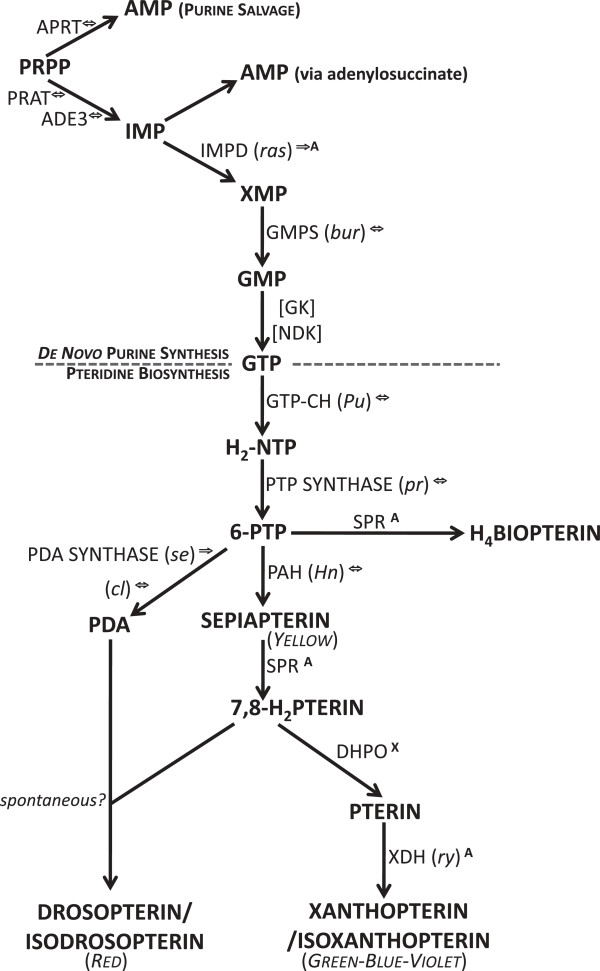
***De novo *purine and pteridine biosynthesis pathways indicating key gene products [**[35-38]**].***Abbreviations:* AMP, adenosine monophosphate; APRT, adenine phosphoribosyl transferase; PRPP, 5-phospho-α-D-ribosyl 1-pyrophosphate; PRAT, phosphoribosylamidotransferase; ADE3, adenosine3 (encodes GARS, glycineamide ribotide synthetase; AIRS, aminoimidazole ribotide synthetase; GART, phosphoribosylglycinamide formyltransferase); IMP, inosine monophosphate; IMPD (*ras*), inosine monophosphate dehydrogenase (*raspberry*); XMP, xanthine monophosphate; GMPS (*bur*), guanine monophosphate synthase (*burgundy*); GMP, guanine monophosphate; GK, guanylate kinase; NDK, nucleoside diphosphate kinase; GTP, guanosine triphosphate; GTP-CH (*Pu*), guanosine triphosphate cyclohydrolase (*Punch*); H_2_-NTP, dihydroneopterin triphosphate; PTP SYNTHASE (*pr*), 6-pyruvoyl-tetrahydropterin synthase (*purple*); 6-PTP, 6-pyruvoyl-tetrahydropterin; SPR, sepiapterin reductase; H_4_Biopterin, tetrahydrobiopterin; PAH (*Hn*), phenylalanine hydroxylase/tetrahydropterin oxidase (*Henna*); 7,8-H_2_PTERIN, dihydropterin; PDA synthase (*se*), pyrimidodiazepine synthase (*sepia*); (*cl*)*,* (*clot*); PDA, pyrimidodiazepine; DHPO, dihydropterin oxidase; XDH (*ry*), xanthine dehydrogenase (*rosy*). *Symbols:***⇔**, supported by reciprocal blast hit, **⇒**, supported by one-way blast hit (*D. melanogaste*r protein versus translated spider transcriptome); **A**, either not supported by reciprocal blast hit or not part of the *D. melanogaster* AmiGO pigment gene set but identified by Blast2GO annotation; **X**, not detected; [], not searched for.

Regarding the Araneae, the lineage leading from the arachnid ancestor to the genus *Theridion* (assuming our two species are representative) accumulated 2,367 novel gene families while apparently only loosing 322. Nearly half of the genes in these novel gene families (45.64% - *T. californicum*; 45.80% - *T. grallator*; and 45.72% - total) could not be assigned to gene ontology (GO) domains (i.e., molecular function, biological process, or cellular component) and did not receive GO annotations. Between the full transcriptomes of the two *Theridion* species sequenced here, 135 unique GO terms were assigned by blast2go. Of these, 131 were present in the *T. californicum* annotations; each of these was shared with *T. grallator*. The *T. grallator* transcriptome contained four unique GO terms: GO:0023033 (signal transduction), GO:0045735 (nutrient reservoir activity), GO:0071568 (UFM 1 conjugating enzyme activity), and GO:0071569 (protein ufmylation). The latter two are associated with the protein UFM 1, a ubiquitin-like protein. The nearly complete overlap of GO annotations between the two species further validates the completeness of our transcriptome data. In order to characterize the spider transcriptome functionally, we explored the level II and level III GO annotations for each of the three GO domains in terms of frequency with which each GO-term was assigned to the dataset. We also included the set of Araneae-specific genes (as defined by the gene-family analysis – Figure 2) in order to attempt to highlight any functional differences that may be enriched within this group. For brevity, these results are discussed in the Supplementary material alongside the accompanying Supplementary Section 13, and Additional file 3: Figures S5-S10.

### Pigment pathway-associated genes in *T. californicum* and *T. grallator*

A principle aim of this study was to identify and characterize expressed pigment-pathway-associated genes in *T. californicum* and *T. grallator*: 1) to clarify which pigment pathways are expressed; and 2), to identify candidate loci responsible for the allelic basis of the color polymorphism. Homologues of known pigment-process-associated proteins from *Drosophila melanogaster* were sought in the full transcriptome assemblies of both *T. californicum* and *T. grallator*. Putative homologues were detected to 59 out of 69 *D. melanogaster* proteins (Tables 3 and 4). Of these, 40 were confirmed as likely homologues by reciprocal best hit (RBH). Again, it is important to note that absence of evidence for a contig is not evidence of absence of a gene. The pigment pathway genes were divided into five broad categories of pathway: heme, melanin, rhodopsin/carotenoid, pteridine and ommochrome . (Tables 3 and 4; details of *D. melanogaster* proteins used for RBH are given in Additional file 3: Table S7).

**Table 3:** **Occurrence of “ *Drosophila *” heme, melanin and rhodopsin pigment-pathway-associated genes in *T. californicum *** **and *T. grallator *** **transcriptome assemblies as identified by reciprocal-blast-** **hit (RBH) analysis**

***Pigment pathway***^**1**^	***Gene symbol***	***Gene name***	***Selected references***	***T. californicum***	***T. grallator***
				***RBH***^**2**^	***RBH***
**Heme**	*Alas*	*Aminolevulinate synthase*	[39,40]	**⇔**	**⇔**
	*CG3156*	*CG3156 (ABC Transporter)*	[39,40]	**⇒**	**⇔**
	*CG3803*	*Heme A synthase*	[39,40]	**⇔**	**⇔**
	*CG5037*	*Protoheme IX farnesyltransferase*	[39,40]	**⇔**	**⇔**
	*Coprox*	*Coproporphyrinogen oxidase*	[39,40]	**⇔**	**⇔**
	*ferrochelatase*	*ferrochelatase*	[39,40]	**⇔**	**⇔**
	*Ho*	*Heme oxygenase*	[41,42]	NA	**⇒**
	*Ppox*	*Protoporphyrinogen oxidase*	[39,40]	**⇔**	**⇔**
	*Updo*	*Uroporphyrinogen decarboxylase*	[39,40]	**⇔**	**⇔**
**Melanin**	*bsk*	*basket*		**⇔**	**⇔**
	*dl*	*dorsal*		**⇔**	**⇔**
	*e*	*ebony*	[43]	**⇒**	**⇒**
	*egr*	*eiger*		NA	NA
	*grim*	*grim*		NA	NA
	*Gr28b*	*Gustatory receptor 28b*		NA	NA
	*hep*	*hemipterous*		**⇔**	**⇔**
	*Hml*	*Hemolectin*	[44]	**⇔**	**⇔**
	*MP1*	*Melanization Protein 1*		**⇒**	**⇒**
	*Nrg*	*Neuroglian*		**⇔**	**⇔**
	*PGRP-LC*	*Peptidoglycan recognition protein LC*		**⇒**	**⇒**
	*Rho1*	*Rho1*		**⇔**	**⇔**
	*Sp7*	*Serine protease 7*		**⇒**	**⇒**
	*Spn27A*	*Serpin 27A*	[45]	**⇒**	**⇒**
	*Spn77Ba*	*Serpin 77Ba*		**⇒**	**⇒**
	*Tl*	*Toll*	[46]	**⇔**	**⇔**
	*y*	*yellow*	[43]	NA	NA
	*yellow-f*	*yellow-f*	[14,47]	NA	NA
	*yellow-f2*	*yellow-f2*	[14,47]	NA	NA
**Rhodopsin**	*Cnx99A*	*Calnexin 99A*		**⇒**	**⇒**
	*CG13611*	*CG13611*		**⇒**	NA
	*Xport*	*exit protein of rhodopsin and TRP*		NA	NA
	*KH1*	*KH1*	[48]	**⇔**	**⇔**
	*ninaA*	*neither inactivation nor afterpotential A*	[49]	**⇔**	**⇔**
	*ninaB*	*neither inactivation nor afterpotential B*		**⇒**	**⇒**
	*ninaD*	*neither inactivation nor afterpotential D*		**⇒**	**⇒**
	*ninaG*	*neither inactivation nor afterpotential G*	[50,51]	**⇒**	**⇒**
	*pinta*	*prolonged depolarization afterpotential (PDA) is not apparent*	[50,51]	**⇒**	**⇒**
	*santa-maria*	*scavenger receptor acting in neural tissue and majority of rhodopsin is absent*	[49]	**⇔**	**⇔**

^**1**^Indicates pigment pathways with which these genes can be broadly associated (see Additional file 3: Table S5).

^**2**^**⇒**: tblastn hit (E < 1×10-5) between the *D. melanogaster* protein and a transcript in the spider transcriptome; **⇔**Reciprocal blast hit (RBH) (blastx, E < 1×10-5) between best tblastn hit and Uniprot-Uniref-100 database; “NA”: No blast hit detected.

**Table 4:** **Occurrence of *Drosophila *** **ommochrome and pteridine pigment-pathway-associated genes in *T. californicum *** **and *T. grallator *** **transcriptome assemblies as identified by reciprocal-blast-hit (RBH) analysis**

***Pigment pathway***^**1**^	***Gene symbol***	***Gene name***	***Selected references***	***T. californicum***	***T. grallator***
				***RBH***^**2**^	***RBH***
**Pteridine**	*ade3*	*adenosine3*	[36]	**⇔**	**⇔**
	*Aprt*	*adenine phosphoribosyl transferase*	[36]	**⇔**	**⇔**
	*Prat*	*Phosphoribosylamidotransferase*	[36]	**⇔**	**⇔**
	*bur*	*burgundy (GMPS)*	[36]	**⇔**	**⇔**
	*ras*	*raspberry*	[36]	**⇒A**	**⇒A**
	*bw*	*brown*^3^	[52,53]	**⇒**	**⇒**
	*cl*	*clot*	[38]	**⇔**	**⇔**
	*DhpD*	*Dihydropterin deaminase*	[14]	NA	NA
	*Hn*	*Henna*	[36,37]	**⇔**	**⇔**
	*mal*	*maroon-like (CG1665)*	[54]	**⇔**	**⇔**
	*Pu*	*Punch*	[36]	**⇔**	**⇔**
	*pr*	*purple*	[36]	**⇔**	**⇔**
	*se*	*sepia*	[35-38]	**⇒**	NA
**Ommochrome**	*cd*	*cardinal*	[55-58]	**⇒**	**⇒**
	*cn*	*cinnabar*	[55]	**⇔**	**⇔**
	*kar*	*karmoisin*	[52,55,59]	**⇔**	**⇔**
	*st*	*scarlet*^3^	[52,53]	**⇒**	**⇒**
	*v*	*vermillion*	[55]	**⇔**	**⇔**
	*w*	*white*^3^	[52,53]	**⇔**	**⇔**
	*z*	*zeste*	[55,56]	NA	NA
	*KFase*	*Kynurenine formamidase*	[52,60]	NA	NA
**Ommochrome & Pteridine “granule group”**	*cm*	*carmine*	[56,61]	**⇒A**	**⇒A**
	*car*	*carnation*	[56,61]	**⇔**	**⇔**
	*ca*	*claret*	[62]	**⇔**	**⇔**
	*or*	*orange*	[57,61]	**⇔**	**⇔**
	*dor*	*deep orange*	[56,61]	**⇔**	**⇔**
	*g*	*garnet*	[56,61]	**⇔**	**⇔**
	*lt*	*light*	[56,61]	**⇔**	**⇔**
	*ltd*	*lightoid*	[63]	**⇔**	**⇔**
	*p*	*pink*	[56,61]	**⇔**	**⇔**
	*rb*	*ruby*	[56,61]	**⇔**	**⇔**

^**1**^Indicates pigment pathways with which these genes can be broadly associated (see Additional file 3: Table S5).

^**2**^**⇒**: tblastn hit (E < 1×10-5) between the *D. melanogaster* protein and a transcript in the spider transcriptome; **⇔**Reciprocal blast hit (RBH) (blastx, E < 1×10-5) between best tblastn hit and Uniprot-Uniref-100 database; 'A’ Verified by Blast2Go pipeline annotation (only if **⇒** or NA).

^**3**^White/Scarlet/Brown complex not distinguishable by RBH.

#### Heme

Of nine *D. melanogaster* heme genes examined, eight were confirmed by RBH. The products of the eight confirmed genes are all involved in heme synthesis [39,40], confirming that the heme pathway, known to be highly-conserved across the tree-of-life [39], is largely intact in these spiders. The gene for *heme oxygenase* (HO) was not detected by RBH; because HO catalyses the degradation of heme into biliverdin [41,42], its apparent absence supports the notion that these spiders do not produce bilin pigments.

#### Melanin

Only seven out of 19 melanin-associated genes (37%) were confirmed by RBH. Melanin pigments have not been reported in spiders [3], although their role in parasite encapsulation in spiders has been assumed [64]. Key genes associated with melanin pigmentation in *D. melanogaster* were not detectable by RBH e.g. *Spn27A*, which regulates the melanization cascade in *D. melanogaste*r [45]; *yellow-f* (dopachrome isomerase) that converts dopachrome to 5,6-dihydroxyindole [14]; nor *ebony* (NBAD-synthase) [43]. The lack of a melanin pigmentation pathway, also implies that spiders do not produce the yellow papiliochrome pigments that are typical of swallowtail butterflies as these depend upon both the melanin and ommochrome pathways [43].

#### Rhodopsin/carotenoid

Although not structurally related, we group rhodopsin and the carotenoid pigments together here simply because rhodopsins are intimately bound to the carotenoid derived cofactor retinal (vitamin A). Only three out of 10 (30%) of the rhodopsin/carotenoid-associated genes were identified by RBH. *Santa-maria* and *ninaA* are important in general carotenoid metabolism [49] and *KH1* contains RNA helicase domains [48].

No genes strongly associated with vision were identified– a testament to the “poor vision” characteristic of most spiders families.

#### Pteridine

Nine (69%) of 13 pteridine-associated genes were identified by RBH (Table 4). Although the pteridine biosynthesis pathway starts with guanosine triphosphate (GTP) as its substrate, the homology search also included key genes from the *de novo* purine synthesis pathway through which GTP is generated (Figure 2) [35-38]. We detected two genes whose products are involved in purine nucleotide salvage: adenine phosphoribosyl transferase, APRT; and hypoxanthine-guanine phosphoribosyltransferase, HGPRT (detected by keyword search; not shown in Figure 2). Genes for all key *de novo* purine synthesis enzymes that were searched for were detected including the classic *D. melanogaster* eye-color loci *raspberry* (inosine monophosphate dehydrogenase, IMPD) (received only one-way blast support but was identified by keyword search against annotations) and *burgundy* (guanine monophosphate synthase, GMPS). Furthermore, all key enzymes leading to the production of H_4_biopterin [27] were detected: *Punch* (guanosine triphosphate cyclohydrolase, GTP-CH (EC 3.5.4.16)), which catalyzes the production of dihydroneopterin triphosphate, H_2_-NTP; *purple* (6-pyruvoyl-tetrahydropterin synthase, PTP-synthase (EC 4.6.1.10)) which eliminates the phosphate groups yielding 6-pyruvoyl-tetrahydropterin, 6-PTP; and sepiapterin reductase (SPR (EC 4.6.1.10)) which yields H_4_ biopterin [36]. The conservation of the H_4_biopterin pathway in spiders is not surprising given that the pathway is shared by plants and animals [14]. However, the detection of the genes *Henna* (phenylalanine hydroxylase/tetrahydropterin oxidase, PAH (EC 1.14.16.1)) [37] and *clot*, a thioredoxin-like protein [38], suggest the possibility that the yellow pigment sepiapterin and orange/red drosopterin pigments could be present. In addition, the gene *maroon-like* was also detected. This encodes a protein with a molybdopterin cofactor sulphurase activity and may regulate the activities of aldehyde oxidase and xanthine dehydrogenase [54].

#### Ommochrome

**Figure 3:**
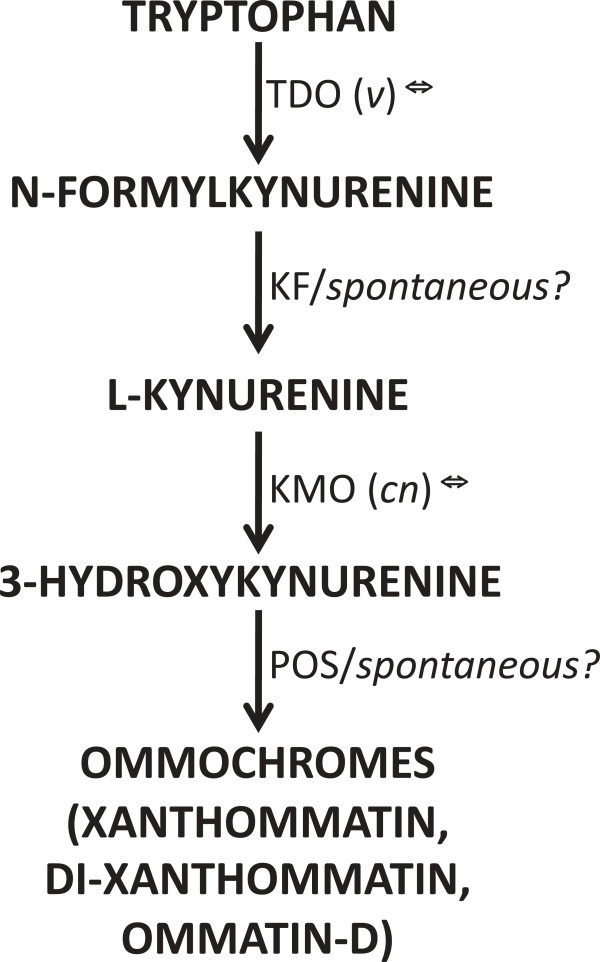
**Ommochrome biosynthesis pathway [**[55]**].** Abbreviations: TDO (*v*), tryptophan 2,3-dioxygenase (*vermilion*); KF, kynurenine formamidase; KMO (*cn*), kynurenine 3-monooygenase (*cinnabar*); POS, phenoxazinone synthase. The genes for KF and POS have not been identified in *Drosophila*.

Of the 18 ommochrome-associated genes that were searched for, 13 were identified Table 4). Neither *cardinal* (which codes for a heme peroxidase) nor *zeste* (which encodes a transcription regulator) [55,56] was detected. The two key enzymes of the ommochrome synthesis pathway *sensu stricto* – *vermillion* (tryptophan 2,3-dioxygenase), and *cinnabar* (kynurenine 3-hydroxylase) (see Figure 3) – were clearly detected. Other enzymes known to be involved, including kynurenine formamidase (KF, KFase) [52,60] and phenoxazinone synthase (POS) [52] were not deteced (although the possibility that the *cardinal* gene may encode for POS has been suggested [56-58]). Overall xthough, our results confirm that the ommochrome pathway is expressed and intact in these spiders.

#### Ommochrome and pteridine transport-associated genes

*ABC-type membrane transporters.* The *white*, *brown* and *scarlet* genes encode subunits of ABC-type membrane transporters. The white and scarlet subunits combine to form an ommochrome precursor transporter and the white and brown subunits combine to form a pteridine precursor transporter [53]. Although the *white* gene was identified by RBH in both spiders, the *brown* and *scarlet* genes were only identified at the level of the one-way blast and therefore their presence cannot be confirmed, although they are likely to be present.

*Tryptophan transport.* The gene *karmoisin* was confirmed by RBH. It has been suggested that the product of this gene, which is probably orthologous to mammalian TAT1, is a tryptophan cell-membrane transporter and is therefore essential to ommochrome biosynthesis [52,55,59].

*Endosomal and vesicle trafficking.* The four subunits of the AP-3 complex (associated with pigment granule formation in invertebrates [56,61]) were all detected by RBH or blast2go annotation: *carmine* (AP-3 subunit *μ*), *garnet* (AP-3 subunit *δ*), *orange* (AP-3 subunit *σ*) and *ruby* (AP-3 subunit *β*). We also detected two genes that encode clathrin heavy-chain peptides: *deep orange* and *light.* In addition we also detected the BLOC-2 component *pink* (HPS5), the HOPS component *carnation*, the Rab GTPase *lightoid* (Rab38) which has been implicated in trafficking to lysosome-related organelles [63], and *claret* – a guanine nucleotide exchange factor that acts with *lightoid*[62].

### Relative and differential expression of genes and isoforms in *T. grallator* and *T. californicum*

The RNA-seq reads for each spider species were mapped back to the assembled transcriptome data so that both overall relative expression levels, and differential expression, of genes could be examined between Yellow and Colored morphs. Of the three most highly expressed genes from each of the two spider species, 5 out of 6 show closest homology to genes from other arachnids and actin is among the most highly expressed genes in both species. (The top 100 expressed genes for each species are given in Additional file 3: Tables S8 (*T. californicum*) and S9 (*T. grallator*)).

Differential expression (DE) between the read pools from Yellow and from Colored individuals was examined by comparing the read mappings between the two groups using edgeR [65-67]. Since no true biological replicates were present in our data, the data sets were normalized against a set of 196 RBH-verified *Drosophila melanogaster* house-keeping (HK) genes. The HK genes were expressed at similar levels in the Yellow and Colored groups of each species. The average absolute difference in the number of reads mapped to the HK genes in the Yellow and Colored categories (as a proportion of the total number of mapped reads) for *T. californicum* was 2.81×10^-5^ reads, and for the *T. grallator* was 2.84×10^-5^ reads. In comparison, the set of pigment-associated genes (see below) had average absolute differences in the number of mapped reads (as a proportion of the total number of mapped reads) of 3.80×10^-3^ for *T. californicum* (135 times that of the HK genes) and 1.86×10^-3^ for *T. grallator* (66 times the HK genes). In order to test for 'statistical significance’ the common dispersion was also estimated using the set of HK genes. Even so, any interpretation of significance in DE among the samples here must be treated with *extreme* caution, especially when the entire transcriptome dataset is considered. The most differentially expressed components for each species are given in Additional file 3: Tables S10 and S11. Nonetheless, our DE analysis suggested that when *T. californicum* Color was compared to *T. californicum* Yellow, 26 components (genes) were “significantly” over-expressed and 19 were under-expressed (*P* < 0.05 after Benjamini-Hochberg false discovery rate correction). When the same comparison was made for *T. grallator,* 356 genes were “significantly” over-expressed and 282 under-expressed. The reason for the discrepancy in the magnitude of these numbers is not clear, however it may well be a simple consequence of fewer individuals entering the sequencing pool for *T. grallator*, generating greater variance in this species’ data. Examination of the differences in GO-term assignment percentages between the entire transcriptome and the DE gene sets (Supplemental Section 26, Additional file 3: Figure S11 and associated text) also revealed little agreement between the two species with respect to DE GO-term enrichment, highlighting the need for caution in interpreting our transcriptome-wide DE assessments.

**Figure 4:**
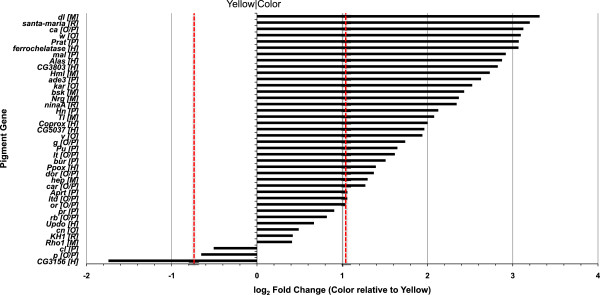
**Differential expression of read-mapped pigment-associated genes in Colored versus Yellow individuals.** Genes plotted in order of decreasing fold-change (log_2_ scale). Red bars indicate +/- 2 S.D. around the mean fold-change (Color versus Yellow) of the house-keeping genes. O = Ommochrome, P = Pteridine, M = Melanin, H = Heme, R = Rhodopsin/Carotenoid. For full gene names see Tables 3 and 4.

The identification of reciprocal homologues among both the *T. grallator* and *T. californicum* HK gene-set and the pigment-associated genes, permitted a more robust analysis for this sub-set of that data than was possible for the transcriptome-wide data. We therefore focused on DE patterns for those pigment genes with measurable expression (RNA-seq) to identify shared changes in expression among Colored versus Yellow samples of both species (Figure 4). The log_2_ fold-change for Color compared to Yellow is plotted in decreasing order from positive to negative. No gene showed statistically significant DE (smallest uncorrected *P-*value = 0.11 for *dl*.). The use of only two (pseudo-) biological replicates yields little statistical power. *In lieu* of “statistical significance” for this data, some confidence in the extent of DE was obtained by examining the standard deviation (SD) in DE among the HK genes; taking any pigment-gene log_2_ fold-change more than or less than 2 SD around the HK mean to be likely to be meaningful (2 SD = -0.75 – 1.04; mean = 0.14; equating to a fold-change < 0.6 or > ~2.1). Of 40 pigment-associated genes examined (Figure 4), three were down-regulated (one by more than 2 SD_HK_) and 37 were up-regulated (30 by more than 2 SD_HK_). Six genes showed a log_2_ fold-change > 3.0 (> 8 fold), most notably these genes included the guanine nucleotide exchange factor *claret* (*ca*) and the ABC transporter *white* (*w*). Both of these proteins are involved in pigment granules formation and trafficking. Another, notably up-regulated gene was *Phosphoribosylamidotransferase* (*Prat*), which is a key enzyme in purine synthesis and is therefore upstream in the pteridine biosynthetic process (see Figure 2).

## Discussion

The transcriptome of each of two species of color-polymorphic theridiid spider was sequenced using Illumina technology and assembled using the assembler trinity. By sequencing pools of individuals at great depth and by combining RNA-seq libraries and sequencing libraries derived from normalized-cDNA (ncDNA) libraries we have been able to reconstruct the transcriptome of each species with apparent completeness. The great utility of RNA-seq data comes from its ability to capture digital gene expression information in the form of relative read coverage. Consequently, RNA-seq is biased towards generating sequence from the most highly-expressed contigs. Since many contigs are likely to be rare, with perhaps less than 1% of expressed genes accounting for 50% of cellular mRNA [68], a typical RNA-seq experiment will fail to record sequence from many transcripts. By using both ncDNA-derived data and RNA-seq data we have been able both to assemble rare transcripts into contigs and *tentatively* examine DE. The contribution of the ncDNA data to the assemblies was clear as only 70-80% of the RNA-seq reads mapped back to the Metazoan blastx-positive components (>100 bp). However, it is also likely that the use of ncDNA resulted in the detection of a large and diverse spider “meta-transcriptome” – an inventory of expressed genes from organisms associated with the spiders (endo- and ectoparasites, commensal and external contaminant organisms). The fascinating discrepancy in the proportion of non-spider sequences between the temperate, mainland species *T. californicum* and the tropical, island species *T. grallator* will be explored elsewhere.

Our transcriptome assemblies are naturally not complete in terms of sampling the full diversity of genes and their various isoforms or in their full-length assembly into contigs. Since the detection of gene transcripts by transcriptome sequencing depends upon the expression of those transcripts, those transcripts that are only expressed at certain life-stages will be missed. Since adult female spiders will contain developing eggs our use of this life stage will naturally also include some transcripts from early development. Accepting the absence of some life-stage specific transcripts, several lines of evidence indicate that our gene sampling is otherwise quite comprehensive. First, the numbers of coding genes predicted, and other characteristics of the assemblies, were consistent between the two species (for example see Table 2 and Figure 1), with the number of Metazoan blastx-positive components (> 200 bp) only differing by 8% (*T. californicum*: 20,611; *T. grallator*: 18,868). Second, the distributions of the top hit taxa and associated E-values (Additional file 3: Figures S2 and S3) from the blastx homology searches, as well as all GO-term assignment analyses (Additional file 3: Figures S5, S7, S9), were remarkably consistent across both species. Furthermore, when GO-terms were assigned to gene families the two species shared 131 of 135 (97.04%) unique GO terms. Third, the CEGMA analysis (Supplementary Sections 8–12, Additional file 3: Tables S3-S6) indicated that 99% (*T. californicum*) and 98% (*T. grallator*) of the 248 CEGs were at least partially represented.

The transcriptomes of *T. californicum* and *T. grallator* contain a large number of contigs that represent components or “genes” (>200 bp: *T. californicum* 83,701; *T. grallator* 89,166; Table 1). These components include both protein-coding genes (whose sequence includes untranslated regions (UTRs) i.e. 5’UTR, 3′UTR, and transcribed introns) and transcribed non-coding sequences. The non-protein-coding genes (i.e. microRNA, ribosomal RNA, transfer RNA, transposons and transposable elements) likely comprise more than 50% of the spider transcriptome but we have not attempted to characterize these here. The set of putative protein-coding components is however impressive and we estimate that these species express *at least* 18,868 (>200 bp) protein-coding genes and probably in excess of 21,495 (>200 bp; perhaps many more if contigs between 100 and 199 bp are considered). *Theridion* spiders, assuming that *T. californicum* and *T. grallator* are representative of the genus, therefore appear to have more protein-coding genes than the well-characterized two-spotted spider mite *Tetranychus urticae* (18,423) and a similar number to *Homo sapiens* (21,828)[69]. For *T. californicum* and *T. grallator* only ca. 4.5% of the Markov-predicted genes (shared among the species and not microbial) had no known homology. Given the large number of Araneae-specific gene families (Figure 1) this low percentage of genes with no known homologues may seem surprising. However, many of these homologues are likely to stem from the fact that the relatively few protein and EST sequences derived from spiders and available in public databases are biased towards those that are specific to spiders i.e. venom and silk gland EST-sequencing experiments (e.g. *Latrodectus hesperus* – see Additional file 3: Figure S3), and venom-gland sequences from other organisms. Of 961 curated venom peptide sequences downloaded from Arachnoserver [70], *T. californicum* had 18 and *T. grallator* had only 14 (23 overall for both species) RBH blast matches to diverse *arachnid* venom peptides (see Additional file 3: Table S12), so if many *Theridion* genes do code for venom peptides then these might be mostly unknown. Until the reads/transcripts can be mapped back to a reference genome it is not possible to be sure about the numbers of *Theridion* genes. Our transcripts are *de novo* assembled and will include erroneously concatenated transcripts and single transcripts that have been split into separate components. Fragmentation is likely to be common for highly-repetitive silk genes, for example and we have demonstrated that short contigs (100–199 bp) are likely to contain many fragments of single genes). However, this is unlikely to detract from the fact that the gene catalogue for these spiders, the first comprehensive list for any spider, is undoubtedly *large*.

In this study, pooling individuals placed a constraint upon our ability to measure DE between the (double recessive) Yellow and (dominant) Colored morphs of these spiders and hence to detect gene pathways associated with the color polymorphism. Without true biological replicates, estimation of the coefficient of variation and hence testing statistical significance becomes impossible. We attempted to circumvent this limitation by borrowing from microarray approaches, normalizing read counts and estimating common dispersion from a defined set of house-keeping (HK) genes. Even so, over such a large set of genes this approach was still of limited utility (as evidenced by the lack of congruence between the two species in terms of numbers of DE genes and enriched GO-terms (Supplemental Sections 23–26, Additional file 3: Tables S10, S11 and Figure S11). Consequently, we chose to focus on the subset of ommochrome- and pteridine-associated genes identified by RBH against *D. melanogaster* homologues in a survey of pigment-pathway associated genes. Since homology was established among the pigment genes and among the HK genes we were able to use the two species as biological replicates, and although statistical power was still weak for significance testing, both species showed a marked and congruent increase in expression in pigment-associated genes in Colored individuals. This result is logical since it is known that the Yellow form is *double recessive* with respect to all the patterned, colored morphs. As such, the recessive Yellow alleles would be expected to show lower expression levels for associated pigment genes when compared to the dominant Color alleles, and this one-tailed expectation is corroborated by both ommonchrome and pteridine pigment pathway genes (Figure 4). These results are also important because they demonstrate that many pigmentation genes are differentially expressed in adult spiders i.e. expression is not restricted to younger instars, perhaps because pigment granules are constantly being cycled [19]. The implication of a role for pteridines in the color polymorphism of these spiders is also very significant because: 1) pteridine pigments have not been described in spiders [3], and 2) because the involvement of this pathway provides an intriguing link between stored guanine and overlying yellow, red and very dark-brown pigments, which have been assumed to be exclusively ommochrome-derived. Together these components interact to generate the various color morphs [6,23]. Of course, the mere presence of the pteridine pathway genes does not necessarily mean that the animals generate pteridine pigments in any appreciable amount, even if it is suggestive of this.

This homology-based approach to pathway-gene identification works because of the deep evolutionary conservation of the pathways associated with the production of many animal pigments. Indeed pigments are often derived from the waste or terminal products of key metabolic processes such as heme [39] and guanine [27], or metabolites generated during the production and recycling of the cofactor H_4_biopterin [14]. Nonetheless, the pathways and the enzymes recruited into various roles do vary and the assumption that spider homologues to *D. melanogaster* enzymes should have equivalent roles is not trivial, especially given that these organisms probably had a last common ancestor some 725 Ma [71].

## Conclusions

We have generated an exhaustive assembly of the transcriptomes of two species of theridiid spider and been able to identify homologues to an array of pigment-pathway genes from *D. melanogaster*. This confirmed the presence of genes from the pathways of known pigments (i.e. ommochromes) and indicated the presence of previously unknown pathways in spiders that may be implicated in the color patterning and polymorphism exhibited by these species (i.e. pteridines). Obvious future work includes the confirmation of the presence these pigments by mass-spectrometry and the verification of putatively differentially expressed genes by qPCR. Our analyses also indicated the likely absence of some pigment pathways. Most notable is the apparent lack of key enzymes associated with melanization in spiders. Although there has been much work on the role of eumelanin in pigmentation and innate defense (encapsulation) in insects and crustaceans [72], this study exemplifies how little is known about innate immunity in spiders (and other non-insect arthropods). Arachnid immunity is likely to be a fruitful avenue of research that, like studies of silk and venom, promises far-reaching medical, agricultural and technological applications. This first comprehensive gene catalogue represents a valuable baseline genomics resource for future research into spider genetics and represents a first and fundamental step towards understanding, and eventually identifying, the genetic basis of the incredible color polymorphism and patterning displayed by these animals.

## Methods

### Samples, RNA extraction, normalization and sequencing

Specimens of *T. californicum* were collected from Albany Hill, Albany, Alameda County, California (37° 54′ N, 122° 20′ W) from beneath the leaves of blackberry plants (*Rubus ursinus*) during the early summer when most individuals are either adult or sub-adult. Specimens of *T. grallator* were collected from Lower Waikamoi Preserve, Haleakala, East Maui, Hawaii (20° 48′ N, 156° 14′ W) from the undersides of leaves of the native *Broussaisia arguta* and *Clermontia arborescens*, and the invasive ginger *Hedychium gardnerianum*. All necessary permits and permissions were obtained and no additional special permissions were required for these species. In order to facilitate the identification of differentially expressed color genes, two sets of animals were collected for each species. Each pool consisted of either the “Yellow” (i.e. unpatterned) morph or a mixture of “Colored” (patterned) morphs. This simple scheme is based upon the fact that in all species studied, the Yellow morph appears to be recessive to all other color morphs [6,9] and a similar scoring scheme has been used previously [8,73]. For *T. californicum* the “Yellow” pool comprised 20 Yellow individuals and the “Colored” pool 20 individuals of the following morphs defined in Oxford [9]: “Red lines” (n = 6), “Black spot” (2), “Black blob” (2), “White” (1), “Red ring A” (4), “Red ring B” (2), “Red stripe A” (3). For *T. grallator* the “Yellow” pool consisted of 2 Yellow individuals and the “Colored” pool 2 “Red front and back” individuals as defined in [7]. All animals were adult females and therefore of a similar size. Individuals were examined to ensure that no mites were present, starved for at least 3 days and then flash frozen at -80°C. Animals were homogenized and total RNA extracted using an RNeasy Mini Kit (Qiagen) according to the manufacturer’s instructions. Five μg of total RNA was used to generate an mRNA-seq library from each sample pool. In addition, and in order to recover the maximum number of genes, 2 μg of total RNA was converted to cDNA using a MINT cDNA synthesis kit and this was subsequently used to generate a normalized cDNA library using the TRIMMER kit (both Evrogen, Moscow, Russia), according to the manufacturer’s instructions. Illumina sequencing libraries were created from 50 ng of each normalized cDNA (ncDNA) pool following the NEXTERA protocol (Evrogen, Moscow, Russia) and paired ends sequenced (50 and 76 bp reads, insert sizes ca. 200–300 bp) on either a Genome Analyzer II or Hi-Seq 2000 sequencer (Illumina).

### Sequence quality assessment, pre-processing and *de novo* assembly

The raw sequence reads were graphically inspected for quality using FastQC v.0.10.0 (Babraham Bioinformatics). Reads were subsequently trimmed to a quality greater than 20 (Phred Score) throughout and adaptor/primer sequences removed using the 'preprocess’ module of String Graph Assembler, SGA [74]. Further trimming of low quality, redundant and polyN sequences was performed using the ShortRead Bioconductor package [75]. In order to recover an assembly that would be both as representative as possible of the full transcript complement and comparable between the color categories (Colored and Yellow), we assembled the transcriptome of each species using all the reads for each species combined (RNA-seq and ncDNA from both Colored and Yellow pools), creating a single read-pool for each species (each ~250 million reads). Due to RAM limitations the number of reads entering the assembly pipeline was subsequently reduced to ~170 million. Each transcriptome was assembled using the *de novo* transcriptome assembler trinity (release 2011-10-29) [76] on a 48 core cluster with 256 GB RAM. The assembly used the default *k*mer size of 25 bp and a minimum contig length of 100 bp.

### Functional annotation and identification of the meta-transcriptome

The complete set of trinity transcripts was assessed for homology by executing local blastx searches against the entire downloaded National Center for Biotechnology Information (NCBI) non-redundant (*nr*) protein database (as of Dec. 29, 2011). All E-values up to 1×10^-3^ (using the PAM30 similarity matrix) were accepted as significant and up to 20 best hits per transcript were retained. All sequences with significant blastx hits were loaded into blast2go pro[77,78] for functional annotation. blast2go was used to manage internet based interproscan (IPS) searches for conserved protein motifs, map enzyme codes, search KEGG pathway maps [79] and to map gene ontology (GO) terms to each sequence.

Percentage assignments of GO terms to the trinity transcripts for the three GO functional domains “cellular component”, “molecular function” and “biological process” were assessed at GO levels II and III. Positive enrichment of particular GO terms (functional classes) (i.e. all transcripts versus spider lineage specific transcripts - see Comparative Genomics, below – and DE genes – also see below) was assessed in two-ways. First, specific GO-terms (only level II and III) within each GO domain were assessed by Bonferroni-corrected contingency-table (χ^2^/Fisher’s exact test) analysis of the scores for each term within each category. Second, positive enrichment was examined using Fisher’s exact tests (FDR significance threshold 0.05) and the directed acyclic graph (DAG)-based enrichment analysis function of blast2go (which was not restricted to levels II and III).

Sequences that were likely to be derived from non-spider contaminants (mainly parasitic, commensal and external contaminant organisms - the spider “meta-transcriptome”), were identified by filtering the blastx results for all putatively non-metazoan transcripts. This was done by mapping the blastx results against the NCBI taxonomy using megan v.4.69.4 [32] with the lowest common ancestor (LCA) algorithm (settings: minimum support = 5; minimum score = 35.0; top percent = 10.0; minimum complexity = 0.3). Putative spider sequences were taken as those mapping to the metazoa, with the exception of a small subset of transcripts that were assigned by megan specifically to the Nematoda (*T. californicum*: 671 transcripts, *T. grallator*: 126 transcripts) as these species are known to be commonly parasitized by mermithid nematodes (PJPC, pers. obs. and [80,81]). All other “non-metazoan” transcripts were therefore deemed part of the meta-transcriptome of the spiders.

In addition to blastx searches, putative protein-coding genes were also detected using a Markov Model-based prediction scheme. Open read frames (ORFs) in each transcriptome assembly were searched using scripts provided by the trinity pipeline. The trinity method essentially implements the ORF prediction methods of geneid[34]. We searched for the 500 longest ORFs in all 6-reading frames (those most likely to represent true spider genes) in each dataset and used these to parameterize a hexamer-based Markov model. The same ORFs were then randomized to generate a null-model for non-coding sequence and all transcripts were then searched for the longest, most-likely coding ORF. This was scored as putatively coding or non-coding according to a likelihood ratio test.

### Comparative genomics and generation of orthologous gene clusters

#### Gene family clustering

Clusters of gene families were created using the predicted proteins of *T. californicum*, *T. grallator* and chosen outgroups with fully sequenced genomes. If isoforms for a gene existed in the predicted peptides of the *Theridion* species, only the longest variant was retained. For outgroup comparisons, the most recent CDS sequences (19 July 2012) were selected from the following taxa with existing genome sequences: *Nematostella vectensis* (Cnidaria: Anthozoa, http://genome.jgi-psf.org/Nemve1/Nemve1.download.ftp.html), *Homo sapiens* (Chordata: Mammalia, http://www.ncbi.nlm.nih.gov/projects/CCDS/CcdsBrowse.cgi), *Daphnia pulex* (Arthropoda: Pancrustacea: Branchiopoda, http://genome.jgi-psf.org/Dappu1/Dappu1.download.ftp.html), *Nasonia vitripennis* (Arthropoda: Pancrustacea: Hexapoda: Hymenoptera, ftp://ftp.hgsc.bcm.edu/Nvitripennis/annotation/), *Trobolium castaneum* (Arthropoda: Pancrustacea: Hexapoda: Coleoptera, ftp://ftp.hgsc.bcm.edu/Tcastaneum/Tcas2.0/annotations/), *Drosophila melanogaster* (Arthropoda: Pancrustacea: Hexapoda: Diptera, ftp://ftp.flybase.net/releases/FB2012_04/dmel_r5.46/fasta/), and *Tetranychus urticae* (Arthropoda: Arachnida: Acari, https://bioinformatics.psb.ugent.be/gdb/tetranychus/). (As noted earlier, *T. urticae* annotated protein sequences were not available in the *nr* database for the earlier (Dec. 29, 2011) annotation stage and do not appear in Figure 2).

#### Phylogenetic inference

Orthologous genes were identified using the hamstr pipeline [82]. hamstr uses hidden Markov models (HMMs) and reciprocal best-hit (RBH) blast searches against a predefined set of orthologous sequences derived from model organisms. The identified orthologs were aligned individually. The programs gblocks[83,84], aliscore[85], and alicut[86] were used to remove poorly aligned and overly “gappy” portions of the alignments. Sequences less than 100 amino acids in length were removed, and any alignments with missing taxa were deleted. The 352 trimmed alignments remaining, comprising 170,965 aligned amino acid sites, were concatenated using fasconcat[87], and a partitioned maximum likelihood (ML) phylogenetic analysis run in the program raxml[88]. The concatenated alignments were partitioned by gene, and each partition was assigned the PROTGAMMA (gamma shaped distribution of site rates with four rate categories) model using the WAG amino-acid-substitution matrix [89]. To find the most likely tree topology, 1000 random addition sequence (RAS) replicates were performed followed by 1000 bootstrap replicates. The “chronopl” command from the R package “ape” [90] was used to create an ultrametric phylogeny via the non-parametric rate-smoothing approach using the raxml tree. The analysis used no fossil or other calibration points, so the branch lengths display time in “evolutionary units” from 0 to 1. The resulting ultrametric phylogeny was used in downstream analyses.

#### Dollo parsimony reconstruction of gene family evolution

To delineate gene families, CDS sequences for all taxa were combined into a single file and a blast-searchable database was created. An all-against-all blast search was performed using an E-value cutoff of 1×10^-05^. Gene families were constructed using mclblastline[91] with an Inflation Factor of 2.0 and other default parameters. Phylogenetic profiles were constructed for all gene families reflecting the presence or absence of each family within the genomes of all taxa. The most parsimonious scenario for the gain and loss of gene families was inferred under the principle of Dollo parsimony. Under this scenario once a complex character, such as a gene family, is lost it cannot be regained. The program dollop in the phylip package [92] was used to reconstruct the ancestral presence and absence of gene families along all branches of the phylogeny.

### Detection of pigment pathway genes

The *de novo*-assembled transcriptome datasets of both spider species were directly searched for pigment-pathway-associated proteins. All *Drosophila melanogaster* proteins from the AmiGO (v.1.8) [93] database under the category “Pigment Metabolic Process” (GO:0042440) (*n* = 68) were downloaded and searched using the tblastn algorithm (E < 1×10^-5^) against blast databases constructed from the transcriptome assemblies of each spider species. Spider transcripts that were returned as “significant” blast hits were then extracted and subject to a reciprocal blastx search (E < 1×10^-5^) against the Uniref 100 non-redundant *Drosophila melanogaster* protein-sequence download from the Uniprot database (release April 2012).

Ommochrome and pteridine/purine *de novo* synthesis-pathway-associated genes/proteins that were not included in this set, or which had failed to be detected by RBH, were directly searched for in the blast2go annotated transcriptome sets (based upon the entire NCBI *nr* database) for each species via non-exact-match keyword searches against the sequence description. The following keywords were employed: spr, sprt, rosy, sepia, xanthine, pterin, pteridine, raspberry, inosine, brown, pyrimidodiazepine synthase, cardinal, carmine, zeste, yellow, white, scarlet, and ebony (melanin/papiliochrome pathway).

### Read mapping, relative and differential expression estimates

In order to estimate the relative expression levels of the components/transcripts, to look for evidence of differential expression (DE) between “Yellow” and “Colored” samples, we mapped the RNA-seq data back to the transcriptome assemblies for each species using rsem[31] and bowtie[94]. This approach takes into account the uncertainty in read-mapping that is present in RNA-seq data due to the presence of multiple isoforms and estimates maximum likelihood abundances. rsem/bowtie mapping was implemented using scripts packaged with the trinity pipeline.

The experimental design used here did not include within species/phenotype biological replicates. This lack of replication places strong limitations on the ability to make statistical inferences with respect to DE since biological and experimental coefficients of variation cannot be estimated. Consequently, estimates of differential expression presented here must be treated cautiously. To facilitate normalization and to calculate a more meaningful estimate of common dispersion, we chose to use a housekeeping (HK) gene approach. We recovered 1197 putative *Drosophila* house-keeping genes – as previously predicted using a naïve Bayes classifier [95] – using the biomart (European Bioinformatics Institute - EBI) search tool. These proteins were downloaded and searched using the tblastn algorithm (E < 1×10^-5^) against blast databases constructed from transcriptome assemblies of each spider species. These proteins returned significant hits to 3063 *T. grallator* and 3507 *T. californicum* transcripts. Only those putative HK genes that hit a *single* component and had positive hits to both species were considered as valid and subjected to reciprocal blastx searches against the complete *nr* database. The final set of HK genes totaled 196 and was used to normalize the Yellow vs. Colored RSEM count data and to estimate common dispersion in the DE software edgeR[65-67]. This procedure was used to examine the entire read-mapped transcriptome datasets. For the subset of pigment-pathway-associated genes, the homologous contigs for each gene among *T. californicum*, *T. grallator* and *D. melanogaster* were known, we therefore looked for DE that was shared between both spider species. This analysis treated Colored *T. californicum* and Colored *T. grallator* as replicates, and Yellow *T. californicum* and *T. grallator* as replicates and was therefore more robust than the transcriptome-wide analyses. In each case significant DE was determined according to the Benjamni-Hochberg False Discovery Rate (FDR).

### Availability of supporting data

The sequence data associated with this study are available from the National Center for Biotechnology Information archives under the following BioProject accession numbers: *T. californicum* PRJNA217181; *T. grallator* PRJNA217184. Additional annotated sequence files are available as Supplemental information to this article (Additional file 1: Theridion californicum transcriptome; Additional file 2: Theridion grallator transcriptome).

## Competing interests

The authors declare that they have no competing interests.

## Authors’ contributions

PJPC collected the specimens, designed the study, carried out the molecular genetics, analyzed and interpreted the data, and drafted the manuscript. MB performed the comparative analyses and helped draft the manuscript. CJW critically reviewed the biochemistry, performed sequence analysis, and helped draft the manuscript. GSO and RGG collected specimens, helped conceive the study, interpret data and critically revised the manuscript. All authors read and approved the final manuscript.

## Supplementary Material

Additional file 1 ***Theridion californicum *** **transcriptome.** Click here for file

Additional file 2 ***Theridion grallator *** **transcriptome.** Click here for file

Additional file 3 Supplemental information. Click here for file
