# Supplementary material for: De novo characterization of the gene-rich transcriptomes of two color-polymorphic spiders, Theridion grallator and T. californicum (Araneae: Theridiidae), with special reference to pigment genes
Source: BMC Genomics. 2013 Dec 8;14:862. doi: 10.1186/1471-2164-14-862 (PMC3878950; doi:10.1186/1471-2164-14-862)
Supplement: Additional file 3 — Supplemental information. [file 1471-2164-14-862-S3.pdf]

Supplemental Information for:

"*De novo* characterization of the gene-rich transcriptomes of two color-polymorphic spiders, *Theridion grallator* and *T. californicum* (Araneae: Theridiidae), with special reference to pigment genes."

By:

Peter J P Croucher[1], Michael Brewer[1], Christopher J. Winchell[2], Geoff S. Oxford[3], Rosemary G. Gillespie[1].

Affiliations:

[1]Department of Environmental Science, Policy and Management, University of California, Berkeley, CA 94720-3114, USA

[2]Department of Molecular and Cell Biology, University of California, Berkeley, CA 94720-200, USA

[3]Department of Biology (E019), University of York, Wentworth Way, Heslington, York YO10 5DD, UK

Contents

1) **Figure S1. Frequency distribution of contig lengths for the *T. grallator* and *T. californicum* transcriptome assemblies** 4

2) **Table S1. Percentage of contigs assigned to non-Metazoan Phyla** 5

3) **Table S2. Percent relative abundances of bacterial classes in the spider RNA-seq data** 5

4) **Figure S2. Distribution of BLASTX E-values** 6

5) **Figure S3. Distribution of top BLASTX hits** 7

6) ***Text:* Overlap among protein-coding gene predictions** 8

7) **Figure S4. Overlap among protein-coding gene predictions** 9

8) **CEGMA analyses of completeness of gene sampling and transcriptome assembly** 10

9) **Table S3. *T. californicum* full Trinity transcriptome assembly CEGMA report** 11

10) **Table S4. *T. californicum* metazoan-only Trinity transcriptome assembly CEGMA report** 12

11) **Table S5. *T. grallator* full Trinity transcriptome assembly CEGMA report** 13

12) **Table S6. *T. grallator* metazoan-only Trinity transcriptome assembly CEGMA report** 14

13) ***Text:* Gene Ontology (GO) Functional Profiles for the *T. californicum* and *T. grallator* transcriptome assemblies and GO Enrichment Analyses for Araneae Specific Gene Families** 15

14) **Figure S5. Distribution of transcripts by Biological Process GO terms** 17

15) **Figure S6. BLAST2GO enrichment analyses for Araneae specific genes relative to total gene set: Biological Process** 18

16) **Figure S7. Distribution of transcripts by Molecular Function GO terms** 19

17) **Figure S8. BLAST2GO enrichment analyses for Araneae specific genes relative to total gene set: Molecular Function** 20

18) **Figure S9. Distribution of transcripts by Cellular Component GO terms** 21

19) **Figure S10. BLAST2GO enrichment analyses for Araneae**

specific genes relative to total gene set: Cellular Component 22

20) Table S7. *D. melanogaster* proteins and reciprocal blast hit (RBH) results for identification of pigment protein genes 23

21) Table S8. Top 100 expressed components ("genes") in *T.californicum* 27

22) Table S9. Top 100 expressed components ("genes") in *T. grallator* 34

23) Table S10. Differentially expressed components ("genes", *n*=45) in *T. californicum* Color relative to Yellow 41

24) Table S11. Differentially expressed components ("genes", Top 100) in *T. grallator* Color relative to Yellow 44

25) Figure S11. Differential expressed GO-terms in Color relative to Yellow 51

26) *Text:* Differential expressed GO-terms in Color relative to Yellow 52

27) Table S12. Venom genes identified by reciprocal best hit (RBH) blast against the Arachnoserver database. 53

28) Table S13. "House-Keeping" genes (*n* = 196) used for DE normalization and estimation of common dispersion 55

29) Literature Cited 69

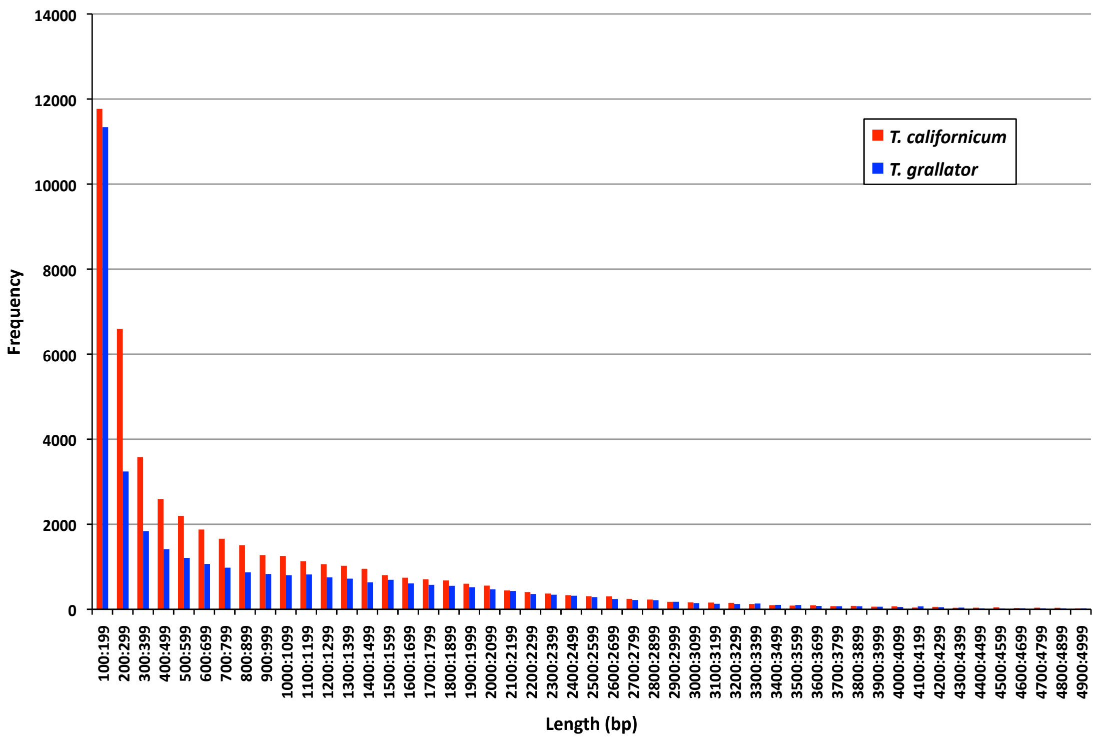

1) Figure S1. Frequency distribution of contig lengths for the *T. grallator* and *T. californicum* transcriptome assemblies. Only contig lengths up to 5,000 bp shown. For *T. californicum* there were 299 contigs longer than 5,000 bp (maximum length 24,235 bp) and for *T. grallator* there were 181 contigs longer than 5,000 bp (maximum length 17,866 bp).

2) Table S1. Percentage of contigs assigned to non-Metazoan Phyla

Phylum	*T. grallator*	*T. californicum*
Bacteria	36.19	7.13
Fungi	13.80	2.19
Viridiplantae	4.07	1.29
Viruses	0.30	0.36
Not assigned	0.28	0.29
Alveolata	0.25	0.48
Amoebozoa	0.18	0.21
Pseudocoelomata	0.16	1.22
Choanoflagellida	0.12	0.30
Stramenopiles	0.11	0.22
Parabasalia	0.10	0.20
Euglenozoa	0.10	0.08
Opisthokonta incertae sedis	0.08	0.14
Archaea	0.03	0.01
Heterolobosea	0.03	0.03
Rhizaria	0.01	0.00
Fornicata	0.00	0.01

Data are presented as percentages of the full set of BLASTX positive TRINITY contigs (*T. grallator* = 76,610; *T. californicum* = 54,777) assigned to Phyla by Megan 4 [1]

3) Table S2. Percent relative abundances of bacterial classes in the spider RNA-seq data

Bacterial Class	*T. grallator*	*T. californicum*
Gammaproteobacteria	16.43	23.50
Mollicutes	13.27	14.20
Betaproteobacteria	13.00	29.56
Clostridia	11.81	2.87
Spirochaetes	6.98	5.42
Bacilli	6.60	0.00
Flavobacteria	6.18	6.45
Fusobacteria	5.65	5.30
Epsilonproteobacteria	5.40	3.40
Alphaproteobacteria	3.69	2.24
Methanococci	2.62	0.80
Thermotogae	1.04	0.19
Sphingobacteria	0.09	1.08
Actinobacteria	0.02	4.61

Raw read data was mapped to a database of bacterial lineage informative genes and relative abundances of bacterial classes computed by the software METAPHLAN [2] . Only bacterial classes with a percent relative abundance > 1.0 are shown.

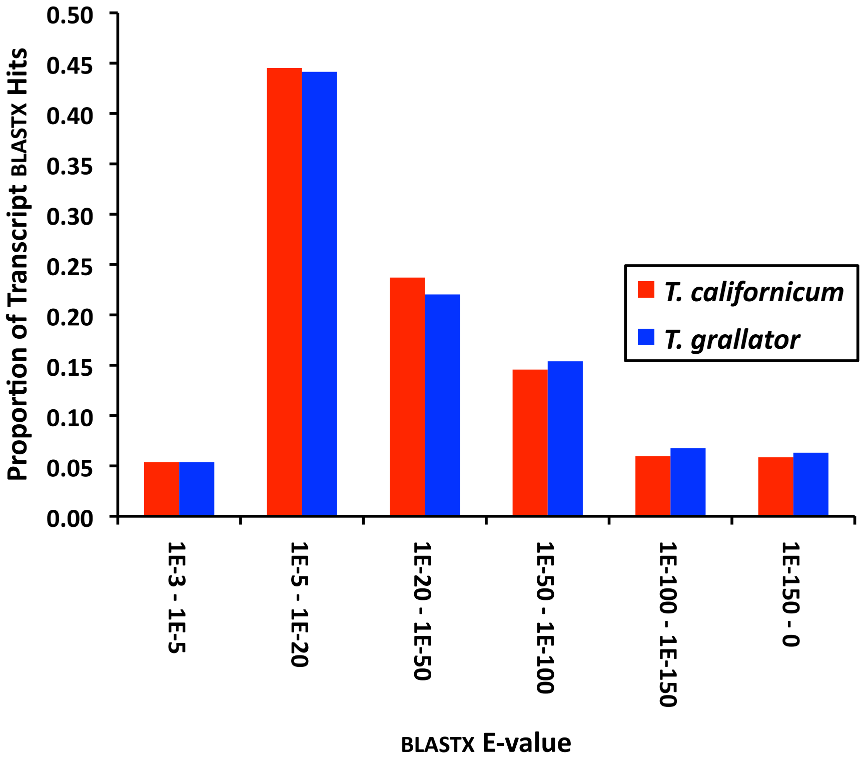

4) Figure S2. Distribution of BLASTX E-values. Histogram showing the distribution of BLASTX E-values among the spider BLASTX-positive transcripts from *T. californicum* and *T. grallator* against the NCBI *nr* database.

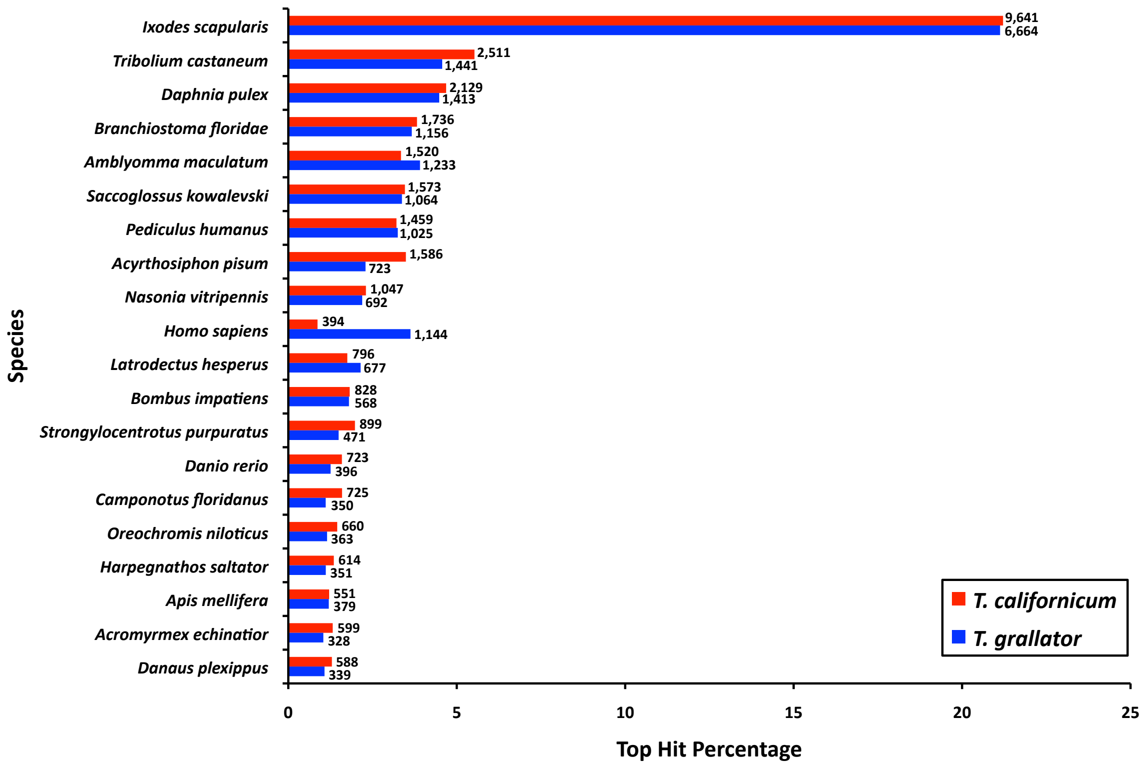

5) Figure S3. Distribution of top BLASTX hits. Taxonomic distribution of top BLASTX hits (E < 1x10^{-3}) of spider BLASTX-positive transcripts from *T. californicum* and *T. grallator* against the NCBI *nr* database. Expressed as a percentage to facilitate comparison; actual counts given next to histogram bars.

6) Over-lap among protein-coding gene predictions.

Venn diagrams in Fig. S4 indicate, for both species, the distribution of contigs among all the spider BLASTX-positive components, non-spider BLASTX-positive components and Markov-ORF prediction components. Of the latter, 2948 (15.25%) *T. californicum* Markov-ORF components contained no BLASTX-positive contigs (i.e. did not belong to either the spider or non-spider BLAST2GO set of components). Similarly, 2488 (14.32%) *T. grallator* Markov-ORF components contained no BLASTX-positive contigs. These may therefore represent novel gene predictions. The proportion of these predicted genes that were likely to be spider genes was evaluated by reciprocal best-hit (RBH) analyses using translating BLAST searches between the two spider species. These indicated that 829 components (28% of *T. californicum* no blastx positive contigs and 33% of *T. grallator* no blastx-positive contigs: E = $1x10^{-3}$; 715 at E = $1x10^{-15}$) were RBH. In other words, approximately two-thirds of these gene predictions in each species were likely to be of non-spider origin. In *T. californicum* 15,242 (78.86%) Markov-ORF components and in *T. grallator* 14,079 (81.10%) Markov-ORF components were also present in the spider BLASTX positive set. In each case the remaining spider BLASTX positive components (i.e. those not Markov-ORF predicted) are largely those containing contigs between 101 and ca. 300 bp in length (39.02% of *T. californicum* and 42.89% of *T. grallator* spider BLASTX-positive contigs) – as Markov-ORF prediction was limited to ORFs >100 aa (i.e. > 300 bp). As discussed above, many of the shorter contigs are likely to be non-overlapping fragments of single genes. *T. californicum* revealed 711 (2.52%), and *T. grallator* 323 (1.10%), BLASTX-positive components that were shared with non-spider BLASTX-positive components. These likely represent "genes" that are conserved between

metazoans and non-metazoans and for which we detected contigs belonging both to spider and contaminating organisms but grouped within the same component. Taken together, the results of the Markov-ORF prediction suggest that the two spider species might have ca. *4.5% more* protein-coding genes than predicted by BLASTX homology alone – i.e. *at least* 20,611 + (829/19,328*20,611) = ca. 21,495 coding genes.

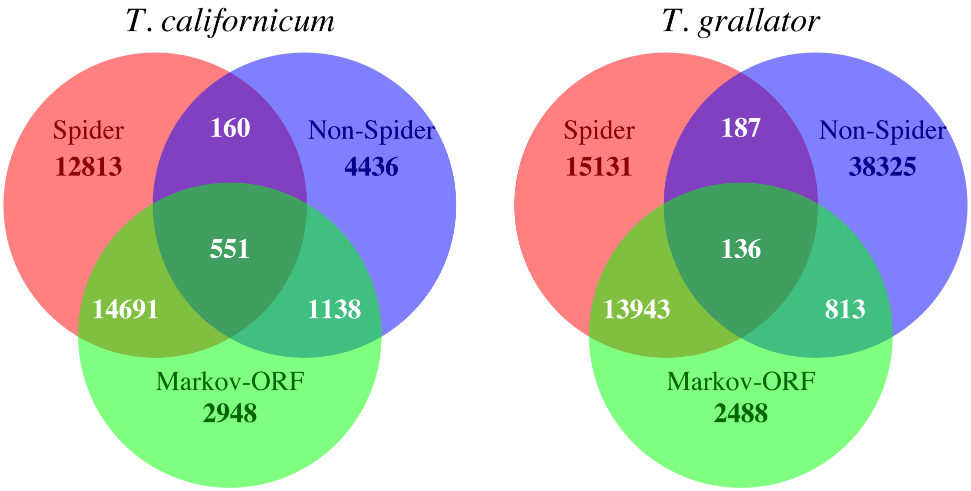

7) Figure S4. Over-lap among protein-coding gene predictions. Venn diagrams illustrating overlaps between sets of putative protein-coding gene components in the assembled transcriptomes of *T. californicum* and *T. grallator*. Three sets of predicted genes are shown – 1) BLASTX homology determined "spider" genes; 2) BLASTX homology determined "non-spider" genes (separated from 1 with MEGAN 4 analyses), and; 3) Markov-model predicted ORFs.

8) CEGMA analyses of completeness of gene sampling and transcriptome assembly.

The similar numbers of genes predicted by the various approaches discussed above, and the similar discrepancies between the approaches, indicate that the gene complement of the transcriptomes of both species have been to a large degree equivalently sampled. An estimate of the completeness of the transcriptome assemblies was made by comparing them to a standard set of 248 core eukaryotic genes (CEGs), derived from the eukaryotic orthologous groups (KOGs) (a subset of the Cluster of Orthologous Groups (COGs) of Proteins database) [3] database using CEGMA [4, 5]. This analysis was performed using both the full TRINITY assemblies and the subset of metazoan-only BLASTX-positive contigs. For *T. californicum* the full transcriptome assembly was found to contain complete (full length) copies of 225 (91%) of the 248 CEGs, with 245 (99%) being at least partially represented. The values did not differ when the analysis was repeated using the subset of metazoan-only contigs. For *T. grallator* the full transcriptome assembly was found to contain complete copies of 237 (96%) CEGs, with 246 (99%) CEGs at least partially represented. When the metazoan-only *T. grallator* contigs were analyzed, these numbers were only slightly reduced to 234 (94%) complete and 243 (98%) at least partially represented CEGs. The transcriptome assemblies therefore recovered most of the CEGs and, although these genes may not be representative of all the genes (in terms of expression for example), these results do give an indication that the assemblies are likely to be quite comprehensive (For detailed CEGMA output see supplementary information Tables S3-S6, below).

CEGMA Completeness Reports for the *T. californicum* and *T. grallator* transcriptome assemblies.

9) Table S3. *T. californicum* full Trinity transcriptome assembly CEGMA report:

```
#      Statistics of the completeness of the genome based on 248 CEGs      #

              #Prots  %Completeness  -  #Total  Average  %Ortho

  Complete      225       90.73      -   427     1.90     52.44

   Group 1       56       84.85      -   114     2.04     62.50
   Group 2       54       96.43      -   110     2.04     57.41
   Group 3       56       91.80      -   111     1.98     50.00
   Group 4       59       90.77      -    92     1.56     40.68

   Partial      245       98.79      -   530     2.16     60.41

   Group 1       64       96.97      -   143     2.23     67.19
   Group 2       56      100.00      -   132     2.36     64.29
   Group 3       61      100.00      -   135     2.21     60.66
   Group 4       64       98.46      -   120     1.88     50.00

#    These results are based on the set of genes selected by Genis Parra   #

#    Key:                                                                  #
#    Prots = number of 248 ultra-conserved CEGs present in genome          #
#    %Completeness = percentage of 248 ultra-conserved CEGs present        #
#    Total = total number of CEGs present including putative orthologs     #
#    Average = average number of orthologs per CEG                         #
#    %Ortho = percentage of detected CEGS that have more than 1 ortholog   #

##              Missing proteins             ##
# Complete
KOG0018, KOG0188, KOG0434, KOG0462, KOG0559, KOG0964, KOG0969, KOG1159,
KOG1299, KOG1322, KOG1349, KOG1535, KOG1596, KOG1760, KOG1774, KOG1795,
KOG1980, KOG2017, KOG2303, KOG2531, KOG3295, KOG3387, KOG3855
# Partial
KOG1159, KOG1535, KOG3387
```

10) Table S4. *T. californicum* metazoan-only Trinity transcriptome assembly CEGMA report:

```
#      Statistics of the completeness of the genome based on 248 CEGs      #

              #Prots  %Completeness  -  #Total  Average  %Ortho

  Complete      225       90.73      -   424     1.88     51.11

   Group 1       56       84.85      -   112     2.00     60.71
   Group 2       54       96.43      -   110     2.04     57.41
   Group 3       56       91.80      -   111     1.98     48.21
   Group 4       59       90.77      -    91     1.54     38.98

   Partial      245       98.79      -   507     2.07     55.92

   Group 1       64       96.97      -   136     2.12     64.06
   Group 2       56      100.00      -   129     2.30     60.71
   Group 3       61      100.00      -   131     2.15     55.74
   Group 4       64       98.46      -   111     1.73     43.75

#    These results are based on the set of genes selected by Genis Parra   #

#    Key:                                                                  #
#    Prots = number of 248 ultra-conserved CEGs present in genome          #
#    %Completeness = percentage of 248 ultra-conserved CEGs present        #
#    Total = total number of CEGs present including putative orthologs     #
#    Average = average number of orthologs per CEG                         #
#    %Ortho = percentage of detected CEGS that have more than 1 ortholog   #

##              Missing proteins            ##
# Complete
KOG0018, KOG0188, KOG0434, KOG0462, KOG0559, KOG0964, KOG0969, KOG1159,
KOG1299, KOG1322, KOG1349, KOG1535, KOG1596, KOG1760, KOG1774, KOG1795,
KOG1980, KOG2017, KOG2303, KOG2531, KOG3295, KOG3387, KOG3855
# Partial
KOG1159, KOG1535, KOG3387
```

11) Table S5. *T. grallator* full Trinity transcriptome assembly CEGMA report:

```
#      Statistics of the completeness of the genome based on 248 CEGs      #

              #Prots  %Completeness  -  #Total  Average  %Ortho

  Complete      237       95.56      -   417     1.76     45.15

   Group 1       64       96.97      -   118     1.84     57.81
   Group 2       50       89.29      -   105     2.10     62.00
   Group 3       59       96.72      -   103     1.75     40.68
   Group 4       64       98.46      -    91     1.42     23.44

   Partial      246       99.19      -   459     1.87     50.41

   Group 1       65       98.48      -   124     1.91     56.92
   Group 2       56      100.00      -   125     2.23     67.86
   Group 3       60       98.36      -   109     1.82     46.67
   Group 4       65      100.00      -   101     1.55     32.31

#    These results are based on the set of genes selected by Genis Parra   #

#    Key:                                                                  #
#    Prots = number of 248 ultra-conserved CEGs present in genome          #
#    %Completeness = percentage of 248 ultra-conserved CEGs present        #
#    Total = total number of CEGs present including putative orthologs     #
#    Average = average number of orthologs per CEG                         #
#    %Ortho = percentage of detected CEGS that have more than 1 ortholog   #

##              Missing proteins            ##
# Complete
KOG0209, KOG0261, KOG0969, KOG1137, KOG1535, KOG1795, KOG2472, KOG2703,
KOG2851, KOG3180, KOG3400
# Partial
KOG0969, KOG1535
```

12) Table S6. *T. grallator* metazoan-only Trinity transcriptome assembly CEGMA report:

```
#      Statistics of the completeness of the genome based on 248 CEGs      #

              #Prots  %Completeness  -  #Total  Average  %Ortho

  Complete      234       94.35      -   427     1.82     48.29

   Group 1       63       95.45      -   119     1.89     61.90
   Group 2       49       87.50      -   108     2.20     63.27
   Group 3       59       96.72      -   111     1.88     45.76
   Group 4       63       96.92      -    89     1.41     25.40

   Partial      243       97.98      -   466     1.92     51.85

   Group 1       64       96.97      -   125     1.95     60.94
   Group 2       55       98.21      -   129     2.35     69.09
   Group 3       60       98.36      -   113     1.88     46.67
   Group 4       64       98.46      -    99     1.55     32.81

#    These results are based on the set of genes selected by Genis Parra   #

#    Key:                                                                  #
#    Prots = number of 248 ultra-conserved CEGs present in genome          #
#    %Completeness = percentage of 248 ultra-conserved CEGs present        #
#    Total = total number of CEGs present including putative orthologs     #
#    Average = average number of orthologs per CEG                         #
#    %Ortho = percentage of detected CEGS that have more than 1 ortholog   #

##              Missing proteins            ##
# Complete
KOG0209, KOG0261, KOG0365, KOG0400, KOG0969, KOG1137, KOG1535, KOG1795,
KOG2472, KOG2703, KOG2770, KOG2851, KOG3180, KOG3400
# Partial
KOG0365, KOG0400, KOG0969, KOG1535, KOG2770
```

13) Gene Ontology (GO) Functional Profiles for the *T. californicum* and *T. grallator* transcriptome assemblies and GO Enrichment Analyses for Araneae Specific Gene Families.

In order to characterize the spider transcriptomes functionally, we explored the level II and level III GO annotations for each of the three GO domains in terms of frequency with which each GO-term was assigned to the dataset (Figs. S5, S7, S9). Again, the similarity in GO-term assignment frequency between the two transcriptomes indicates that the two assemblies are probably very complete. We also included the set of Araneae-specific genes (as defined by the gene-family analysis – main text, Fig. 1) in order to attempt to highlight any functional differences that may be enriched within this group. Spider contigs annotated under the biological process domain (Fig. S5) were heavily biased towards "metabolic process" (GO:0008152), "cellular process" (GO:0009987) and "biological regulation" (GO:0065007). Araneae-specific contigs were significantly enriched in at least one of the species at level II (Fig. S5A) for "developmental process" (GO:0032502), "multicellular organismal process" (GO:0032501), "death" (GO:0016265) and "multi-organism process" (GO:0051704). More specifically at level III (Fig. S5B) these contigs were enriched for "nitrogen compound metabolic process" (GO:0006807), "multicellular organismal development" (GO:0007275), "organelle organization" (GO:0006996), "cell death" (GO:0008219), and "interspecies interactions between organisms" (GO:0044419). Graph-based GO enrichment analysis (Fig. S6) in BLAST2GO indicated enrichment for "symbiosis, encompassing mutualism through parasitism" (GO: 0044403; *T. californicum* False Discovery Rate (FDR) = 0.02; *T. grallator* FDR = NS). This level IV term is a child of GO:0044419, which is itself a child of GO:0051704.

Under the molecular function domain (Fig. S7A and S7B), contigs were biased towards "binding" (GO:0005488) and "catalytic activity" (GO:0003824). At level II the Araneae-specific contigs were heavily enriched for "binding" (GO:0005488) and "transcription regulator activity" (GO:0030528). At level III they were particularly enriched with respect to "protein binding" (GO:0005515), "nucleic acid binding" (GO:0003676), "transcription factor activity" (GO:0003700) and "carbohydrate binding" (GO:0030246). Graph-based BLAST2GO enrichment analysis (Fig. S8) gave some support for enrichment of "carbohydrate binding" (GO:0030246; *T. californicum* FDR = 0.0010; *T. grallator* FDR = NS), "protein binding" (GO:0005515; *T. californicum* FDR = NS; *T. grallator* FDR = 0.0063), and the level V term "nuclease activity" (GO:0004518; *T. californicum* FDR = 0.0010; *T. grallator* FDR = NS)).

Under the cellular component domain (Fig. S9A and S9B) there was some evidence for enrichment in the Araneae-specific contigs at the level II terms "membrane enclosed lumen" GO:0031974 and "extracellular region" GO:0005576 and at the level III term "organelle part" GO:0044422. No enrichment was supported by BLAST2GO enrichment analyses (Fig. S10).

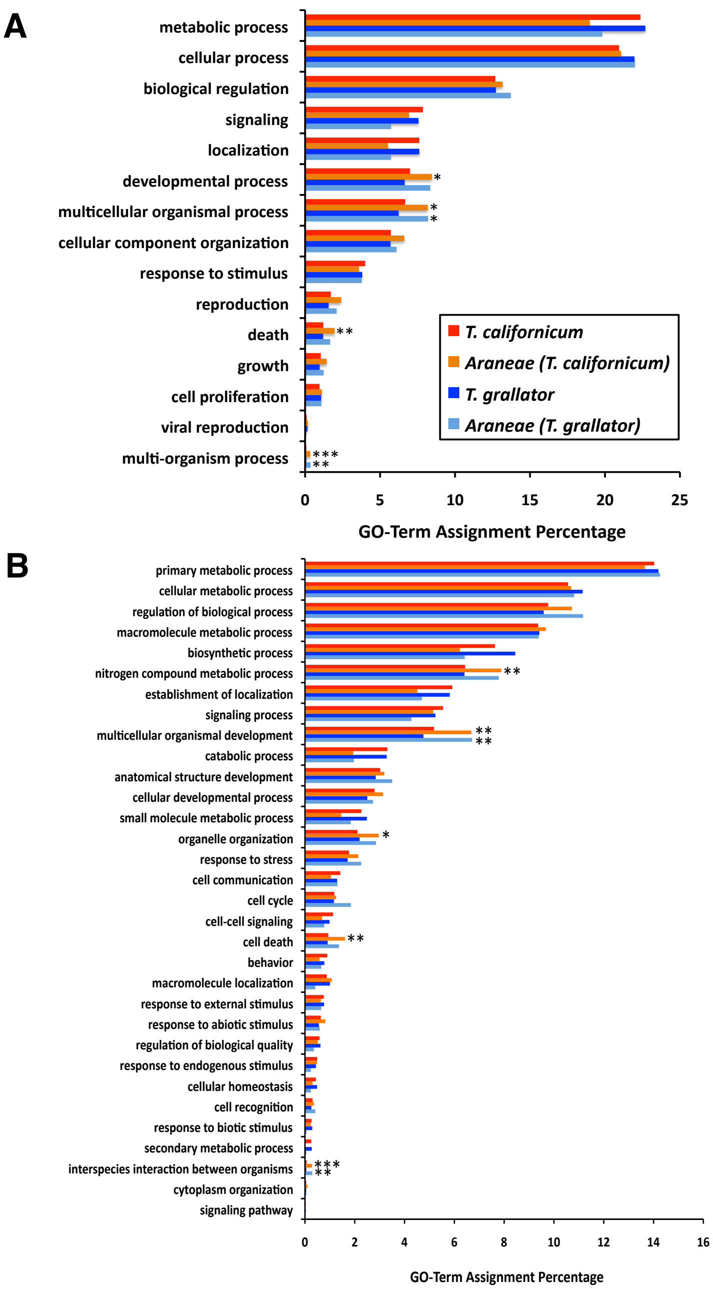

14) Figure S5. Distribution of transcripts by Biological Process GO terms. A) Biological Process level II; **B)** Biological Process level III. Distribution of *T. californicum* and *T. grallator* total transcripts and "spider lineage only" transcripts from orthologous gene family analysis. Significance is based upon actual counts and after Bonferroni correction. * 0.05 > *P* > 0.01; ** 0.01 > *P* > 0.001; *** *P* < 0.001

A) *T. californicum*

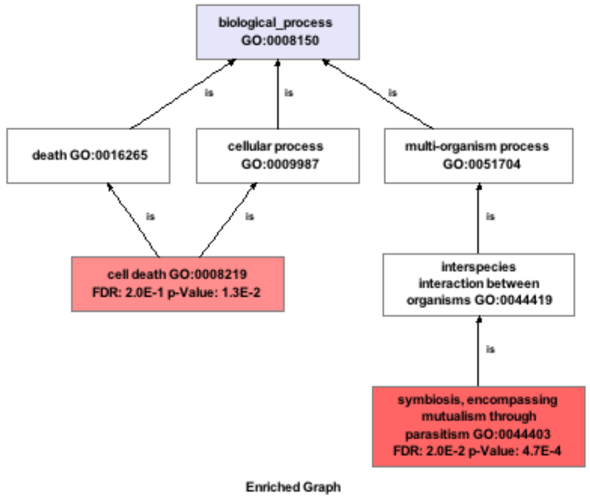

B) *T. grallator*

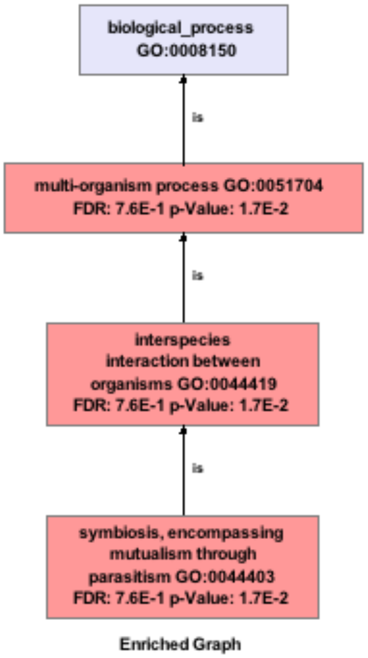

15) Figure S6. BLAST2GO enrichment analyses for Araneae specific genes relative to total gene set: Biological Process.

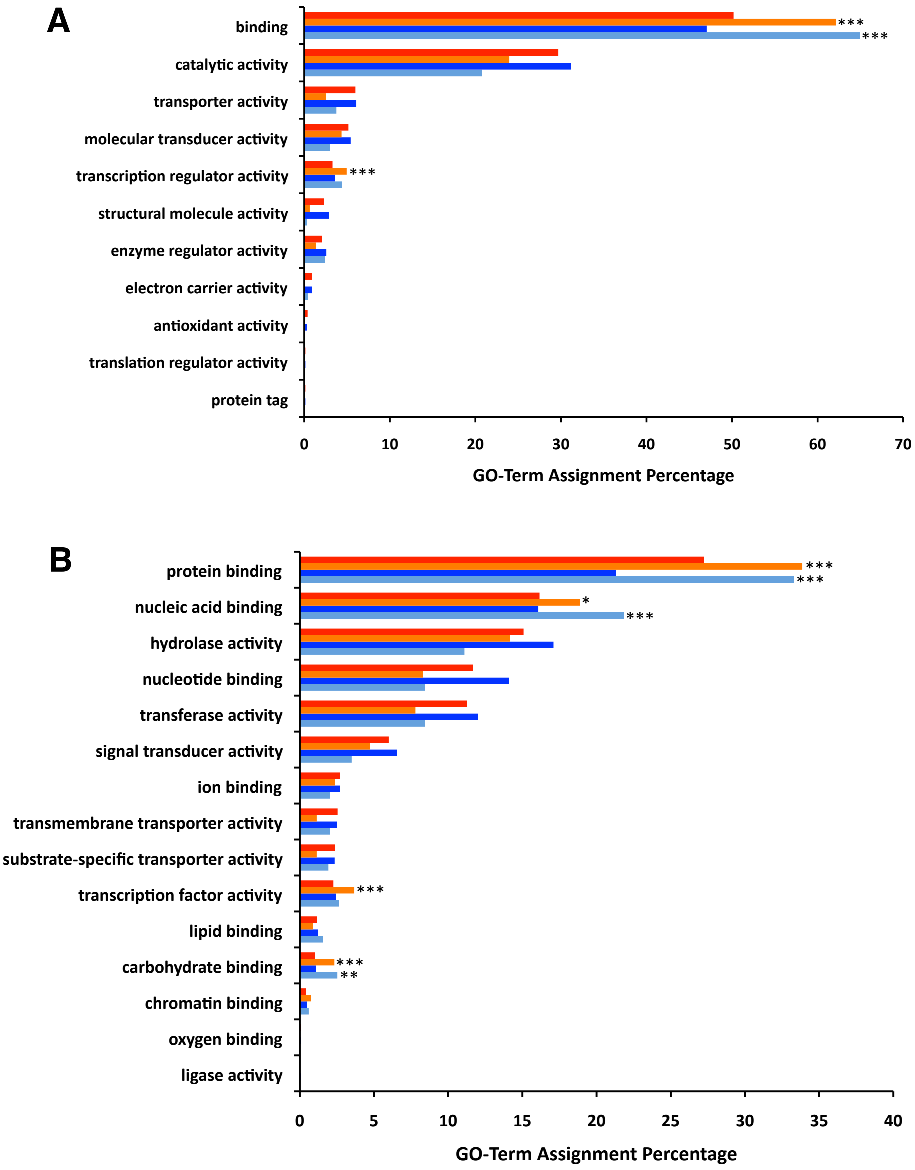

16) Figure S7. Distribution of transcripts by Molecular Function GO terms. A) Molecular Function level II; **B)** Molecular Function level III. For further details see Fig. S5.

A) *T. californicum*

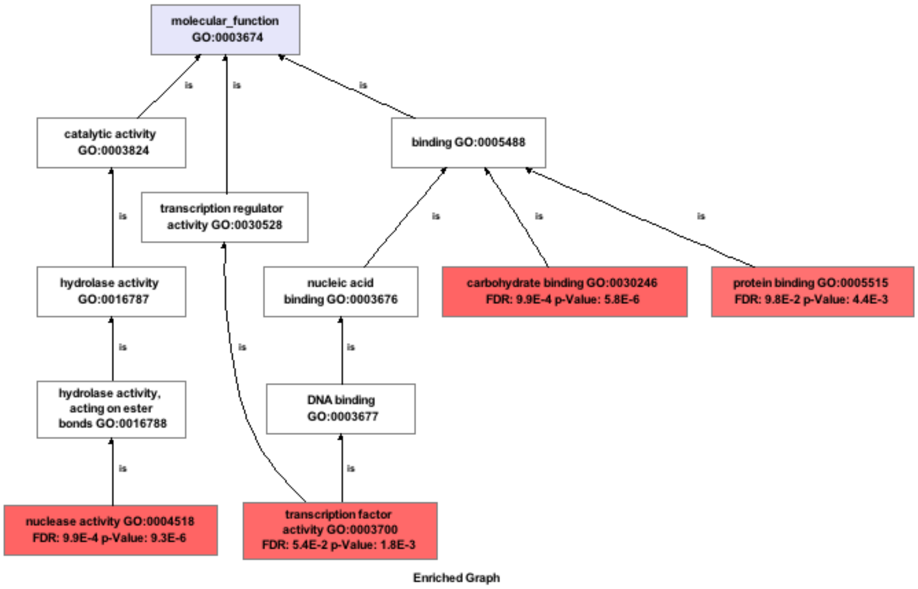

B) *T. grallator*

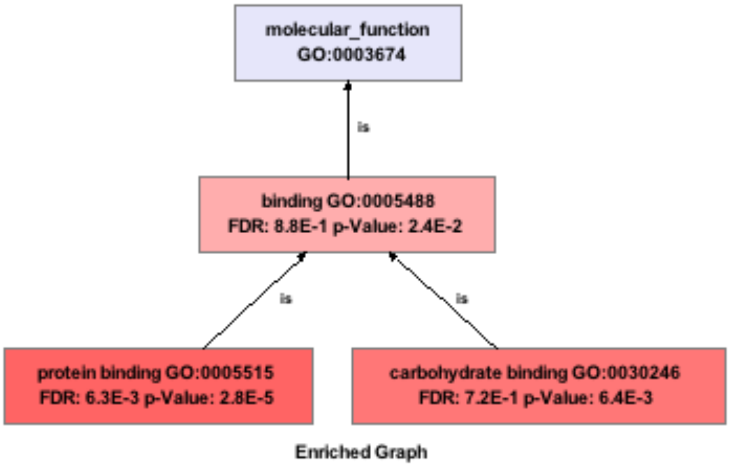

17) Figure S8. **BLAST2GO enrichment analyses for Araneae specific genes relative to total gene set: Molecular Function:**

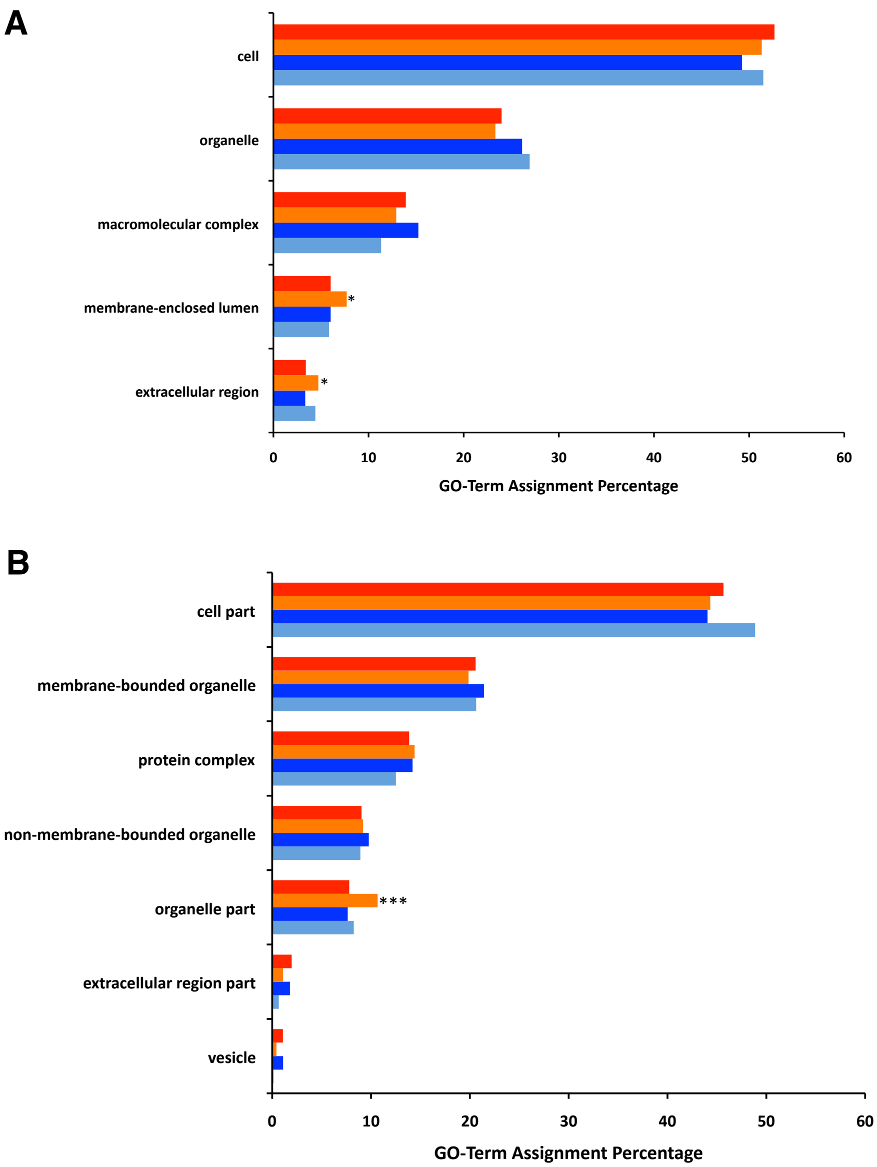

18) Figure S9. Distribution of transcripts by Cellular Component GO terms. A) Cellular Component level II; **B)** Cellular Component level III. For further details see Fig. S5.

T. californicum

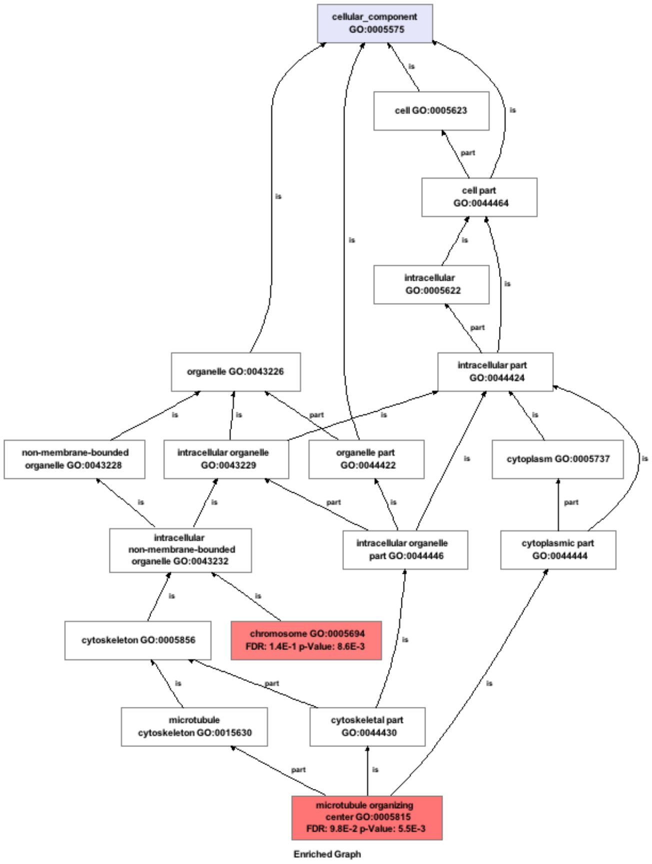

19) Figure S10. BLAST2GO **enrichment analyses for Araneae specific genes relative to total gene set: Cellular Component.** Results for *T. californicum* shown (Not significant after application of FDR). For *T. grallator* no enrichment was detected before or after application of FDR.

20) Table S7. ***D. melanogaster*** **proteins and reciprocal BLAST hit (RBH) results for identification of pigment protein genes**

#	Gene symbol	Flybase identifier	TBLASTN best hit *T. californicum* transcript	TBLASTN E-value	Reciprocal BLASTX best hit species	BLASTX E-value	TBLASTN best hit *T. grallator* transcript	TBLASTN E-value	Reciprocal BLASTX best hit species	BLASTX E-value
1	*Aprt*	FBgn0000109	comp4283_c0_seq1	2.16E-34	*Danio rerio*	6.00E-39	comp649_c0_seq1	6.72E-35	*Danio rerio*	1.10E-38
2	*ade3*	FBgn0000053	comp4286_c0_seq1	1.52E-135	*Drosophila melanogaster*	3.90E-177	comp7355_c0_seq1	6.03E-134	*Rattus norvegicus*	2.90E-145
3	*Alas*	FBgn0020764	comp3731_c0_seq1	0.00E+00	*Drosophila melanogaster*	1.40E-157	comp6728_c0_seq1	4.67E-114	*Bos taurus*	7.90E-92
4	*bsk*	FBgn0000229	comp11143_c0_seq2	0.00E+00	*Drosophila melanogaster*	3.90E-170	comp29637_c0_seq1	0	*Drosophila melanogaster*	5.60E-172
5	*bur*	FBgn0000239	comp659_c0_seq1	0.00E+00	*Rattus norvegicus*	1.10E-236	comp1698_c0_seq1	0	*Rattus norvegicus*	1.20E-239
6	*Cnx99A*	FBgn0015622	comp9161_c0_seq1	9.90E-106	*NA*	NA	comp7585_c0_seq1	1.23E-110	*NA*	NA
7	*cd*	FBgn0263986	comp7526_c0_seq1	8.10E-90	*NA*	NA	comp7938_c0_seq1	2.78E-106	*NA*	NA
8	*cm*	FBgn0000330	comp8035_c0_seq1	0.00E+00	*NA*	NA	comp10417_c0_seq1	0.00E+00	*NA*	NA
9	*car*	FBgn0000257	comp8026_c0_seq1	9.51E-132	*Homo sapiens*	4.10E-191	comp5821_c0_seq1	1.11E-130	*Homo spaiens*	1.40E-190
10	*CG13611*	FBgn0039177	comp1002_c0_seq1	0.000406967	*NA*	NA	NA	NA	*NA*	NA
11	*CG3156*	FBgn0023536	comp6410_c0_seq2	1.42E-88	*NA*	NA	comp38092_c0_seq1	0.00E+00	*Drosophila melanogaster*	1.50E-143
12	*CG3803*	FBgn0034938	comp6069_c0_seq1	4.90E-119	*Drosophila melanogaster*	4.30E-100	comp10873_c0_seq1	9.71E-120	*Drosophila melanogaster*	1.10E-101
13	*CG5037*	FBgn0032222	comp5148_c0_seq1	1.73E-119	*Drosophila melanogaster*	1.70E-89	comp6707_c0_seq1	2.79E-121	*Drosophila melanogaster*	2.80E-85
14	*cn*	FBgn0000337	comp6051_c0_seq1	2.15E-161	*Drosophila melanogaster*	3.00E-124	comp2370_c0_seq1	2.13E-159	*Drosophila melanogaster*	1.20E-122
15	*ca*	FBgn0000247	comp13617_c0_seq1	1.92E-35	*Drosophila melanogaster*	2.90E-44	comp117818_c0_seq1	1.56E-22	*Drosophila melanogaster*	1.70E-19
16	*cl*	FBgn0000318	comp10853_c0_seq1	3.34E-30	*Drosophila melanogaster*	8.50E-24	comp9908_c0_seq2	1.72E-29	*Drosophila melanogaster*	8.50E-24
17	*Coprox*	FBgn0021944	comp6440_c0_seq1	2.15E-171	*Drosophila melanogaster*	8.50E-127	comp13479_c0_seq1	2.08E-172	*Drosophila melanogaster*	6.70E-127
18	*dor*	FBgn0000482	comp8889_c0_seq1	2.95E-155	*Drosophila melanogaster*	1.10E-133	comp11377_c0_seq2	3.91E-151	*Drosophila melanogaster*	6.30E-134
19	*DhpD*	FBgn0261436	NA	NA	*NA*	NA	NA	NA	*NA*	NA

Continued ->

Table S7 (Continued):

#	Gene symbol	Flybase identifier	TBLASTN best hit *T. californicum* transcript	TBLASTN E-value	Reciprocal BLASTX best hit species	BLASTX E-value	TBLASTN best hit *T. grallator* transcript	TBLASTN E-value	Reciprocal BLASTX best hit species	BLASTX E-value
20	*dl*	FBgn0260632	comp24122_c0_seq1	8.67E-97	*Drosophila melanogaster*	2.00E-77	comp41843_c0_seq1	1.20E-103	*Drosophila melanogaster*	3.10E-77
21	*e*	FBgn0000527	comp4967_c0_seq1	1.89E-13	*NA*	NA	comp30447_c0_seq1	3.46E-12	*NA*	NA
22	*egr*	FBgn0033483	NA	NA	*NA*	NA	NA	NA	*NA*	NA
23	*Xport*	FBgn0038749	NA	NA	*NA*	NA	NA	NA	*NA*	NA
24	*ferro-chelatase*	FBgn0024891	comp650_c0_seq1	1.38E-160	*Drosophila melanogaster*	9.60E-119	comp16863_c0_seq2	4.48E-161	*Drosophila melanogaster*	3.60E-119
25	*g*	FBgn0001087	comp11692_c0_seq1	0.00E+00	*Drosophila melanogaster*	8.60E-272	comp12464_c0_seq1	0	*Drosophila melanogaster*	2.20E-275
26	*grim*	FBgn0015946	NA	NA	*NA*	NA	NA	NA	*NA*	NA
27	*Gr28b*	FBgn0045495	NA	NA	*NA*	NA	NA	NA	*NA*	NA
28	*Ho*	FBgn0037933	NA	NA	*NA*	NA	comp8405_c0_seq1	7.50E-41	*NA*	NA
29	*hep*	FBgn0010303	comp26362_c0_seq1	1.13E-128	*Drosophila melanogaster*	1.20E-106	comp26748_c0_seq1	1.08E-127	*Drosophila melanogaster*	5.60E-109
30	*Hml*	FBgn0029167	comp37000_c0_seq1	4.53E-38	*Drosophila melanogaster*	9.90E-43	comp53683_c0_seq1	1.59E-53	*Drosophila melanogaster*	6.20E-57
31	*Hn*	FBgn0001208	comp2896_c0_seq1	0.00E+00	*Drosophila melanogaster*	8.00E-163	comp3994_c0_seq1	0	*Drosophila melanogaster*	3.80E-163
32	*kar*	FBgn0001296	comp2948_c0_seq1	3.44E-99	*Drosophila melanogaster*	4.40E-98	comp5099_c0_seq1	1.69E-101	*Drosophila melanogaster*	5.80E-94
33	*KFase*	FBgn0031821	NA	NA	*NA*	NA	NA	NA	*NA*	NA
34	*KH1*	FBgn0086130	comp12041_c0_seq1	1.54E-43	*Drosophila melanogaster*	2.10E-46	comp9522_c0_seq1	2.09E-67	*Drosophila melanogaster*	1.10E-65
35	*lt*	FBgn0002566	comp6120_c0_seq1	1.58E-169	*Drosophila melanogaster*	5.60E-135	comp10130_c0_seq1	1.02E-176	*Gallus gallus*	1.50E-200
36	*ltd*	FBgn0002567	comp979_c0_seq1	1.03E-96	*Drosophila melanogaster*	2.60E-75	comp4097_c0_seq1	1.25E-94	*Drosophila melanogaster*	1.60E-73
37	*mal*	FBgn0002641	comp3635_c0_seq1	2.03E-11	*Drosophila melanogaster*	1.80E-29	comp19103_c0_seq1	3.29E-07	*Homo spaiens*	6.40E-51
38	*MP1*	FBgn0027930	comp14114_c0_seq1	1.19E-38	*NA*	NA	comp8405_c0_seq1	7.50E-41	*NA*	NA

Continued ->

Table S7 (Continued):

#	Gene symbol	Flybase identifier	TBLASTN best hit *T. californicum* transcript	TBLASTN E-value	Reciprocal BLASTX best hit species	BLASTX E-value	TBLASTN best hit *T. grallator* transcript	TBLASTN E-value	Reciprocal BLASTX best hit species	BLASTX E-value
39	*ninaA*	FBgn0002936	comp1819_c0_seq2	6.91E-51	*Gallus gallus*	2.30E-53	comp3656_c0_seq1	1.32E-51	*Gallus gallus*	8.10E-52
40	*ninaB*	FBgn0002937	comp4415_c0_seq1	1.80E-86	*NA*	NA	comp7709_c0_seq1	3.75E-84	*NA*	NA
41	*ninaD*	FBgn0002939	comp3489_c0_seq1	6.77E-74	*NA*	NA	comp6647_c0_seq1	9.83E-73	*NA*	NA
42	*ninaG*	FBgn0037896	comp19135_c0_seq1	9.35E-61	*NA*	NA	comp8585_c0_seq1	3.83E-58	*NA*	NA
43	*Nrg*	FBgn0002968	comp23885_c0_seq1	5.89E-161	*Drosophila melanogaster*	3.50E-130	comp25842_c0_seq1	1.98E-159	*Drosophila melanogaster*	1.20E-129
44	*or*	FBgn0003008	comp17870_c0_seq1	2.06E-115	*Drosophila melanogaster*	1.40E-85	comp77175_c0_seq1	9.16E-121	*Drosophila melanogaster*	1.30E-85
45	*PGRP-LC*	FBgn0035976	comp63897_c0_seq1	1.34E-22	*NA*	NA	comp11860_c0_seq1	1.01E-21	*NA*	NA
46	*Prat*	FBgn0004901	comp1423_c0_seq1	0.00E+00	*Drosophila melanogaster*	4.00E-161	comp17699_c0_seq1	5.12E-114	*Drosophila melanogaster*	8.30E-95
47	*Prat2*	FBgn0041194	comp1423_c0_seq1	0.00E+00	*NA*	NA	comp17699_c0_seq1	8.31E-119	*NA*	NA
48	*p*	FBgn0086679	comp68395_c0_seq1	7.77E-18	*Drosophila melanogaster*	1.50E-13	comp16974_c0_seq2	1.26E-13	*Drosophila melanogaster*	5.00E-12
49	*pinta*	FBgn0038966	comp44272_c0_seq2	1.20E-16	*NA*	NA	comp26801_c0_seq1	1.72E-16	*NA*	NA
50	*Ppox*	FBgn0020018	comp14635_c0_seq1	5.63E-77	*Drosophila melanogaster*	2.00E-70	comp16016_c0_seq1	7.36E-87	*Drosophila melanogaster*	2.40E-70
51	*Pu*	FBgn0003162	comp5154_c0_seq1	5.66E-86	*Drosophila melanogaster*	1.70E-73	comp4552_c0_seq3	1.50E-89	*Drosophila melanogaster*	9.10E-74
52	*pr*	FBgn0003141	comp12541_c0_seq1	1.30E-43	*Drosophila melanogaster*	3.90E-35	comp8830_c0_seq7	1.86E-23	*Drosophila melanogaster*	7.60E-18
53	*ras*	FBgn0003204	comp2047_c0_seq1	0.00E+00	*NA*	NA	comp2437_c0_seq1	0.00E+00	*NA*	NA
54	*Rho1*	FBgn0014020	comp1577_c0_seq1	3.63E-118	*Drosophila melanogaster*	1.00E-91	comp5740_c0_seq1	4.73E-117	*Drosophila melanogaster*	9.50E-92
55	*rb*	FBgn0003210	comp10775_c1_seq1	0.00E+00	*Drosophila melanogaster*	1.50E-274	comp10948_c0_seq1	0	*Drosophila melanogaster*	6.60E-252
56	*st*	FBgn0003515	comp8034_c0_seq1	1.65E-122	*NA*	NA	comp2133_c0_seq1	1.02E-66	*NA*	NA

Continued ->

Table S7 (Continued):

#	Gene symbol	Flybase identifier	TBLASTN best hit *T. californicum* transcript	TBLASTN E-value	Reciprocal BLASTX best hit species	BLASTX E-value	TBLASTN best hit *T. grallator* transcript	TBLASTN E-value	Reciprocal BLASTX best hit species	BLASTX E-value
57	*santa-maria*	FBgn0025697	comp3489_c0_seq1	5.50E-86	*Drosophila melanogaster*	6.90E-77	comp6647_c0_seq1	3.61E-88	*Drosophila melanogaster*	1.30E-78
58	*se*	FBgn0086348	comp80046_c0_seq1	4.33E-21	*NA*	NA	NA	NA	*NA*	NA
59	*Sp7*	FBgn0037515	comp24146_c0_seq1	2.49E-39	*NA*	NA	comp17750_c0_seq1	1.27E-43	*NA*	NA
60	*Spn27A*	FBgn0028990	comp581_c1_seq1	3.48E-37	*NA*	NA	comp1338_c0_seq1	1.36E-30	*NA*	NA
61	*Spn77Ba*	FBgn0262057	comp6639_c0_seq1	3.40E-32	*NA*	NA	comp1338_c0_seq1	1.86E-32	*NA*	NA
62	*Tl*	FBgn0262473	comp38185_c0_seq1	2.11E-52	*Drosophila melanogaster*	1.20E-49	comp38354_c0_seq1	1.48E-52	*Drosophila melanogaster*	8.00E-46
63	*Updo*	FBgn0033428	comp6172_c0_seq1	5.41E-140	*Drosophila melanogaster*	4.70E-104	comp399_c0_seq1	2.74E-126	*Drosophila melanogaster*	5.90E-96
64	*v*	FBgn0003965	comp3141_c0_seq1	4.09E-136	*Drosophila melanogaster*	5.00E-104	comp4017_c0_seq1	4.14E-134	*Drosophila melanogaster*	3.60E-112
65	*w*	FBgn0003996	comp8034_c0_seq1	1.73E-175	*Drosophila melanogaster*	5.40E-146	comp29235_c0_seq2	1.07E-64	*Drosophila melanogaster*	1.10E-74
66	*y*	FBgn0004034	NA	NA	*NA*	NA	NA	NA	*NA*	NA
67	*yellow-f*	FBgn0041710	NA	NA	*NA*	NA	NA	NA	*NA*	NA
68	*yellow-f2*	FBgn0038105	NA	NA	*NA*	NA	NA	NA	*NA*	NA
69	*z*	FBgn0004050	NA	NA	*NA*	NA	NA	NA	*NA*	NA

NA = No BLAST hit detected.

21) Table S8. Top 100 expressed components ("genes") in *T. californicum*

#	*T. californicum* component	No. of mapped reads	Top BLASTX hit species	Sequence Description	Sequence Length (bp)	Top BLASTX hit E-vlaue	Mean similarity to top BLASTX hit
1	comp72_c0	862786	*Tityus discrepans*	hypothetical protein [Tityus discrepans]	1694	1.83E-05	61.00%
2	comp8_c0	626288	*Agelena silvatica*	actin	1363	0.00E+00	98.15%
3	comp96_c0	463086	*Bos taurus*	hypothetical protein BOS_23236 [Bos taurus]	4574	7.83E-35	72.50%
4	comp6_c0	366887	*Latrodectus hesperus*	hypothetical protein [Latrodectus hesperus]	297	1.40E-09	77.00%
5	comp27_c0	250770	*Haemaphysalis longicornis*	hemelipoglycoprotein precursor	263	1.12E-06	60.78%
6	comp143_c0	232004	*Blomia tropicalis*	blot 13 allergen	762	1.53E-37	70.35%
7	comp84_c0	191464	*Medicago truncatula*	senescence-associated protein	1840	5.91E-67	72.95%
8	comp86_c0	186929	*Uloborus diversus*	beta-actin	711	7.49E-57	100.00%
9	comp20_c0	177120	*Argiope aurantia*	astacin-like peptidase p16	160	1.61E-05	72.00%
10	comp104_c0	163175	*Avicularia avicularia*	myosin regulatory light chain smooth muscle isoform	1193	3.97E-75	74.50%
11	comp0_c0	160258	*Latrodectus hesperus*	conserved plasmodium protein	1608	6.21E-64	61.83%
12	comp13_c0	158882	*Phoneutria nigriventer*	U24-ctenitoxin-Pn1a	563	3.45E-43	49.85%
13	comp24_c0	158034	*Ixodes scapularis*	myosin heavy chain	1277	2.50E-46	89.85%
14	comp120_c0	156078	*Latrodectus hesperus*	hypothetical protein [Latrodectus hesperus]	1133	1.85E-127	89.00%
15	comp109_c0	144127	*Bombus terrestris*	myosin heavy muscle isoform 1	5069	0.00E+00	85.30%

Continued ->

Table S8. (Continued):

#	*T. californicum* component	No. of mapped reads	Top BLASTX hit species	Sequence Description	Sequence Length (bp)	Top BLASTX hit E-vlaue	Mean similarity to top BLASTX hit
16	comp114_c0	138566	*Neoscona nautica*	tropomyosin	1367	9.21E-127	93.45%
17	comp22_c0	134695	*Haemaphysalis longicornis*	hemelipoglycoprotein	4669	0.00E+00	42.60%
18	comp130_c0	125013	*Latrodectus hesperus*	nidogen 1	258	1.20E-11	62.83%
19	comp41_c0	123819	*Phoneutria nigriventer*	predicted protein	436	1.06E-17	49.20%
20	comp228_c0	123454	*Latrodectus hesperus*	kunitz-like protease inhibitor precursor	1245	7.12E-99	54.05%
21	comp175_c0	121842	*Argiope bruennichi*	cytochrome c oxidase subunit i	1577	0.00E+00	87.10%
22	comp80_c0	102130	*Latrodectus hesperus*	hatching enzyme	351	5.53E-22	62.45%
23	comp220_c0	90300	*Latrodectus hesperus*	muscle lim protein mlp84b	1261	1.14E-43	83.30%
24	comp59_c0	87102	*Latrodectus hesperus*	adp atp translocase 2	622	2.01E-91	93.35%
25	comp16_c0	86297	*Lycosa singoriensis*	myosin light chain alkali	856	1.05E-67	72.70%
26	comp63_c0	86096	*Gallus gallus*	elongation factor 2	2751	0.00E+00	90.95%
27	comp418_c0	85926	*Amblyomma maculatum*	elongation factor 1 alpha	1884	0.00E+00	92.60%
28	comp82_c0	82337	*Haplopelma schmidti*	fibronectin type iii domain protein	355	1.15E-11	54.30%
29	comp275_c0	81273	*Amblyomma maculatum*	elongation factor 1-alpha	1635	0.00E+00	92.85%
30	comp214_c0	80672	*Latrodectus hesperus*	cg10527-like methyltransferase	634	7.98E-75	76.30%
31	comp604_c0	79214	*Camponotus floridanus*	polyadenylate-binding protein 1-like isoform 1	2776	0.00E+00	76.70%

Continued ->

Table S8. (Continued):

#	*T. californicum* component	No. of mapped reads	Top BLASTX hit species	Sequence Description	Sequence Length (bp)	Top BLASTX hit E-vlaue	Mean similarity to top BLASTX hit
32	comp67_c0	78403	*Latrodectus geometricus*	major ampullate spidroin 1 locus 2	577	2.07E-33	76.90%
33	comp124_c0	77688	*Limulus polyphemus*	arginine kinase	992	1.72E-147	89.45%
34	comp259_c0	76405	*Haliotis diversicolor supertexta*	cathepsin L-like	301	9.90E-16	90.90%
35	comp135_c0	74507	*Araneus ventricosus*	ferritin	667	2.48E-64	83.35%
36	comp374_c0	74430	*Ixodes scapularis*	calcium-transporting atpase sarcoplasmic endoplasmic reticulum type (calcium pump)	2896	0.00E+00	89.20%
37	comp185_c0	72861	*Culex quinquefasciatus*	s-adenosylmethionine synthetase	1428	1.36E-144	83.05%
38	comp170_c0	69661	*Tribolium castaneum*	hexokinase	1158	6.25E-86	70.40%
39	comp386_c0	67410	*Nephila clavata*	cytochrome oxidase subunit ii	755	3.21E-72	84.15%
40	comp136_c0	67174	*Haemaphysalis qinghaiensis*	troponin t	1363	1.51E-66	76.25%
41	comp291_c0	65218	*Araneus ventricosus*	secreted cystatin	394	1.68E-23	59.62%
42	comp488_c0	63922	*Lycosa singoriensis*	troponin i	1119	8.87E-58	74.10%
43	comp30_c0	62124	*Nasonia vitripennis*	proactivator polypeptide	3362	2.13E-118	47.20%
44	comp38_c0	61348	*Latrodectus hesperus*	astacin-like metalloprotease	526	5.7E-76	65.55%
45	comp43_c0	60753	*Ixodes scapularis*	heat shock protein 70	2025	0.00E+00	95.80%
46	comp108_c0	60129	*Argiope amoena*	major ampullate spidroin 1	359	1.07E-17	74.45%

Continued ->

Table S8. (Continued):

#	*T. californicum* component	No. of mapped reads	Top BLASTX hit species	Sequence Description	Sequence Length (bp)	Top BLASTX hit E-vlaue	Mean similarity to top BLASTX hit
47	comp165_c0	59514	*Lycosa singoriensis*	adp atp translocase	673	4.35E-48	90.45%
48	comp342_c0	58515	*Amblyomma maculatum*	muscular protein	992	1.93E-82	79.30%
49	comp404_c0	58024	*Rhipicephalus microplus*	paramyosin	3056	0.00E+00	83.90%
50	comp92_c0	57764	*Ixodes scapularis*	gtp-binding protein sar1b	2380	9.32E-94	92.15%
51	comp221_c0	57340	*Latrodectus hesperus*	lipid storage droplets surface-binding protein 2-like	535	1.05E-56	49.30%
52	comp329_c0	54886	*Drosophila virilis*	hydrolase	460	1.43E-06	52.10%
53	comp133_c0	54602	*Saccoglossus kowalevskii*	high-density lipoprotein-binding	2182	3.87E-131	70.05%
54	comp121_c0	54288	*Araneus ventricosus*	cystatin c	248	2.35E-25	63.95%
55	comp115_c0	53987	*Latrodectus hesperus*	ornithine decarboxylase antizyme	1181	2.70E-84	62.90%
56	comp277_c0	50211	*Sparus aurata*	cytoplasmic 2	411	1.34E-20	100.00%
57	comp93_c0	49078	*Dolomedes minor*	actin 5c	794	1.57E-144	99.00%
58	comp23_c0	48927	*Rhipicephalus microplus*	paramyosin	3146	0.00E+00	82.00%
59	comp484_c0	48501	*Dermacentor albipictus*	ferritin	881	3.52E-74	89.00%
60	comp454_c0	48341	*Penaeus monodon*	cathepsin d	1537	4.08E-150	78.60%
61	comp497_c0	46899	*Aphonopelma sp. SH-2011*	troponin c	1063	1.55E-64	84.00%

Continued ->

Table S8. (Continued):

#	*T. californicum* component	No. of mapped reads	Top BLASTX hit species	Sequence Description	Sequence Length (bp)	Top BLASTX hit E-vlaue	Mean similarity to top BLASTX hit
62	comp436_c0	46794	*Nephila inaurata madagascariensis*	hemocyanin subunit d	1955	0.00E+00	80.55%
63	comp88_c0	45487	*Nephila inaurata madagascariensis*	hemocyanin subunit 6	1919	0.00E+00	80.95%
64	comp337_c0	45450	*Danio rerio*	galectin family member (lec-1)	1294	2.75E-24	46.30%
65	comp154_c0	45289	*Latrodectus hesperus*	kunitz-like protease inhibitor precursor	799	4.49E-83	52.55%
66	comp905_c0	44725	*Tribolium castaneum*	alpha- sarcomeric-like isoform 1	4752	0.00E+00	90.05%
67	comp704_c0	44022	*Amblyomma maculatum*	d-3-phosphoglycerate dehydrogenase	1869	2.35E-142	65.80%
68	comp219_c0	43482	*Ixodes scapularis*	hypothetical protein IscW_ISCW009032 [Ixodes scapularis]	3656	2.39E-30	42.00%
69	comp290_c3	43140	*Ciona intestinalis*	apical endosomal glycoprotein	6047	0.00E+00	41.35%
70	comp216_c0	42641	*Noctiluca scintillans*	glucan -beta-glucosidase	586	1.39E-08	60.07%
71	comp467_c0	42407	*Tribolium castaneum*	high density lipoprotein binding protein	2722	0.00E+00	74.00%
72	comp367_c0	42085	*Latrodectus hesperus*	salivary plasminogen activator beta	2271	6.83E-62	56.10%
73	comp285_c0	41301	*Latrodectus hesperus*	fructose -bisphosphate aldolase	995	1.56E-156	85.25%
74	comp655_c0	40780	*Latrodectus hesperus*	histamine release factor	883	2.43E-83	80.65%
75	comp595_c0	39659	*Habronattus oregonensis*	cytochrome b	429	1.60E-29	77.35%
76	comp493_c0	38460	*Bos taurus*	tpa_exp: ubiquitin	600	2.60E-77	99.90%

Continued ->

Table S8. (Continued):

#	*T. californicum* component	No. of mapped reads	Top BLASTX hit species	Sequence Description	Sequence Length (bp)	Top BLASTX hit E-vlaue	Mean similarity to top BLASTX hit
77	comp129_c0	38317	*Litopenaeus vannamei*	zinc proteinase mpc1	270	6.74E-04	62.00%
78	comp590_c0	38134	*Latrodectus hesperus*	ribosomal protein sa	1083	9.84E-123	73.25%
79	comp417_c0	37855	*Latrodectus hesperus*	apolipophorin precursor protein	2060	5.38E-143	52.65%
80	comp925_c0	37808	*Blattella germanica*	enolase	2134	0.00E+00	87.25%
81	comp430_c0	36747	*Amblyomma maculatum*	actin 5c	574	8.67E-101	100.00%
82	comp471_c0	36704	*Latrodectus hesperus*	conserved plasmodium protein	1175	1.70E-46	56.80%
83	comp192_c0	36689	*Latrodectus hesperus*	hypothetical protein [Latrodectus hesperus]	536	2.53E-18	53.00%
84	comp226_c0	36634	*Latrodectus hesperus*	secreted salivary gland	749	8.16E-20	43.17%
85	comp734_c0	35955	*Argiope argentata*	elongation factor-1 gamma	1490	1.05E-179	78.60%
86	comp429_c0	35732	*Haemaphysalis longicornis*	vitellogenin	9386	1.02E-136	44.45%
87	comp69_c0	34799	*Ixodes scapularis*	cuticle	813	4.33E-20	66.85%
88	comp481_c0	34721	*Rhipicephalus microplus*	paramyosin	909	9.70E-14	65.30%
89	comp60_c0	33626	*Nephila clavipes*	aggregate spider glue 2	1197	3.01E-22	45.00%
90	comp773_c0	33622	*Nephila clavata*	cytochrome c oxidase subunit iii	744	1.37E-51	71.45%
91	comp579_c0	33053	*Latrodectus hesperus*	hypothetical protein [Latrodectus hesperus]	976	2.76E-33	59.75%

Continued ->

Table S8. (Continued):

#	*T. californicum* component	No. of mapped reads	Top BLASTX hit species	Sequence Description	Sequence Length (bp)	Top BLASTX hit E-vlaue	Mean similarity to top BLASTX hit
92	comp345_c0	32992	*Araneus ventricosus*	cathepsin b	808	5.25E-111	80.00%
93	comp311_c0	32571	*Amblyomma maculatum*	eukaryotic initiation factor 4a-ii	1933	0.00E+00	92.25%
94	comp538_c0	32513	*Latrodectus hesperus*	iclp2 protein	433	1.06E-17	57.94%
95	comp287_c0	32505	*Latrodectus hesperus*	serine protease	353	2.17E-10	84.00%
96	comp570_c0	32225	*Latrodectus hesperus*	ribosomal protein s2	1048	1.22E-122	91.55%
97	comp553_c0	32095	*Tribolium castaneum*	glutamine synthetase 2 (glutamate-amonia ligase)	2038	3.42E-166	84.45%
98	comp505_c0	31424	*Ixodes scapularis*	secreted salivary gland	982	3.63E-41	45.50%
99	comp561_c0	31108	*Latrodectus hesperus*	universal minicircle sequence binding protein	1334	2.84E-56	53.80%
100	comp91_c0	30527	*Xenopus laevis*	epididymal secretory protein e1 precursor	603	2.54E-32	63.90%

22) Table S9. Top 100 expressed components ("genes") in *T. grallator*

#	*T. grallator* component	No. of mapped reads	Top BLASTX hit species	Sequence Description	Sequence Length (bp)	Top BLASTX hit E-vlaue	Mean similarity to top BLASTX hit
1	comp158_c0	1305652	*Lycosa singoriensis*	actin	1599	0.00E+00	99.05%
2	comp195_c0	830801	*Latrodectus hesperus*	hypothetical protein [Latrodectus hesperus]	1142	6.22E-131	87.00%
3	comp619_c0	654764	*Latrodectus hesperus*	hexokinase isoform a	1776	9.24E-173	73.45%
4	comp758_c0	513150	*Ixodes scapularis*	heat shock protein 70	2313	0.00E+00	96.25%
5	comp761_c0	454687	*Argiope bruennichi*	cytochrome c oxidase subunit i	1576	0.00E+00	89.20%
6	comp358_c0	425513	*Hasarius adansoni*	opsin	1469	0.00E+00	80.05%
7	comp795_c0	420760	*Amblyomma maculatum*	elongation factor 1-alpha	1630	0.00E+00	93.50%
8	comp468_c0	415362	*Avicularia avicularia*	myosin regulatory light chain smooth muscle isoform	969	2.79E-70	75.40%
9	comp610_c0	350082	*Latrodectus hesperus*	cre-lea-1 protein	1912	1.77E-84	45.90%
10	comp444_c0	338354	*Suidasia medanensis*	blo t 13 allergen	756	2.41E-35	71.55%
11	comp737_c0	311112	*Ixodes scapularis*	salivary lipid interacting	832	1.61E-09	48.40%
12	comp628_c0	287429	*Neoscona nautica*	tropomyosin	1360	9.75E-129	93.55%
13	comp857_c0	278196	*Amblyomma americanum*	serine protease inhibitor serpin-4	1414	5.82E-31	47.00%
14	comp112_c0	262331	*Latrodectus hesperus*	secreted protein	312	1.84E-23	80.50%
15	comp162_c0	257454	*Brugia malayi*	sjchgc01393 protein	723	6.03E-17	71.70%

Continued ->

Table S9. (Continued):

#	*T. grallator* component	No. of mapped reads	Top BLASTX hit species	Sequence Description	Sequence Length (bp)	Top BLASTX hit E-vlaue	Mean similarity to top BLASTX hit
16	comp613_c0	255789	*Latrodectus hesperus*	adp atp translocase	1124	2.46E-140	90.90%
17	comp391_c0	254793	*Latrodectus hesperus*	ribosome associated membrane protein 4	766	2.11E-26	89.25%
18	comp900_c0	252383	*Ixodes scapularis*	glyceraldehyde 3-phosphate dehydrogenase	1322	4.05E-172	90.55%
19	comp898_c0	251052	*Haemaphysalis qinghaiensis*	troponin t	1953	1.35E-71	74.00%
20	comp774_c0	249912	*Latrodectus hesperus*	perilipin 2	1328	5.65E-81	44.70%
21	comp745_c0	243364	*Branchiostoma floridae*	pancreatic lipase-related protein 2	1373	3.38E-36	47.85%
22	comp353_c0	235880	*Latrodectus hesperus*	conserved plasmodium protein	786	4.58E-64	55.56%
23	comp567_c0	228101	*Latrodectus hesperus*	muscle lim protein isoform a	1068	3.06E-44	84.25%
24	comp714_c0	225756	*Latrodectus hesperus*	cofilin actin-depolymerizing factor homolog	1653	9.59E-76	76.90%
25	comp606_c0	222435	*Ixodes scapularis*	peritrophic membrane chitin binding	1615	2.33E-127	61.15%
26	comp902_c0	222426	*Acyrthosiphon pisum*	PREDICTED: hypothetical protein LOC100568673 [Acyrthosiphon pisum]	1606	2.63E-22	67.30%
27	comp810_c0	215614	*Ixodes scapularis*	tubulin alpha-1 chain	1569	0.00E+00	99.00%
28	comp1231_c0	215126	*Limulus polyphemus*	arginine kinase	1637	3.87E-170	90.15%
29	comp1082_c0	210537	*Amblyomma maculatum*	eukaryotic initiation factor 4a-ii	3287	0.00E+00	92.50%
30	comp845_c0	203539	*Ixodes scapularis*	heat shock protein 90	2452	0.00E+00	92.40%

Continued ->

Table S9. (Continued):

#	*T. grallator* component	No. of mapped reads	Top BLASTX hit species	Sequence Description	Sequence Length (bp)	Top BLASTX hit E-vlaue	Mean similarity to top BLASTX hit
31	comp675_c0	201669	*Amblyomma maculatum*	carboxypeptidase polypeptide 2	1177	1.23E-12	48.00%
32	comp634_c0	198433	*Latrodectus hesperus*	troponin i	1108	1.87E-68	77.05%
33	comp311_c0	197435	*Saccoglossus kowalevskii*	apolipoprotein d-like	974	1.56E-12	47.30%
34	comp890_c0	192907	*Tribolium castaneum*	ribosomal protein l3	1324	0.00E+00	92.65%
35	comp618_c0	192858	*Latrodectus hesperus*	hypothetical protein [Latrodectus hesperus]	954	1.61E-06	55.50%
36	comp968_c0	188813	*Latrodectus hesperus*	heat shock protein	1167	1.94E-10	54.20%
37	comp893_c0	180195	*Argiope argentata*	elongation factor-1 gamma	1499	0.00E+00	79.50%
38	comp426_c0	179434	*Pediculus humanus corporis*	abhydrolase domain-containing protein 11-like	1191	2.36E-27	47.05%
39	comp715_c0	178896	*Ixodes scapularis*	beta 2b	1653	0.00E+00	98.65%
40	comp1302_c0	175133	*Anopheles gambiae str. PEST*	enolase	1602	0.00E+00	87.00%
41	comp1823_c0	173429	*Tribolium castaneum*	myosin heavy isoform d	6551	0.00E+00	85.40%
42	comp404_c0	170130	*Lycosa singoriensis*	myosin light chain	711	2.32E-66	73.10%
43	comp1034_c0	169202	*Saccoglossus kowalevskii*	abhydrolase domain-containing protein 11-like	1110	8.27E-24	46.35%
44	comp776_c0	168782	*Aedes aegypti*	s-adenosylhomocysteine hydrolase	5745	0.00E+00	88.30%
45	comp477_c0	166592	*Nasonia vitripennis*	alpha beta hydrolase fold protein	1124	6.49E-16	43.65%

Continued ->

Table S9. (Continued):

#	*T. grallator* component	No. of mapped reads	Top BLASTX hit species	Sequence Description	Sequence Length (bp)	Top BLASTX hit E-vlaue	Mean similarity to top BLASTX hit
46	comp975_c0	165944	*Solenopsis invicta*	nucleosome assembly protein 1-like 1	1432	2.09E-76	69.45%
47	comp1129_c0	164138	*Ixodes scapularis*	eukaryotic translation initiation factor 3 subunit a	2712	3.70E-126	72.65%
48	comp710_c0	162168	*Lycosa singoriensis*	b-cell translocation gene anti-proliferative	1538	1.93E-83	68.80%
49	comp1058_c0	161351	*Bombus impatiens*	abhydrolase domain-containing protein 11-like	1010	2.17E-20	43.25%
50	comp1130_c0	160347	*Latrodectus hesperus*	ornithine decarboxylase antizyme	1247	1.60E-82	61.70%
51	comp598_c0	158676	*Bombus impatiens*	abhydrolase domain-containing protein 11-like	1074	3.45E-19	43.95%
52	comp512_c0	158650	*Oreochromis niloticus*	abhydrolase domain-containing protein 11-like	880	7.69E-21	45.60%
53	comp1471_c0	157490	*Danaus plexippus*	calcium-transporting atpase sarcoplasmic endoplasmic reticulum type (calcium pump)	4804	0.00E+00	89.30%
54	comp609_c0	157463	*Camponotus floridanus*	polyadenylate-binding protein 1-like isoform 1	2479	0.00E+00	75.95%
55	comp42_c0	157223	*Daphnia pulex*	s-adenosylmethionine synthetase	973	8.08E-102	82.75%
56	comp747_c0	156958	*Bombus impatiens*	abhydrolase domain containing 11	1030	3.09E-22	45.65%
57	comp817_c0	151528	*Tribolium castaneum*	muscleblind, isoform f	1493	6.56E-97	78.1%
58	comp1240_c0	151450	*Tribolium castaneum*	glutamine synthetase	1756	1.28E-158	85.20%
59	comp750_c0	149727	*Ornithodoros parkeri*	elongation factor 1-beta	800	7.73E-75	73.85%
60	comp386_c0	148834	*Amblyomma maculatum*	fibronectin type iii domain protein	675	3.52E-13	49.25%

Continued ->

Table S9. (Continued):

#	*T. grallator* component	No. of mapped reads	Top BLASTX hit species	Sequence Description	Sequence Length (bp)	Top BLASTX hit E-vlaue	Mean similarity to top BLASTX hit
61	comp836_c0	146861	*Ixodes scapularis*	Mid1-interacting protein	769	9.50E-51	47.60%
62	comp490_c0	145101	*Nasonia vitripennis*	saposin-related protein	3572	7.27E-125	47.50%
63	comp641_c0	144446	*Tribolium castaneum*	high density lipoprotein binding protein	4697	0.00E+00	72.85%
64	comp666_c0	143704	*Amblyomma maculatum*	actin 5c	667	9.58E-125	100.00%
65	comp1671_c0	143587	*Bombus impatiens*	cg14607 cg14607-pa	2127	6.83E-16	61.90%
66	comp771_c0	143403	*Nasonia vitripennis*	heat shock protein	1032	5.07E-57	74.65%
67	comp883_c0	142611	*Amblyomma variegatum*	fructose -bisphosphate aldolase	1700	1.39E-162	83.35%
68	comp1331_c0	141613	*Tribolium castaneum*	reticulon nogo	1546	1.04E-52	64.85%
69	comp436_c0	139325	*Ixodes scapularis*	nhl repeat-containing	3949	1.80E-140	73.15%
70	comp944_c0	138815	*Latrodectus hesperus*	translation initiation factor 5a	1047	2.10E-82	87.35%
71	comp642_c0	138736	*Lycosa singoriensis*	60s ribosomal protein l6	889	1.11E-91	71.75%
72	comp359_c0	136861	*Argiope trifasciata*	major ampullate spidroin-2	1366	1.20E-09	72.95%
73	comp1054_c0	132971	*Amblyomma maculatum*	galectin-9 isoform 2	1155	3.11E-45	46.85%
74	comp1478_c0	132523	*Daphnia pulex*	dnaj homolog subfamily a member 2	2522	6.18E-144	75.25%
75	comp1307_c0	132284	*Amblyomma maculatum*	transport protein sec61 subunit alpha 2	3194	0.00E+00	96.95%

Continued ->

Table S9. (Continued):

#	*T. grallator* component	No. of mapped reads	Top BLASTX hit species	Sequence Description	Sequence Length (bp)	Top BLASTX hit E-vlaue	Mean similarity to top BLASTX hit
76	comp1612_c0	132038	*Tribolium castaneum*	isoform a	1842	3.39E-109	62.80%
77	comp721_c0	131880	*Ixodes scapularis*	secreted protein, putative [Ixodes scapularis]	865	2.18E-04	51.00%
78	comp561_c0	130524	*Aphonopelma sp. SH-2011*	troponin c	952	5.82E-65	84.15%
79	comp1091_c0	130462	*Parasteatoda tepidariorum*	elongation factor 1 alpha	1111	1.21E-107	92.00%
80	comp799_c0	130072	*Ixodes scapularis*	cg9896-like protein	988	3.80E-38	46.45%
81	comp623_c0	129244	*Latrodectus hesperus*	histamine release factor	868	6.23E-84	80.60%
82	comp880_c0	129016	*Latrodectus hesperus*	ribosomal protein l19e	751	7.02E-80	94.30%
83	comp813_c0	128407	*Latrodectus hesperus*	type i restriction-modification system methyltransferase subunit	1114	2.49E-44	55.25%
84	comp834_c0	128097	*Latrodectus hesperus*	secreted salivary gland	756	2.20E-20	45.83%
85	comp535_c0	127173	*Latrodectus hesperus*	cg10527-like methyltransferase	633	7.98E-75	76.30%
86	comp1143_c0	124964	*Ixodes scapularis*	tubulin beta-2b chain	1586	0.00E+00	98.25%
87	comp1382_c0	124467	*Bombus terrestris*	myotubularin-related protein 14	1849	2.29E-73	55.30%
88	comp507_c0	124227	*Araneus ventricosus*	cathepsin b	2708	5.63E-159	77.45%
89	comp1176_c0	124153	*Drosophila mojavensis*	eukaryotic translation elongation factor	2758	0.00E+00	90.50%
90	comp1234_c0	123672	*Drosophila melanogaster*	calmodulin	1678	7.96E-78	99.70%

Continued ->

Table S9. (Continued):

#	*T. grallator* component	No. of mapped reads	Top BLASTX hit species	Sequence Description	Sequence Length (bp)	Top BLASTX hit E-vlaue	Mean similarity to top BLASTX hit
91	comp756_c0	123601	*Latrodectus hesperus*	tissue factor pathway inhibitor 2	868	7.81E-87	56.20%
92	comp848_c0	121743	*Latrodectus hesperus*	cellular nucleic acid-binding	1583	2.91E-74	60.25%
93	comp612_c0	121244	*Ornithodoros moubata*	ferritin	850	9.68E-74	88.10%
94	comp365_c0	120417	*Latrodectus hesperus*	ribosomal protein l5	1038	1.16E-141	88.30%
95	comp1161_c0	120136	*Culex quinquefasciatus*	predicted protein CpipJ_CPIJ006504	1290	1.66E-05	47.00%
96	comp1460_c0	119930	*Latrodectus hesperus*	14-3-3 zeta	1863	6.40E-132	96.75%
97	comp777_c0	119247	*Latrodectus hesperus*	60s ribosomal protein l10a	772	3.83E-100	91.75%
98	comp1484_c0	118703	*Ixodes scapularis*	pancreatic triacylglycerol lipase	1683	2.48E-95	55.90%
99	comp662_c0	118611	*Lycosa singoriensis*	60s ribosomal protein l10	806	6.78E-119	89.35%
100	comp1425_c0	118187	*Ixodes scapularis*	ornithine decarboxylase	2283	1.08E-123	67.75%

23) Table S10. Differentially expressed components ("genes", *n*=45) in *T. californicum* Color relative to Yellow

#	*T. californicum* Component ("Gene")	log Fold Change	*P*-value	FDR	Top BLASTX Species Hit	Sequence Description	Length (bp)	E-value of Top Hit	Mean Similarity
1	comp2787_c0	-11.40	1.54E-07	1.22E-03	*Ajellomyces capsulatus H88*	protein tar1-like	363	2.28E-10	63.25%
2	comp332_c0	7.12	1.81E-07	1.22E-03	*Limulus polyphemus*	astacin-like metalloprotease	957	3.13E-42	56.30%
3	comp12282_c0	-10.91	5.88E-07	2.08E-03	*Branchiostoma floridae*	voltage gated chloride channel domain-containing protein	1798	3.74E-12	43.10%
4	comp2123_c0	6.77	6.15E-07	2.08E-03	*Araneus diadematus*	cuticular	911	2.08E-24	64.85%
5	comp10591_c0	-10.54	1.60E-06	4.35E-03	*Pediculus humanus corporis*	Univin precursor, putative [Pediculus humanus corporis]	851	3.63E-04	47.00%
6	comp8118_c0	-10.36	2.59E-06	5.45E-03	*Saccoglossus kowalevskii*	ecm 18	294	5.19E-12	59.55%
7	comp8452_c0	-10.33	2.82E-06	5.45E-03	*Bombus impatiens*	u8-agatoxin-ao1a-like isoform 2	378	1.55E-08	73.61%
8	comp11645_c0	-10.21	3.97E-06	6.72E-03	*Mustela putorius furo*	heat-responsive protein 12	540	2.48E-37	72.75%
9	comp2982_c0	-7.18	4.56E-06	6.86E-03	*Ruminococcus torques ATCC 27756*	hypothetical protein RUMTOR_02906	151	1.25E-07	77.50%
10	comp6635_c0	-10.01	6.69E-06	8.98E-03	*Oikopleura dioica*	pg1 homology to homo sapiens	274	8.75E-12	63.38%
11	comp14130_c0	-9.98	7.29E-06	8.98E-03	*Trichinella spiralis*	hypothetical protein Tsp_11176 [Trichinella spiralis]	709	5.09E-05	41.00%
12	comp1476_c0	-9.94	7.97E-06	8.99E-03	*Ixodes scapularis*	cuticle	154	3.98E-12	74.65%
13	comp2143_c0	-6.45	1.15E-05	1.12E-02	*Nematostella vectensis*	protein	212	1.87E-14	80.05%
14	comp8616_c0	5.68	1.15E-05	1.12E-02	*Ixodes scapularis*	chitinase 1 precursor	2528	0	63.90%
15	comp8737_c0	-6.39	1.34E-05	1.19E-02	*Ixodes scapularis*	cuticle	163	2.83E-10	64.85%
16	comp991_c0	-5.57	1.41E-05	1.19E-02	*Ixodes scapularis*	cuticle	949	8.41E-24	59.30%

Continued ->

Table S10. (Continued):

#	*T. californicum* Component ("Gene")	log Fold Change	*P*-value	FDR	Top BLASTX Species Hit	Sequence Description	Length (bp)	E-value of Top Hit	Mean Similarity
17	comp69_c0	-5.48	1.55E-05	1.23E-02	*Ixodes scapularis*	cuticle	813	4.33E-20	66.85%
18	comp21700_c0	-9.57	2.13E-05	1.61E-02	*Platynereis dumerilii*	tubulin alpha-1 chain	1322	0	98.95%
19	comp9639_c0	-9.53	2.33E-05	1.66E-02	*Equus caballus*	e-selectin precursor	755	3.75E-04	40.75%
20	comp2053_c0	5.38	2.47E-05	1.67E-02	*Thermobia domestica*	hypothetical protein [Thermobia domestica]	1335	1.89E-07	79.00%
21	comp15161_c0	6.51	2.87E-05	1.85E-02	*Homo sapiens*	ALLW1950	266	2.10E-13	93.0%
22	comp6270_c0	6.38	3.39E-05	2.05E-02	---NA---	---NA---	207	---NA---	---NA---
23	comp27_c0	5.16	3.47E-05	2.05E-02	*Haemaphysalis longicornis*	hemelipoglycoprotein precursor	263	1.12E-06	60.78%
24	comp6139_c0	-5.36	4.44E-05	2.51E-02	*Micromonas sp. RCC299*	heme-binding protein 2	1341	9.62E-20	49.50%
25	comp8510_c0	-9.25	4.86E-05	2.53E-02	*Piriformospora indica*	stress response rci peptide	359	8.18E-10	79.10%
26	comp1821_c0	8.74	5.08E-05	2.53E-02	*Schistosoma mansoni*	rrna promoter binding protein	143	5.07E-04	61.50%
27	comp4209_c0	8.74	5.08E-05	2.53E-02	*Pediculus humanus corporis*	cytochrome p450-like tbp	295	2.04E-08	60.00%
28	comp83_c0	5.00	5.36E-05	2.53E-02	*Saccoglossus kowalevskii*	vitellogenin	6256	5.41E-102	42.75%
29	comp22837_c0	-9.19	5.61E-05	2.53E-02	*Ixodes scapularis*	serpin b3-like	1292	6.88E-68	57.90%
30	comp3137_c0	6.26	5.61E-05	2.53E-02	*Camponotus floridanus*	hypothetical protein EAG_03557 [Camponotus floridanus]	154	1.33E-07	81.40%
31	comp9329_c0	-5.83	6.04E-05	2.64E-02	*Clonorchis sinensis*	cathepsin l	1197	1.86E-64	54.95%
32	comp7875_c0	6.90	6.32E-05	2.68E-02	*Tribolium castaneum*	senescence-associated protein	396	1.25E-10	72.63%

Continued ->

Table S10. (Continued):

#	*T. californicum* Component ("Gene")	log Fold Change	*P*-value	FDR	Top BLASTX Species Hit	Sequence Description	Length (bp)	E-value of Top Hit	Mean Similarity
33	comp1352_c0	5.82	6.75E-05	2.69E-02	*Homo sapiens*	spindle pole body component 25 homolog (cerevisiae) isoform cra_a	157	1.12E-14	86.50%
34	comp1037_c0	5.10	6.90E-05	2.69E-02	*Cricetulus griseus-*	hypothetical protein	196	9.80E-11	69.00%
35	comp1848_c0	5.06	6.96E-05	2.69E-02	*Drosophila melanogaster*	LD48059p	328	2.20E-18	71.70%
36	comp1139_c0	4.87	7.69E-05	2.84E-02	*Ornithodoros moubata*	vitellogenin structural genes (yolk protein genes) family member (vit-1)-like	4774	4.30E-27	39.75%
37	comp3431_c0	5.09	7.76E-05	2.84E-02	*Homo sapiens*	p150	890	1.09E-163	98.15%
38	comp15352_c0	-9.03	8.55E-05	3.05E-02	*Latrodectus hesperus*	serine proteinase inhibitor	1064	7.55E-19	41.70%
39	comp16320_c0	-8.93	1.10E-04	3.79E-02	*Trichinella spiralis*	nematode cuticle collagen n-terminal domain containing protein	404	6.11E-05	57.63%
40	comp253_c0	5.27	1.13E-04	3.79E-02	*Macaca fascicularis*	zinc finger protein isoform cra_a	329	1.60E-32	86.50%
41	comp22124_c0	-8.92	1.15E-04	3.79E-02	*Nematostella vectensis*	predicted protein [Nematostella vectensis]	128	3.94E-04	73.00%
42	comp19046_c0	4.98	1.48E-04	4.79E-02	*Latrodectus hesperus*	egg case silk protein 2	627	3.40E-46	72.33%
43	comp7727_c0	-8.79	1.57E-04	4.96E-02	*Ixodes scapularis*	cuticle	321	3.61E-05	63.75%
44	comp19895_c0	-8.77	1.65E-04	4.97E-02	*Acyrthosiphon pisum*	clotting factor b-like	675	2.49E-19	51.80%
45	comp5933_c1	-8.77	1.65E-04	4.97E-02	*uncultured nuHF2 cluster bacterium HF0130_29D04*	hypothetical protein	151	1.98E-11	76.90%

The reported top BLASTX species hits and transcript lengths are from the transcripts (isoforms) with smallest BLASTX E-value. NB although the top hit reported here is sometimes microbial (e.g. 1, 9, 45) this does not mean that all the BLAST hits were microbial and Megan accounts for this in assigning taxa to sequences. NA = No BLAST hit detected.

24) Table S11. Differentially expressed components (“genes”, Top 100) in *T. grallator* Color relative to Yellow

#	*T. grallator* Component (“Gene”)	log Fold Change	*P*-value	FDR	Top BLASTX Species Hit	Sequence Description	Length (bp)	E-value of Top Hit	Mean Similarity
1	comp2165_c0	-13.36	6.48E-48	7.94E-44	*Blattabacterium sp. (Mastotermes darwiniensis) str. MADAR*	hypothetical protein MADAR_309 [Blattabacterium sp. (Mastotermes darwiniensis) str. MADAR]	285	2.20E-10	57.81%
2	comp3587_c0	6.26	4.60E-35	2.81E-31	*Latrodectus hesperus*	astacin-like metalloprotease	935	6.06E-35	57.20%
3	comp4363_c0	5.63	2.94E-31	1.20E-27	*Tigriopus japonicus*	vitellogenin 2	1585	1.09E-20	43.20%
4	comp16337_c0	-10.91	2.54E-28	7.78E-25	*Drosophila melanogaster*	IP15837p [Drosophila melanogaster]	1273	9.25E-09	52.00%
5	comp16006_c0	5.89	1.16E-25	2.84E-22	*Culex quinquefasciatus*	scavenger receptor cysteine-rich protein	1950	1.84E-44	41.85%
6	comp138_c0	-10.27	1.77E-23	3.62E-20	*Daphnia pulex*	hypothetical protein DAPPUDRAFT_118838	260	1.60E-16	70.95%
7	comp15863_c0	7.63	3.33E-23	5.82E-20	*Latrodectus hesperus*	leucine-rich transmembrane	1236	3.59E-18	47.30%
8	comp18968_c0	10.25	2.92E-19	4.47E-16	*Latrodectus hesperus*	hypothetical protein [Latrodectus hesperus]	313	3.61E-26	71.50%
9	comp10123_c0	4.05	4.54E-18	6.17E-15	*Ixodes scapularis*	transmembrane protein 20	1944	2.72E-48	59.45%
10	comp15668_c0	-5.16	2.03E-17	2.48E-14	*Amblyomma variegatum*	pra1 family protein 3	1239	4.86E-07	60.20%
11	comp2679_c0	3.84	2.30E-17	2.56E-14	*Ixodes scapularis*	novel protein containing von willebrand factor type c domain	466	3.48E-08	44.80%
12	comp3416_c0	-3.83	2.67E-17	2.70E-14	*Tribolium castaneum*	cytochrome p450 cyp18a1	516	6.66E-15	53.75%
13	comp17074_c0	-9.40	2.86E-17	2.70E-14	*Ixodes scapularis*	lipase precursor, putative [Ixodes scapularis]	667	6.36E-04	49.00%
14	comp4476_c0	-5.79	1.59E-16	1.39E-13	*Branchiostoma floridae*	hypothetical protein BRAFLDRAFT_126840 [Branchiostoma floridae]	168	3.01E-04	50.00%

Continued ->

Table S11. (Continued):

#	*T. grallator* Component ("Gene")	log Fold Change	*P*-value	FDR	Top BLASTX Species Hit	Sequence Description	Length (bp)	E-value of Top Hit	Mean Similarity
15	comp3870_c0	3.55	3.46E-16	2.83E-13	*Haemaphysalis longicornis*	vitellogenin structural genes (yolk protein genes) family member (vit-1)-like	412	1.75E-20	44.85%
16	comp12836_c0	-4.49	6.29E-16	4.82E-13	*Ixodes scapularis*	myosin light chain 2	729	1.63E-17	65.05%
17	comp18438_c0	3.65	8.99E-16	6.48E-13	*Haemaphysalis longicornis*	vitellogenin fused with superoxide dismutase	1718	1.35E-80	44.15%
18	comp33469_c0	9.52	2.64E-15	1.80E-12	*Daphnia pulex*	speckle-type poz protein	1500	2.32E-17	51.65%
19	comp1253_c0	3.18	9.89E-14	6.38E-11	*Ixodes scapularis*	matrix metalloproteinase 1 isoform 1	1274	4.67E-69	61.30%
20	comp28046_c0	5.44	1.15E-13	7.06E-11	*Heterocephalus glaber*	tissue factor pathway inhibitor 2	1782	1.55E-10	60.40%
21	comp60670_c0	9.18	2.75E-13	1.60E-10	*Haemaphysalis longicornis*	Vitellogenin-B [Haemaphysalis longicornis]	1102	2.10E-11	43.50%
22	comp6481_c0	5.99	4.73E-13	2.63E-10	*Branchiostoma floridae*	cd93 molecule	279	1.81E-09	52.30%
23	comp3284_c0	-3.09	5.28E-13	2.81E-10	*Strongylocentrotus purpuratus*	cytochrome family subfamily polypeptide 6	1246	1.12E-43	51.70%
24	comp9866_c0	3.80	6.37E-13	3.25E-10	*Hydra magnipapillata*	meprin a subunit beta	573	6.34E-27	60.05%
25	comp28353_c0	4.62	7.76E-13	3.80E-10	*Branchiostoma floridae*	zinc finger protein 474-like	901	3.36E-35	78.90%
26	comp50755_c0	9.04	1.66E-12	7.80E-10	*Bombus terrestris*	glucose dehydrogenase	1530	2.65E-109	60.25%
27	comp10_c1	-2.98	2.41E-12	1.09E-09	*Daphnia pulex*	rrna intron-encoded homing endonuclease	134	1.06E-09	75.65%
28	comp11546_c0	-3.15	4.08E-12	1.78E-09	*Latrodectus hesperus*	serine protease	1588	1.07E-84	50.60%
29	comp8002_c0	8.82	5.50E-12	2.32E-09	---NA---	---NA---	166	---NA---	---NA---

Continued ->

Table S11. (Continued):

#	*T. grallator* Component ("Gene")	log Fold Change	*P*-value	FDR	Top BLASTX Species Hit	Sequence Description	Length (bp)	E-value of Top Hit	Mean Similarity
30	comp16536_c0	-3.33	5.82E-12	2.38E-09	*Daphnia pulex*	spaetzle-like protein	808	1.86E-07	42.75%
31	comp15505_c0	3.63	6.55E-12	2.59E-09	*Xenopus laevis*	leucine-rich repeat-containing protein 67-like	912	1.35E-23	57.75%
32	comp4235_c0	-2.95	8.57E-12	3.28E-09	*Ixodes scapularis*	cuticular	763	4.67E-26	63.10%
33	comp28806_c0	8.77	9.79E-12	3.63E-09	*Methylomicrobium album BG8*	transposase	708	1.01E-55	60.95%
34	comp17324_c0	-3.48	1.03E-11	3.63E-09	*Nasonia vitripennis*	cytosolic carboxypeptidase 6	1076	5.16E-15	57.75%
35	comp16901_c0	4.24	1.04E-11	3.63E-09	*Latrodectus hesperus*	cysteine-rich venom	1274	6.00E-08	51.80%
36	comp41276_c0	-8.48	1.60E-11	5.44E-09	*Plasmodium falciparum 3D7*	ribosomal rna	1368	2.78E-06	42.47%
37	comp42621_c0	8.71	2.15E-11	7.13E-09	*Ixodes scapularis*	homeobox protein goosecoid isoform a-like	1423	1.72E-22	78.20%
38	comp19325_c0	-3.45	4.74E-11	1.53E-08	*Latrodectus hesperus*	astacin-like metalloprotease	1078	3.20E-73	59.35%
39	comp161_c0	-2.92	5.41E-11	1.70E-08	*Latrodectus hesperus*	rrna promoter binding protein	335	1.86E-38	80.42%
40	comp18097_c0	-3.46	5.77E-11	1.77E-08	*Cricetulus griseus*	sporulation meiosis- spo11 homolog (cerevisiae)	869	2.73E-55	71.80%
41	comp15263_c0	-3.27	6.27E-11	1.87E-08	*Caenorhabditis brenneri*	protein nas-13	1140	3.74E-27	54.35%
42	comp8293_c0	2.81	1.22E-10	3.56E-08	*Xenopus laevis*	methenyltetrahydrofolate synthetase domain containing	3867	1.70E-48	65.25%
43	comp13491_c0	3.09	2.04E-10	5.72E-08	*Ixodes scapularis*	hypothetical protein	1588	9.76E-62	47.15%
44	comp35166_c0	-8.28	2.05E-10	5.72E-08	*Trichomonas vaginalis G3*	ankyrin repeat protein	1714	2.60E-07	55.25%
45	comp21607_c0	-2.99	3.12E-10	8.50E-08	*Bombus impatiens*	tbc1 domain family member 9	1548	3.17E-134	66.55%

Continued ->

Table S11. (Continued):

#	*T. grallator* Component ("Gene")	log Fold Change	*P*-value	FDR	Top BLASTX Species Hit	Sequence Description	Length (bp)	E-value of Top Hit	Mean Similarity
46	comp51695_c0	8.46	4.10E-10	1.09E-07	*Strongylocentrotus purpuratus*	ankyrin unc44	443	1.51E-27	59.55%
47	comp36788_c0	-2.88	5.60E-10	1.46E-07	*Ixodes scapularis*	cub domain low-density lipoprotein receptor domain class	2357	1.28E-103	45.90%
48	comp9434_c1	2.71	5.83E-10	1.49E-07	*Amblyomma maculatum*	acid phosphatase-1	1180	3.11E-56	54.25%
49	comp8118_c0	2.70	6.18E-10	1.52E-07	*Drosophila melanogaster*	IP15837p [Drosophila melanogaster]	751	1.95E-05	47.00%
50	comp4453_c0	-2.67	6.20E-10	1.52E-07	*Latrodectus hesperus*	nidogen 1	398	7.24E-22	60.35%
51	comp18480_c0	2.85	7.45E-10	1.79E-07	*Ixodes scapularis*	chorion peroxidase	2541	1.60E-123	53.55%
52	comp24209_c0	-2.98	7.83E-10	1.84E-07	*Hydra magnipapillata*	protein	230	2.25E-07	69.85%
53	comp1881_c0	2.60	8.19E-10	1.89E-07	*Latrodectus hesperus*	kunitz-like protease inhibitor precursor	416	2.61E-08	69.05%
54	comp16930_c0	-2.69	1.16E-09	2.63E-07	*Ixodes scapularis*	2 -cyclic-nucleotide 3 -phosphodiesterase	2765	5.99E-31	45.55%
55	comp37980_c0	-4.56	1.30E-09	2.90E-07	*Daphnia pulex*	pancreatic triacylglycerol lipase	827	5.10E-16	56.70%
56	comp8757_c0	2.59	1.35E-09	2.95E-07	*Daphnia pulex*	kynureninase (l-kynurenine hydrolase)	2233	1.91E-117	62.25%
57	comp45067_c0	-3.52	1.46E-09	3.13E-07	*Acyrthosiphon pisum*	glutamate-gated chloride channel	1620	6.84E-111	59.65%
58	comp6312_c0	2.54	1.61E-09	3.35E-07	*Branchiostoma floridae*	cg3556 cg3556-pa	1960	4.13E-28	43.10%
59	comp3401_c0	2.54	1.62E-09	3.35E-07	*Nasonia vitripennis*	trans- -dihydrobenzene- -diol dehydrogenase	1234	3.16E-83	64.05%
60	comp9665_c0	2.63	1.75E-09	3.56E-07	*Mus musculus*	speckle-type poz protein	1521	5.26E-33	47.50%
61	comp45274_c0	8.31	2.03E-09	4.07E-07	*Tetraodon nigroviridis*	extracellular matrix protein fras1	1248	2.71E-05	61.40%

Continued ->

Table S11. (Continued):

#	*T. grallator* Component ("Gene")	log Fold Change	*P*-value	FDR	Top BLASTX Species Hit	Sequence Description	Length (bp)	E-value of Top Hit	Mean Similarity
62	comp4563_c0	2.52	3.38E-09	6.67E-07	*Schistocerca gregaria*	heat shock protein	654	2.44E-29	62.35%
63	comp11204_c0	3.05	3.48E-09	6.77E-07	*Latrodectus hesperus*	hatching enzyme	776	6.97E-25	62.00%
64	comp8387_c0	2.48	3.60E-09	6.89E-07	*Danaus plexippus*	ldl receptor ligand-binding repeat bearing	2511	1.24E-19	47.55%
65	comp25463_c0	3.77	3.95E-09	7.37E-07	*Ixodes scapularis*	gustatory receptor 64f-like	1201	1.96E-05	44.60%
66	comp18622_c0	3.28	3.97E-09	7.37E-07	*Tribolium castaneum*	transmembrane and tpr repeat-containing protein cg4050-like	1091	3.07E-39	63.95%
67	comp11194_c0	2.50	4.53E-09	8.29E-07	*Anopheles gambiae str. PEST*	sodium-bile acid cotransporter	2899	6.94E-62	57.65%
68	comp3713_c0	2.47	4.76E-09	8.58E-07	*Amblyomma maculatum*	secreted salivary gland	871	4.72E-07	41.33%
69	comp21946_c0	-2.67	5.27E-09	9.36E-07	*Ixodes scapularis*	transmembrane protein 184b	2144	5.83E-132	79.25%
70	comp2545_c0	2.47	5.93E-09	1.04E-06	*Rattus norvegicus*	grn2 protein	709	5.08E-13	51.45%
71	comp31840_c0	-6.11	6.71E-09	1.14E-06	*Clonorchis sinensis*	uncharacterized transposase-like protein	1188	8.09E-12	58.70%
72	comp29627_c0	-4.77	6.71E-09	1.14E-06	*Ixodes scapularis*	isoform a	624	1.05E-15	67.80%
73	comp13729_c0	-2.97	7.01E-09	1.18E-06	*Ixodes scapularis*	proteasome activator complex subunit 4	1463	7.20E-165	71.40%
74	comp12938_c0	8.18	8.62E-09	1.43E-06	*Pediculus humanus corporis*	cytochrome c	105	3.41E-11	92.58%
75	comp6668_c0	-2.41	8.88E-09	1.45E-06	*Ixodes scapularis*	proteasome activator complex subunit 4-like	2454	0	60.40%
76	comp4870_c0	-2.41	1.06E-08	1.71E-06	*Araneus diadematus*	cuticular protein 100a	1038	2.49E-11	59.80%
77	comp267_c0	-2.35	1.18E-08	1.88E-06	*Rattus norvegicus*	zgc:158463 protein	497	6.25E-31	68.70%

Continued ->

Table S11. (Continued):

#	*T. grallator* Component ("Gene")	log Fold Change	*P*-value	FDR	Top BLASTX Species Hit	Sequence Description	Length (bp)	E-value of Top Hit	Mean Similarity
78	comp12409_c0	3.53	1.21E-08	1.91E-06	*Nematostella vectensis*	upf0740 protein c1orf192 homolog	541	1.98E-10	52.10%
79	comp986_c0	-2.34	1.39E-08	2.15E-06	*Caerostris extrusa*	txca_caeex ame: full=u3-aranetoxin-ce1a short=u3-aatx-ce1a ame: full=neurotoxic peptide caeron flags: precursor	601	3.46E-05	49.57%
80	comp43282_c0	3.77	1.44E-08	2.20E-06	*Daphnia pulex*	nicotinic acetylcholine receptor subunit a5	1619	1.19E-147	70.45%
81	comp8296_c0	-2.49	1.60E-08	2.42E-06	*Mesocricetus auratus*	serum amyloid a	822	1.90E-39	72.80%
82	comp38452_c0	3.57	1.83E-08	2.73E-06	*Tribolium castaneum*	neural cell adhesion molecule	1798	4.55E-26	61.50%
83	comp8951_c0	2.38	2.18E-08	3.22E-06	*Ixodes scapularis*	n-acetylneuraminate pyruvate lyase	1147	5.05E-64	60.75%
84	comp44467_c0	8.09	2.36E-08	3.44E-06	*Ixodes scapularis*	hypothetical protein IscW_ISCW013795 [Ixodes scapularis]	1301	2.70E-112	56.88%
85	comp20368_c0	-5.99	2.69E-08	3.84E-06	*Ernolatia moorei*	tyrosine hydroxylase	454	1.31E-23	92.90%
86	comp12109_c0	2.40	2.69E-08	3.84E-06	*Latrodectus hesperus*	monocarboxylate	2355	1.55E-16	49.60%
87	comp6412_c0	2.46	2.89E-08	4.02E-06	*Latrodectus hesperus*	hypothetical protein [Latrodectus hesperus]	922	1.10E-04	42.00%
88	comp21857_c0	-2.61	2.89E-08	4.02E-06	*Anopheles darlingi*	substance p receptor (long form)	231	1.56E-08	60.15%
89	comp9931_c0	-2.41	2.95E-08	4.06E-06	*Cricetulus griseus*	fanconi anemia group a protein	1515	3.28E-27	45.20%
90	comp3771_c0	2.30	3.43E-08	4.67E-06	*Mus musculus*	carbonic anhydrase 1	1117	2.69E-30	47.40%
91	comp56885_c0	4.33	3.69E-08	4.97E-06	*Culex quinquefasciatus*	cytochrome p450	984	3.08E-56	57.85%

Continued ->

Table S11. (Continued):

#	*T. grallator* Component ("Gene")	log Fold Change	*P*-value	FDR	Top BLASTX Species Hit	Sequence Description	Length (bp)	E-value of Top Hit	Mean Similarity
92	comp18857_c0	-2.61	3.77E-08	5.01E-06	*Tribolium castaneum*	bel12_ag transposon polyprotein	3031	1.63E-163	57.25%
93	comp5965_c0	2.29	4.09E-08	5.39E-06	*Tribolium castaneum*	speckle-type poz	2034	9.98E-25	54.30%
94	comp5866_c0	-2.27	4.16E-08	5.42E-06	*Ixodes scapularis*	ankyrin repeat domain-containing protein 17	5171	9.38E-153	68.50%
95	comp15912_c0	3.04	4.31E-08	5.56E-06	*Acyrthosiphon pisum*	PREDICTED: hypothetical protein LOC100569550 [Acyrthosiphon pisum]	352	8.83E-04	51.00%
96	comp34013_c0	3.15	4.53E-08	5.78E-06	*Latrodectus hesperus*	antigen 5 scp domain-containing	1401	6.49E-75	52.05%
97	comp13968_c0	2.74	5.15E-08	6.50E-06	*Latrodectus hesperus*	hypothetical protein [Latrodectus hesperus]	533	3.03E-08	60.00%
98	comp1782_c0	2.23	6.27E-08	7.84E-06	*Schistocerca gregaria*	nicotinic acetylcholine receptor subunit a2	1162	4.42E-31	52.30%
99	comp15911_c0	7.98	6.70E-08	8.29E-06	*Oikopleura dioica*	unnamed protein product [Oikopleura dioica]	402	5.19E-04	44.67%
100	comp4590_c0	-2.45	6.83E-08	8.37E-06	*Araneus diadematus*	cu26_aradi ame: full=adult-specific rigid cuticular protein short=acp	602	2.47E-19	72.33%

The reported top BLASTX species hits and transcript lengths are from the transcripts (isoforms) with smallest BLASTX E-value. NB although the top hit reported here is sometimes microbial (e.g. 1) this does not mean that all the BLAST hits were microbial and Megan accounts for this in assigning taxa to sequences. NA = No BLAST hit detected.

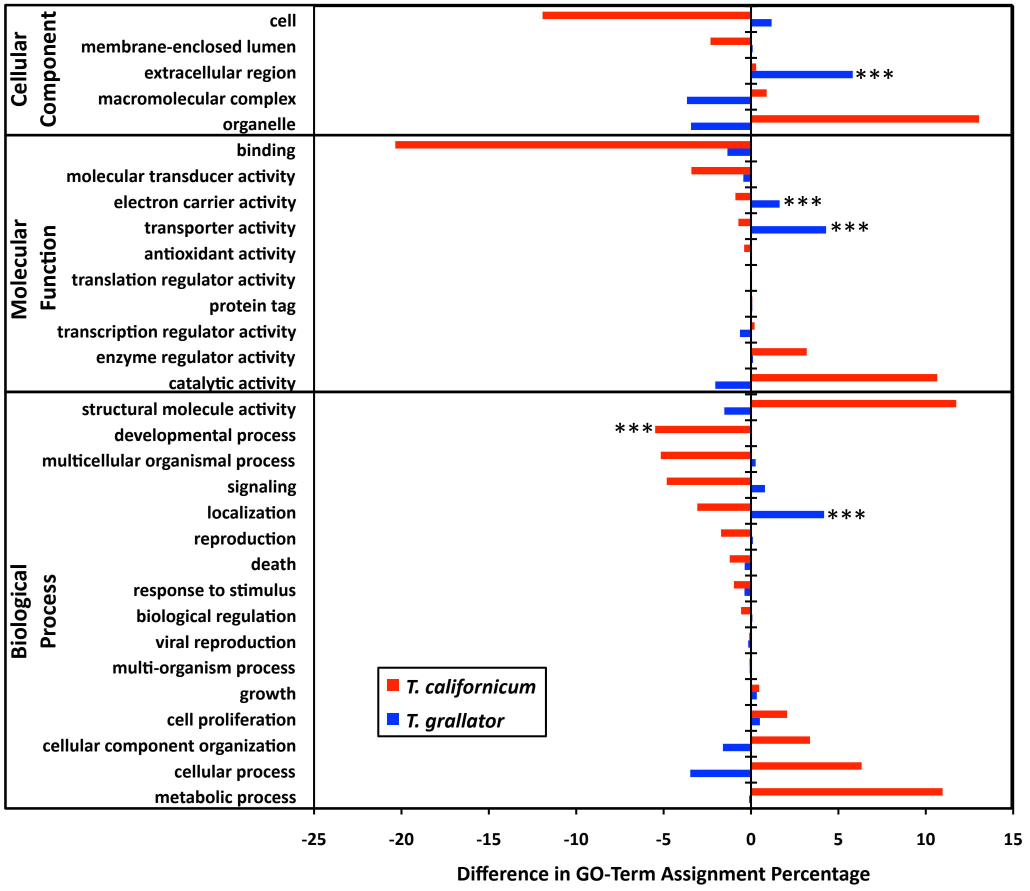

25) Figure S11. Differential expressed GO-terms in Color relative to Yellow. Values are expressed in terms of difference in GO-term assignment percentage between DE genes and total transcriptome. Significance based upon actual counts and after Bonferroni correction. Chart is divided by GO domains: cellular component, molecular function and biological process. Both level II and level III terms are shown.

26) *Text:* Differential expressed GO-terms in Color relative to Yellow

Fig. S11 above, examines the difference in GO-term assignment percentage between the entire transcriptome and the DE gene sets. GO-terms that are relatively enriched in the DE gene sets are shown as positive and those that are less common are shown as negative. Level II and level III terms are plotted together. The histogram suggests little agreement between the two species with respect to DE GO-term enrichment. Since very few components were suggested as DE in *T. californicum* the results for this species are probably mostly noise. Focusing on *T. grallator*, significant enrichment was seen for the cellular component term "extracellular region" (GO:0005576), the molecular functions "electron carrier activity" (GO:0009055) and "transporter activity" (GO:0005215), and the biological process "localization" (GO:0051179).

BLAST2GO enrichment analyses of the cellular component domain supported the term "extracellular space" (GO: 0005615; *T. grallator* FDR = 0.0003 – a child of GO:0005576) and gave suggestive support – significant before but not after FDR correction – for "external encapsulating structure" (GO:0030312), "cell envelope" (GO:0030313), and "lysosome" (GO:0005764). BLAST2GO enrichment analyses of molecular function supported the terms "transporter activity" (GO:0005215; *T. grallator* FDR = 0.0010), "electron carrier activity" (GO:0009055; *T. grallator* FDR = 0.0029) and gave suggestive support for oxygen binding (GO:0019825 – a child term of "binding" GO:0005488). BLAST2GO enrichment analyses of biological process supported a role for "transport" (GO:0006810; *T. grallator* FDR = 0.0029 – a child of GO:0051179) and gave suggestive support for "response to external stimulus" (GO:0050896).

All of these terms could well relate to processes involving the ommochrome and/or pteridin pigment pathways and pigment granule formation processes (see descriptions of the pathways above) and suggest that at least the *T. grallator* DE component set may contain additional pigmentation-associated genes and pathways that play a significant role in the color polymorphism.

27) Table S12. Venom genes identified by reciprocal best hit (RBH) blast against the Arachnoserver database.

	Venom Protein	Species	Homologous Component	%Identity	BLASTX E-value	RBH TBLASTN E-value
1.	alpha-latrotoxin-associated	*T. californicum:*	comp2862_c0	33	1e-07	3e-05
		T. grallator:	comp4833_c0	28	3e-07	4e-05
2.	alpha-latrotoxin-associated_LMWPlsp:P49125l288	*T. californicum:*	comp10788_c0	36	6e-09	2e-05
		T. grallator:	—	—	—	—
3.	alpha-latroinsectotoxin-Lt1alsp:Q02989l62	*T. californicum:*	comp30464_c0	24	3e-21	8e-16
		T. grallator:	comp32957_c0	20	2e-09	6e-19
4.	kappa-theraphotoxin-Hh1a_2lgb:ACD01237l326	*T. californicum:*	comp3301_c0	58	9e-16	2e-12
		T. grallator:	—	—	—	—
5.	kappa-theraphotoxin-Hh1blsp:B2ZBB6l458	*T. californicum:*	—	—	—	—
		T. grallator:	comp7457_c0	57	4e-15	9e-12
6.	U1-aranetoxin-Av1alsp:Q8T3S7l114	*T. californicum:*	comp782_c0	53	3e-17	1e-13
		T. grallator:	comp8591_c0	54	7e-17	1e-13
7.	U1-filistatoxin-Kh1bl1211	*T. californicum:*	comp85105_c0	48	1e-23	2e-13
		T. grallator:	—	—	—	—
8.	U1-hexatoxin-Iw1alsp:Q5D231l601	*T. californicum:*	comp463_c0	34	1e-08	9e-07
		T. grallator:	comp63460_c0	34	5e-09	8e-07
9.	U1-lycotoxin-Ls1b_12lsp:B6DCJ3l884	*T. californicum:*	—	—	—	—
		T. grallator:	comp4523_c0	39	4e-20	4e-16
10.	U3-aranetoxin-Ce1alsp:Q8MTX1l300	*T. californicum:*	comp575_c0	40	7e-16	2e-08
		T. grallator:	comp2640_c0	32	6e-10	5e-06
11.	U3-ctenitoxin-Asp1alsp:P84001l14	*T. californicum:*	comp464_c0	73	9e-20	9e-16
		T. grallator:	comp3070_c0	73	1e-20	4e-17
12.	U3-lycotoxin-Ls1a_8lsp:B6DCR4l938	*T. californicum:*	comp5452_c0	36	7e-12	9e-08
		T. grallator:	—	—	—	—
13.	U4-lycotoxin-Ls1a_3lsp:B6DCT8l966	*T. californicum:*	—	—	—	—
		T. grallator:	comp4523_c0	35	3e-13	2e-09
14.	U5-lycotoxin-Ls1alsp:B6DCV0l983	*T. californicum:*	comp5452_c0	33	2e-17	4e-14
		T. grallator:	—	—	—	—
15.	U3-lycotoxin-Ls1glsp:B6DCR6l949	*T. californicum:*	comp5452_c0	43	5e-13	3e-07
		T. grallator:	—	—	—	—
16.	U8-agatoxin-Ao1alsp:Q5Y4U4l72	*T. californicum:*	comp16089_c0	52	2e-12	3e-05
		T. grallator:	comp3750_c0	38	4e-10	2e-05
17.	U9-ctenitoxin-Pr1alsp:P83893l214	*T. californicum:*	comp51_c0	57	4e-23	1e-16
		T. grallator:	comp50890_c0	52	1e-22	4e-16

Continued ->

Table S12. (Continued):

	Venom Protein	Species	Homologous Component	%Identity	BLASTX E-value	RBH TBLASTN E-value
18.	U15-lycotoxin-Ls1a|sp:B6DD43|1066	*T. californicum:*	—	—	—	—
		T. grallator:	comp2544_c0	38	3e-15	2e-10
19.	U15-lycotoxin-Ls1f_2|sp:B6DD40|1071	*T. californicum:*	comp2324_c0	37	3e-09	3e-04
		T. grallator:	—	—	—	—
20.	U15-theraphotoxin-Hh1a|sp:B2ZBB8|481	*T. californicum:*	comp8918_c0	53	3e-11	3e-10
		T. grallator:	—	—	—	—
21.	U19-ctenitoxin-Pn1a|sp:P83997|213	*T. californicum:*	—	—	—	—
		T. grallator:	comp21747_c0	51	2e-14	1e-14
22.	U21-ctenitoxin-Pn1a|sp:P84033|11	*T. californicum:*	comp1531_c0	48	4e-05	3e-04
		T. grallator:	comp4181_c0	50	6e-06	6e-05
23.	U33-theraphotoxin-Cj1c|sp:B1P1J1|870	*T. californicum:*	comp1175_c0	33	8e-08	7e-05
		T. grallator:	—	—	—	—

28) Table S13. "House-Keeping" genes (n = 196) used for DE normalization and estimation of common dispersion

#	Gene Symbol	FlyBase gene	*T. californicum* component	Length (bp)	*T. grallator* component	Length (bp)	Best Reciprocal BLASTX hit[1]	E-value of best hit
1	*Alh*	FBgn0261238	comp21567_c0	1718	comp15241_c0	2205	gi|212515959|gb|EEB18024.1| protein AF-10, putative [*Pediculus humanus corporis*]	1.10×10^{-106}
2	*AP-2sigma*	FBgn0043012	comp4071_c0	644	comp3642_c0	1200	gi|215505504|gb|EEC14998.1| clathrin adaptor complex, small subunit [*Ixodes scapularis*]	9.92×10^{-76}
3	*Aprt*	FBgn0000109	comp4283_c0	1124	comp649_c0	872	gi|270010221|gb|EFA06669.1| hypothetical protein TcasGA2_TC009597 [*Tribolium castaneum*]	6.34×10^{-44}
4	*Arf72A*	FBgn0000115	comp10773_c0	2115	comp8407_c0	2106	gi|346470451|gb|AEO35070.1| hypothetical protein [*Amblyomma maculatum*]	2.13×10^{-86}
5	*Arf84F*	FBgn0004908	comp4760_c0	957	comp11656_c0	1233	gi|291237670|ref|XP_002738749.1| PREDICTED: ADP-ribosylation factor-like 2-like [*Saccoglossus kowalevskii*]	1.91×10^{-88}
6	*Arpc3A*	FBgn0038369	comp5123_c0	1141	comp2533_c0	1136	gi|108879910|gb|EAT44135.1| arp2/3 complex 21 kd subunit [*Aedes aegypti*]	8.79×10^{-77}
7	*ATPsyn-Cf6*	FBgn0016119	comp2535_c0	825	comp1544_c0	644	gi|161669198|gb|ABX75451.1| ATPase coupling factor 6 [*Lycosa singoriensis*]	4.09×10^{-29}
8	*bcn92*	FBgn0013432	comp8618_c0	571	comp4704_c0	833	gi|346473133|gb|AEO36411.1| hypothetical protein [*Amblyomma maculatum*]	1.14×10^{-20}
9	*Bet1*	FBgn0260857	comp8203_c0	883	comp6130_c0	842	gi|229294182|gb|EEN64839.1| hypothetical protein BRAFLDRAFT_125614 [*Branchiostoma floridae*]	1.73×10^{-20}
10	*Bet5*	FBgn0260860	comp6947_c0	655	comp5439_c0	644	gi|89268748|emb|CAJ82631.1| trafficking protein particle complex 1 [*Xenopus (Silurana) tropicalis*]	6.84×10^{-48}
11	*blos2*	FBgn0036118	comp9623_c0	771	comp4925_c0	783	gi|239788527|dbj|BAH70938.1| ACYPI008364 [*Acyrthosiphon pisum*]	3.52×10^{-24}
12	*Cbp20*	FBgn0022943	comp2782_c0	727	comp5110_c0	669	gi|344313239|gb|AEN04479.1| putative nuclear cap-binding protein subunit 2 [*Plutella xylostella*]	7.72×10^{-66}
14	*CG10333*	FBgn0033962	comp5691_c0	885	comp2982_c0	831	gi|346471907|gb|AEO35798.1| hypothetical protein [*Amblyomma maculatum*]	6.02×10^{-73}
13	*CG10153*	FBgn0032690	comp6670_c0	2548	comp10490_c0	2124	gi|346466927|gb|AEO33308.1| hypothetical protein [*Amblyomma maculatum*]	0.00

Continued ->

Table S13. (Continued):

#	Gene Symbol	FlyBase gene	*T. californicum* component	Length (bp)	*T. grallator* component	Length (bp)	Best Reciprocal BLASTX hit[1]	E-value of best hit
15	*CG10418*	FBgn0036277	comp8377_c0	544	comp7448_c0	514	gi|301792294|ref|XP_002931116.1| PREDICTED: u6 snRNA-associated Sm-like protein LSm2-like [*Ailuropoda melanoleuca*]	$1.63x10^{-45}$
16	*CG10470*	FBgn0032746	comp5006_c0	859	comp3948_c0	4828	gi|215490934|gb|EEC00575.1| membrane protein, putative [*Ixodes scapularis*]	$7.53x10^{-70}$
17	*CG10508*	FBgn0037060	comp91435_c0	319	comp40549_c0	1141	gi|215493453|gb|EEC03094.1| conserved hypothetical protein [*Ixodes scapularis*]	$2.12x10^{-46}$
18	*CG11137*	FBgn0037199	comp3844_c0	809	comp2580_c0	776	gi|67083883|gb|AAY66876.1| putative membrane protein [*Ixodes scapularis*]	$1.00x10^{-69}$
19	*CG11293*	FBgn0034889	comp59431_c0	601	comp8399_c0	649	gi|321478706|gb|EFX89663.1| hypothetical protein DAPPUDRAFT_40617 [*Daphnia pulex*]	$1.12x10^{-36}$
20	*CG11562*	FBgn0031247	comp20343_c0	577	comp7977_c0	547	gi|156546723|ref|XP_001604669.1| PREDICTED: hypothetical protein LOC100121087 [*Nasonia vitripennis*]	$1.41x10^{-06}$
21	*CG11985*	FBgn0040534	comp5526_c0	480	comp7213_c0	534	gi|307175620|gb|EFN65529.1| Probable splicing factor 3B subunit 5 [*Camponotus floridanus*]	$1.33x10^{-36}$
22	*CG12012*	FBgn0035444	comp1970_c0	717	comp1934_c0	721	gi|229291425|gb|EEN62097.1| hypothetical protein BRAFLDRAFT_87997 [*Branchiostoma floridae*]	$8.54x10^{-16}$
23	*CG12050*	FBgn0032915	comp10202_c0	3179	comp12803_c0	2780	gi|291242722|ref|XP_002741255.1| PREDICTED: WD repeat domain 75-like [*Saccoglossus kowalevskii*]	$4.82x10^{-113}$
24	*CG12077*	FBgn0035435	comp11091_c0	1307	comp8627_c0	1907	gi|318087060|gb|ADV40121.1| phosphatidylinositol N-acetylglucosaminyltransferase subunit C [*Latrodectus hesperus*]	$5.67x10^{-102}$
25	*CG12929*	FBgn0033429	comp24077_c0	1513	comp9626_c0	939	gi|346473153|gb|AEO36421.1| hypothetical protein [*Amblyomma maculatum*]	$1.62x10^{-34}$
26	*CG12975*	FBgn0037061	comp7060_c0	679	comp8601_c0	754	gi|215493454|gb|EEC03095.1| conserved hypothetical protein [*Ixodes scapularis*]	$3.70x10^{-44}$
27	*CG13018*	FBgn0040751	comp12520_c0	709	comp17584_c0	497	gi|346466309|gb|AEO32999.1| hypothetical protein [*Amblyomma maculatum*]	$5.42x10^{-23}$
28	*CG13994*	FBgn0031772	comp8155_c0	828	comp7573_c1	788	gi|357607661|gb|EHJ65626.1| dermal papilla derived protein 13 [*Danaus plexippus*]	$1.50x10^{-14}$

Continued ->

Table S13. (Continued):

#	Gene Symbol	FlyBase gene	*T. californicum* component	Length (bp)	*T. grallator* component	Length (bp)	Best Reciprocal BLASTX hit[1]	E-value of best hit
29	*CG14022*	FBgn0031700	comp5712_c0	474	comp20379_c0	484	gi|270012910|gb|EFA09358.1| hypothetical protein TcasGA2_TC001919 [*Tribolium castaneum*]	$9.49x10^{-27}$
30	*CG14229*	FBgn0031059	comp5854_c0	834	comp5271_c0	1359	gi|193906706|gb|EDW05573.1| GI11017 [*Drosophila mojavensis*]	$1.46x10^{-10}$
31	*CG14286*	FBgn0038673	comp10546_c0	765	comp1911_c0	625	gi|363739761|ref|XP_415049.3| PREDICTED: UPF0488 protein C8orf33 homolog [*Gallus gallus*]	$2.33x10^{-09}$
32	*CG14301*	FBgn0038632	comp48975_c0	1484	comp40235_c0	1225	gi|270001714|gb|EEZ98161.1| hypothetical protein TcasGA2_TC000587 [*Tribolium castaneum*]	$1.35x10^{-25}$
33	*CG14414*	FBgn0030571	comp8462_c0	824	comp16372_c0	977	gi|37590400|gb|AAH59651.1| RNA binding motif protein 18 [*Danio rerio*]	$2.58x10^{-31}$
34	*CG14512*	FBgn0039639	comp11105_c0	856	comp9957_c0	797	gi|148683445|gb|EDL15392.1| mCG145726 [*Mus musculus*]	$1.20x10^{-35}$
35	*CG14669*	FBgn0037326	comp142104_c0	236	comp179832_c0	221	gi|215494220|gb|EEC03861.1| RAS-related protein, putative [*Ixodes scapularis*]	$1.45\ x10^{-22}$
36	*CG14757*	FBgn0033274	comp5889_c0	943	comp11166_c0	686	gi|321478706|gb|EFX89663.1| hypothetical protein DAPPUDRAFT_40617 [*Daphnia pulex*]	$6.58x10^{-39}$
37	*CG14871*	FBgn0038343	comp59586_c0	273	comp23616_c0	323	gi|167879921|gb|EDS43304.1| conserved hypothetical protein [*Culex quinquefasciatus*]	$5.55x10^{-06}$
38	*CG15387*	FBgn0031403	comp8868_c0	603	comp5075_c0	695	gi|332025613|gb|EGI65775.1| Protein C10 [*Acromyrmex echinatior*]	$2.73x10^{-24}$
39	*CG15456*	FBgn0040650	comp5170_c0	647	comp7070_c0	1621	gi|152013691|gb|ABS19961.1| selenoprotein W2 [*Artemia franciscana*]	$3.29x10^{-18}$
40	*CG15525*	FBgn0039732	comp6121_c0	638	comp4499_c0	649	gi|156717982|ref|NP_001096533.1| coiled-coil domain containing 12 [*Xenopus (Silurana) tropicalis*]	$7.88x10^{-36}$
41	*CG15881*	FBgn0036909	comp5985_c0	676	comp5026_c0	661	gi|221271937|sp|Q6PBK1.2|CCD58_DANRE RecName: Full=Coiled-coil domain-containing protein 58 [*Danio rerio*]	$1.58x10^{-39}$
42	*CG16986*	FBgn0035356	comp7333_c0	1377	comp5634_c0	2094	gi|348513197|ref|XP_003444129.1| PREDICTED: acyl-coenzyme A thioesterase 13-like [*Oreochromis niloticus*]	$1.02x10^{-32}$
43	*CG17266*	FBgn0033089	comp10841_c0	751	comp10704_c0	781	gi|215499378|gb|EEC08872.1| peptidyl-prolyl cis-trans isomerase H (PPIH), putative [*Ixodes scapularis*]	$1.31x10^{-95}$

Continued ->

Table S13. (Continued):

#	Gene Symbol	FlyBase gene	*T. californicum* component	Length (bp)	*T. grallator* component	Length (bp)	Best Reciprocal BLASTX hit[1]	E-value of best hit
44	*CG17331*	FBgn0032596	comp2548_c0	1295	comp2662_c0	956	gil225705784lgblACO08738.1l Proteasome subunit beta type 2 [*Oncorhynchus mykiss*]	$2.71x10^{-70}$
45	*CG17734*	FBgn0037890	comp1909_c0	1080	comp3316_c0	1557	gil357616749lgblEHJ70377.1l hypothetical protein KGM_20861 [*Danaus plexippus*]	$3.59x10^{-16}$
46	*CG1882*	FBgn0033226	comp5734_c0	2058	comp2285_c0	1705	gil346468149lgblAEO33919.1l hypothetical protein [*Amblyomma maculatum*]	$2.59x10^{-100}$
47	*CG2021*	FBgn0035271	comp8092_c0	539	comp6882_c0	544	gil291238793lreflXP_002739308.1l PREDICTED: U6 snRNA-associated Sm-like protein LSm8-like [*Saccoglossus kowalevskii*]	$9.11x10^{-40}$
48	*CG2862*	FBgn0031459	comp5587_c0	860	comp3148_c0	1357	gil318087056lgblADV40119.1l histidine triad nucleotide-binding protein 1 [*Latrodectus hesperus*]	$7.34x10^{-60}$
49	*CG30010*	FBgn0050010	comp16315_c0	916	comp6518_c0	837	gil198438007lreflXP_002128913.1l PREDICTED: similar to AGAP011455-PA [*Ciona intestinalis*]	$2.49x10^{-58}$
50	*CG30051*	FBgn0050051	comp11880_c0	1426	comp16391_c0	1544	gil215492399lgblEEC02040.1l transmembrane protein, putative [*Ixodes scapularis*]	$7.25x10^{-30}$
51	*CG30382*	FBgn0050382	comp3121_c0	1122	comp2020_c0	1115	gil215504584lgblEEC14078.1l proteasome subunit alpha type, putative [*Ixodes scapularis*]	$2.99x10^{-106}$
52	*CG31126*	FBgn0051126	comp4925_c0	611	comp6780_c0	763	gil189441826lgblAAI67635.1l LOC100170577 protein [*Xenopus (Silurana) tropicalis*]	$7.45x10^{-24}$
53	*CG31229*	FBgn0051229	comp8528_c0	921	comp9477_c0	1393	gil346473227lgblAEO36458.1l hypothetical protein [*Amblyomma maculatum*]	$1.33x10^{-50}$
54	*CG31460*	FBgn0051460	comp1965_c0	682	comp3152_c0	651	gil126307787lreflXP_001373924.1l PREDICTED: keratinocyte-associated protein 2-like [*Monodelphis domestica*]	$1.94x10^{-35}$
55	*CG31812*	FBgn0051812	comp7010_c0	1188	comp4246_c0	1246	gil215506247lgblEEC15741.1l tRNA-splicing endonuclease subunit Sen2, putative [*Ixodes scapularis*]	$4.87x10^{-41}$
56	*CG31855*	FBgn0051855	comp6725_c0	1007	comp6129_c0	1299	gil346470911lgblAEO35300.1l hypothetical protein [*Amblyomma maculatum*]	$3.60x10^{-24}$
57	*CG31954*	FBgn0051954	comp96809_c0	496	comp238815_c0	110	gil345488667lreflXP_003425960.1l PREDICTED: trypsin-1-like [*Nasonia vitripennis*]	$3.89x10^{-36}$
58	*CG31957*	FBgn0051957	comp4940_c0	637	comp7326_c0	930	gil67083851lgblAAY66860.1l unknown [*Ixodes scapularis*]	$1.16x10^{-36}$

Continued ->

Table S13. (Continued):

#	Gene Symbol	FlyBase gene	*T. californicum* component	Length (bp)	*T. grallator* component	Length (bp)	Best Reciprocal BLASTX hit[1]	E-value of best hit
59	*CG32069*	FBgn0052069	comp6060_c0	488	comp8355_c0	546	gil346474138lgblAEO36913.1l hypothetical protein [*Amblyomma maculatum*]	$1.66x10^{-26}$
60	*CG32163*	FBgn0052163	comp10406_c0	675	comp9443_c0	632	gil215498406lgblEEC07900.1l protein GTLF3B, putative [*Ixodes scapularis*]	$1.85x10^{-14}$
61	*CG32202*	FBgn0052202	comp9340_c0	718	comp7834_c0	856	gil291237485lreflXP_002738666.1l PREDICTED: TCF3 (E2A) fusion partner-like [*Saccoglossus kowalevskii*]	$3.88x10^{-08}$
62	*CG32676*	FBgn0052676	comp17716_c0	1549	comp28545_c0	1903	gil350411717lreflXP_003489431.1l PREDICTED: hypothetical protein LOC100742717 [*Bombus impatiens*]	$1.16x10^{-35}$
63	*CG33054*	FBgn0053054	comp2941_c0	2823	comp9720_c0	5029	gil215500364lgblEEC09858.1l sn-1,2-diacylglycerol ethanolamine- and cholinephosphotranferase, putative [*Ixodes scapularis*]	$1.05x10^{-132}$
64	*CG33260*	FBgn0053260	comp9362_c0	1616	comp5793_c0	1389	gil229291379lgblEEN62052.1l hypothetical protein BRAFLDRAFT_239658 [*Branchiostoma floridae*]	$6.69x10^{-40}$
65	*CG3337*	FBgn0038871	comp7708_c0	866	comp5994_c0	849	gil291244037lreflXP_002741906.1l PREDICTED: hypothetical protein [*Saccoglossus kowalevskii*]	$1.34x10^{-54}$
66	*CG3436*	FBgn0031229	comp8780_c0	1271	comp9013_c0	1590	gil346468849lgblAEO34269.1l hypothetical protein [*Amblyomma maculatum*]	$1.47x10^{-150}$
67	*CG3501*	FBgn0034791	comp4399_c0	1705	comp9056_c0	3906	gil325303810ltpglDAA34579.1l TPA_exp: COX4 neighbor [*Amblyomma variegatum*]	$5.47x10^{-57}$
68	*CG3812*	FBgn0030421	comp17342_c0	1513	comp14425_c0	1165	gil229273367lgblEEN44219.1l hypothetical protein BRAFLDRAFT_57443 [*Branchiostoma floridae*]	$1.73x10^{-52}$
69	*CG40002*	FBgn0058002	comp2985_c0	460	comp4062_c0	474	gil270001025lgblEEZ97472.1l hypothetical protein TcasGA2_TC011303 [*Tribolium castaneum*]	$2.06x10^{-13}$
70	*CG40467*	FBgn0069977	comp41934_c0	766	comp424155_c0	131	gil212518472lgblEEB20225.1l sucrose transport protein, putative [*Pediculus humanus corporis*]	$8.79x10^{-57}$
71	*CG4729*	FBgn0036623	comp9359_c0	2945	comp59121_c0	637	gil215494818lgblEEC04459.1l 1-acyl-glycerol-3-phosphate acyltransferase, putative [*Ixodes scapularis*]	$1.73x10^{-92}$
72	*CG5316*	FBgn0038704	comp11988_c0	800	comp1820_c0	1226	gil229278633lgblEEN49420.1l hypothetical protein BRAFLDRAFT_206553 [*Branchiostoma floridae*]	$2.34x10^{-55}$

Continued ->

Table S13. (Continued):

#	Gene Symbol	FlyBase gene	*T. californicum* component	Length (bp)	*T. grallator* component	Length (bp)	Best Reciprocal BLASTX hit[1]	E-value of best hit
73	*CG5323*	FBgn0034362	comp7891_c0	766	comp1326_c0	1047	gi|215495983|gb|EEC05624.1| conserved hypothetical protein [*Ixodes scapularis*]	$7.53x10^{-48}$
74	*CG5569*	FBgn0034919	comp8182_c0	1034	comp7359_c0	1772	gi|312375320|gb|EFR22716.1| hypothetical protein AND_14308 [*Anopheles darlingi*]	$2.09x10^{-26}$
75	*CG5676*	FBgn0032200	comp7025_c0	881	comp4054_c0	712	gi|67083877|gb|AAY66873.1| FUN14 family protein [*Ixodes scapularis*]	$6.61x10^{-29}$
76	*CG5991*	FBgn0026576	comp20443_c0	605	comp266_c0	858	gi|215493690|gb|EEC03331.1| phosphatidylserine decarboxylase, putative [*Ixodes scapularis*]	$6.80x10^{-59}$
77	*CG6015*	FBgn0038927	comp7991_c0	2014	comp10110_c0	1849	gi|215500098|gb|EEC09592.1| mRNA splicing factor, putative [*Ixodes scapularis*]	0.0
78	*CG6196*	FBgn0038323	comp7339_c0	892	comp4421_c0	904	gi|108880527|gb|EAT44752.1| trafficking protein particle complex subunit 6b [*Aedes aegypti*]	$2.28x10^{-52}$
79	*CG6272*	FBgn0036126	comp3123_c0	1783	comp3833_c0	1803	gi|215500538|gb|EEC10032.1| CEBPA: CCAAT/enhancer-binding protein alpha, putative [*Ixodes scapularis*]	$4.27x10^{-24}$
80	*CG6610*	FBgn0035675	comp4847_c0	553	comp7614_c0	600	gi|212517833|gb|EEB19672.1| conserved hypothetical protein [*Pediculus humanus corporis*]	$1.68x10^{-39}$
81	*CG6770*	FBgn0032400	comp1073_c0	669	comp2286_c0	623	gi|260908473|gb|ACX53956.1| p8 nuclear protein [*Rhipicephalus sanguineus*]	$2.42x10^{-20}$
82	*CG6878*	FBgn0036488	comp4701_c0	1819	comp16154_c0	434	gi|167871430|gb|EDS34813.1| conserved hypothetical protein [*Culex quinquefasciatus*]	$2.00x10^{-29}$
83	*CG6959*	FBgn0037956	comp20344_c0	3005	comp161150_c0	167	gi|321469022|gb|EFX80004.1| hypothetical protein DAPPUDRAFT_103849 [*Daphnia pulex*]	$1.66x10^{-66}$
84	*CG7224*	FBgn0031971	comp7360_c0	717	comp6521_c0	689	gi|307180266|gb|EFN68299.1| UPF0369 protein C6orf57-like protein [*Camponotus floridanus*]	$1.72x10^{-16}$
85	*CG7637*	FBgn0033548	comp4577_c0	566	comp7559_c0	456	gi|321470985|gb|EFX81959.1| hypothetical protein DAPPUDRAFT_49574 [*Daphnia pulex*]	$2.45x10^{-20}$
86	*CG7718*	FBgn0038649	comp7134_c0	1859	comp8464_c0	1833	gi|348508490|ref|XP_003441787.1| PREDICTED: CDP-diacylglycerol--glycerol-3-phosphate 3-phosphatidyltransferase, mitochondrial-like [*Oreochromis niloticus*]	$1.50x10^{-133}$
87	*CG7757*	FBgn0036915	comp2349_c0	2630	comp5704_c0	2112	gi|215504843|gb|EEC14337.1| u4/u6 small nuclear ribonucleoprotein, putative [*Ixodes scapularis*]	0.00

Continued ->

Table S13. (Continued):

#	Gene Symbol	FlyBase gene	*T. californicum* component	Length (bp)	*T. grallator* component	Length (bp)	Best Reciprocal BLASTX hit[1]	E-value of best hit
88	*CG7789*	FBgn0039698	comp5667_c0	1164	comp6077_c0	1118	gi|55846015|gb|AAV67032.1| nucleotidase 4F8 [*Ixodes scapularis*]	$4.18x10^{-106}$
89	*CG7920*	FBgn0039737	comp922_c0	2798	comp2570_c0	2147	gi|215509553|gb|EEC19006.1| acetyl-CoA hydrolase, putative [*Ixodes scapularis*]	0.00
90	*CG7949*	FBgn0036107	comp2659_c0	982	comp3867_c0	1300	gi|348501652|ref|XP_003438383.1| PREDICTED: mitotic spindle-associated MMXD complex subunit MIP18-like [*Oreochromis niloticus*]	$9.17x10^{-36}$
91	*CG8009*	FBgn0036090	comp9344_c0	1232	comp4911_c0	756	gi|346470057|gb|AEO34873.1| hypothetical protein [*Amblyomma maculatum*]	$1.83x10^{-51}$
92	*CG9099*	FBgn0030802	comp8453_c0	819	comp6195_c0	754	gi|215504698|gb|EEC14192.1| Density-regulated protein, putative [*Ixodes scapularis*]	$1.10x10^{-56}$
93	*CG9410*	FBgn0033086	comp3696_c0	955	comp2948_c0	973	gi|327284649|ref|XP_003227049.1| PREDICTED: coenzyme Q-binding protein COQ10 homolog B, mitochondrial-like [*Anolis carolinensis*]	$1.31x10^{-56}$
94	*CG9586*	FBgn0032101	comp9010_c0	1200	comp5354_c0	1040	gi|346468303|gb|AEO33996.1| hypothetical protein [*Amblyomma maculatum*]	$1.02x10^{-12}$
95	*CG9921*	FBgn0030743	comp1737_c0	698	comp4825_c0	539	gi|193900811|gb|EDV99677.1| GH12463 [*Drosophila grimshawi*]	$1.79x10^{-19}$
96	*Chrac-16*	FBgn0043001	comp8670_c0	946	comp7397_c0	930	gi|290462639|gb|ADD24367.1| Chromatin accessibility complex protein 1 [*Lepeophtheirus salmonis*]	$9.61x10^{-17}$
97	*cpx*	FBgn0041605	comp14185_c0	1896	comp7874_c0	4004	gi|333466649|gb|EGK96327.1| AGAP003509-PG [*Anopheles gambiae str.* PEST]	$2.41x10^{-17}$
98	*Csas*	FBgn0052220	comp37891_c0	981	comp37511_c0	1220	gi|346469695|gb|AEO34692.1| hypothetical protein [*Amblyomma maculatum*]	$1.97x10^{-63}$
99	*Cyt-b5*	FBgn0033189	comp2403_c0	1014	comp4446_c0	3030	gi|346470009|gb|AEO34849.1| hypothetical protein [*Amblyomma maculatum*]	$1.18x10^{-42}$
100	*Cyt-c-p*	FBgn0000409	comp887_c0	652	comp167_c0	645	gi|318087086|gb|ADV40134.1| putative cytochrome c [*Latrodectus hesperus*]	$5.43x10^{-53}$
101	*Dim1*	FBgn0031601	comp7497_c0	919	comp9227_c0	1956	gi|296473912|gb|DAA16027.1| thioredoxin-like 4A [*Bos taurus*]	$1.77x10^{-71}$
102	*dj-1beta*	FBgn0039802	comp4774_c0	1513	comp3845_c0	1461	gi|229296224|gb|EEN66865.1| hypothetical protein BRAFLDRAFT_94906 [*Branchiostoma floridae*]	$5.74x10^{-48}$

Continued ->

Table S13. (Continued):

#	Gene Symbol	FlyBase gene	*T. californicum* component	Length (bp)	*T. grallator* component	Length (bp)	Best Reciprocal BLASTX hit[1]	E-value of best hit
103	*dpa*	FBgn0015929	comp11247_c0	2109	comp16093_c0	2932	gi|215500547|gb|EEC10041.1| DNA replication licensing factor, MCM4 component, putative [*Ixodes scapularis*]	0.00
104	*Dpy-30L2*	FBgn0035491	comp3062_c0	755	comp2500_c0	832	gi|332021244|gb|EGI61629.1| Protein dpy-30-like protein [*Acromyrmex echinatior*]	$7.28x10^{-16}$
105	*Dsk*	FBgn0000500	comp21382_c0	635	comp11014_c0	610	gi|321469918|gb|EFX80896.1| putative sulfakinin-like peptide [*Daphnia pulex*]	$6.59x10^{-07}$
106	*Fer3HCH*	FBgn0030449	comp484_c0	881	comp612_c0	850	gi|33772683|gb|AAQ54711.1| ferritin [*Dermacentor albipictus*]	$3.52x10^{-74}$
107	*Fis1*	FBgn0039969	comp2977_c0	761	comp4400_c0	810	gi|260908558|gb|ACX53998.1| tetratricopeptide repeat protein [*Rhipicephalus sanguineus*]	$3.42x10^{-37}$
108	*FK506-bp2*	FBgn0013954	comp33544_c0	511	comp12670_c0	1744	gi|321457218|gb|EFX68309.1| hypothetical protein DAPPUDRAFT_231749 [*Daphnia pulex*]	$7.40x10^{-50}$
109	*few*	FBgn0261722	comp8658_c0	1240	comp18768_c0	3309	gi|350397911|ref|XP_003485028.1| PREDICTED: calcium channel flower-like isoform 2 [*Bombus impatiens*]	$2.24x10^{-28}$
110	*HSPC300*	FBgn0061198	comp10036_c0	571	comp5809_c0	659	gi|318087562|gb|ADV40371.1| syntaxin interacting protein 1 [*Latrodectus africana*]	$2.67x10^{-31}$
111	*Ilk*	FBgn0028427	comp3124_c0	3856	comp5845_c0	2092	gi|215494148|gb|EEC03789.1| tyrosine kinase, putative [*Ixodes scapularis*]	0.00
112	*Ilp7*	FBgn0044046	comp11668_c0	648	comp9265_c0	665	gi|37220726|gb|AAQ89696.1| insulin-like peptide 5 precursor [*Anopheles gambiae*]	$1.82x10^{-16}$
113	*jagn*	FBgn0037374	comp9133_c0	3627	comp3556_c0	776	gi|215493384|gb|EEC03025.1| protein jagunal, putative [*Ixodes scapularis*]	$9.40x10^{-30}$
114	*ksh*	FBgn0040890	comp4654_c0	978	comp5766_c0	461	gi|114153262|gb|ABI52797.1| conserved hypothetical protein DUF1242 [*Argas monolakensis*]	$9.23x10^{-22}$
115	*l(1)G0136*	FBgn0026666	comp1090_c0	981	comp1977_c0	1603	gi|193906956|gb|EDW05823.1| GI16259 [*Drosophila mojavensis*]	$4.61x10^{-43}$
116	*l(2)03709*	FBgn0010551	comp2250_c0	1156	comp2028_c0	1147	gi|215504760|gb|EEC14254.1| prohibitin, putative [*Ixodes scapularis*]	$2.69x10^{-105}$
117	*l(2)06225*	FBgn0010612	comp1739_c0	1211	comp1488_c0	580	gi|321471678|gb|EFX82650.1| hypothetical protein DAPPUDRAFT_230756 [*Daphnia pulex*]	$3.27x10^{-24}$

Continued ->

Table S13. (Continued):

#	Gene Symbol	FlyBase gene	*T. californicum* component	Length (bp)	*T. grallator* component	Length (bp)	Best Reciprocal BLASTX hit[1]	E-value of best hit
118	*l(2)35Di*	FBgn0001989	comp3380_c0	783	comp4780_c0	761	gi|346472013|gb|AEO35851.1| hypothetical protein [*Amblyomma maculatum*]	$3.31x10^{-32}$
119	*l(2)efl*	FBgn0011296	comp1429_c0	850	comp1724_c0	838	gi|318087266|gb|ADV40225.1| putative alpha-B-crystallin [*Latrodectus africana*]	$7.23x10^{-45}$
120	*l(3)01239*	FBgn0010741	comp2972_c0	918	comp2063_c0	1237	gi|346472623|gb|AEO36156.1| hypothetical protein [*Amblyomma maculatum*]	$1.04x10^{-33}$
121	*Las*	FBgn0029158	comp4664_c0	1341	comp7053_c0	1342	gi|344279337|ref|XP_003411445.1| PREDICTED: lipoyl synthase, mitochondrial-like [*Loxodonta africana*]	$4.61x10^{-139}$
122	*levy*	FBgn0034877	comp1694_c0	634	comp2067_c0	479	gi|346466541|gb|AEO33115.1| hypothetical protein [*Amblyomma maculatum*]	$3.52x10^{-29}$
123	*LSm1*	FBgn0261067	comp4507_c0	1233	comp7100_c0	1261	gi|346471247|gb|AEO35468.1| hypothetical protein [*Amblyomma maculatum*]	$6.93x10^{-38}$
124	*LSm3*	FBgn0051184	comp13453_c0	658	comp6278_c0	2364	gi|215494871|gb|EEC04512.1| snrnp sm protein, putative [*Ixodes scapularis*]	$3.82x10^{-30}$
125	*LSm7*	FBgn0261068	comp4762_c0	1128	comp10330_c0	545	gi|215496302|gb|EEC05942.1| small nuclear ribonucleoprotein (snRNP) splicing factor [*Ixodes scapularis*]	$2.85x10^{-44}$
126	*mad2*	FBgn0035640	comp9485_c0	818	comp6285_c0	833	gi|229281497|gb|EEN52255.1| hypothetical protein BRAFLDRAFT_260671 [*Branchiostoma floridae*]	$1.17x10^{-65}$
127	*MED10*	FBgn0036581	comp2729_c0	634	comp2643_c0	608	gi|346465831|gb|AEO32760.1| hypothetical protein [*Amblyomma maculatum*]	$3.05x10^{-58}$
128	*mge*	FBgn0035473	comp1814_c0	615	comp3660_c0	814	gi|325303298|tpg|DAA34785.1| TPA_inf: outer mitochondrial membrane complex subunit TOM22 translocase [*Amblyomma variegatum*]	$9.90x10^{-17}$
129	*Mp20*	FBgn0002789	comp2055_c0	722	comp1699_c0	685	gi|346466263|gb|AEO32976.1| hypothetical protein [*Amblyomma maculatum*]	$4.98x10^{-79}$
130	*mRpL11*	FBgn0038234	comp1698_c0	2181	comp5197_c0	904	gi|318087566|gb|ADV40373.1| transmembrane protein 183 [*Latrodectus hesperus*]	$8.70x10^{-91}$
131	*mRpL20*	FBgn0036335	comp6235_c0	725	comp3186_c0	691	gi|332024521|gb|EGI64719.1| 39S ribosomal protein L20, mitochondrial [*Acromyrmex echinatior*]	$2.02x10^{-28}$
132	*mRpL48*	FBgn0031357	comp4495_c0	1093	comp5625_c0	2262	gi|346472977|gb|AEO36333.1| hypothetical protein [*Amblyomma maculatum*]	$1.75x10^{-34}$

Continued ->

Table S13. (Continued):

#	Gene Symbol	FlyBase gene	*T. californicum* component	Length (bp)	*T. grallator* component	Length (bp)	Best Reciprocal BLASTX hit[1]	E-value of best hit
133	*mRpL51*	FBgn0032053	comp4460_c0	704	comp3837_c0	655	gi|110758045|ref|XP_001120055.1| PREDICTED: 39S ribosomal protein L51, mitochondrial [*Apis mellifera*]	4.96×10^{-29}
134	*mRpL52*	FBgn0033208	comp4769_c0	525	comp7777_c0	1129	gi|66911776|gb|AAH97777.1| MGC115481 protein [*Xenopus laevis*]	2.72×10^{-14}
135	*mRpS14*	FBgn0044030	comp8422_c0	728	comp6907_c0	863	gi|215503776|gb|EEC13270.1| ribosomal protein S14, putative [*Ixodes scapularis*]	2.54×10^{-29}
136	*mRpS28*	FBgn0034361	comp6857_c0	1007	comp6274_c0	996	gi|307185697|gb|EFN71613.1| 28S ribosomal protein S28, mitochondrial [*Camponotus floridanus*]	3.21×10^{-32}
137	*Nedd8*	FBgn0032725	comp2991_c0	610	comp4620_c0	905	gi|198138643|gb|EAL33960.2| GA10488 [*Drosophila pseudoobscura pseudoobscura*]	3.75×10^{-26}
138	*noi*	FBgn0014366	comp6722_c0	1747	comp7522_c0	2141	gi|346467927|gb|AEO33808.1| hypothetical protein [*Amblyomma maculatum*]	0.00
139	*Nxt1*	FBgn0028411	comp6695_c0	606	comp5165_c0	893	gi|346466501|gb|AEO33095.1| hypothetical protein [*Amblyomma maculatum*]	2.71×10^{-29}
140	*Oscp*	FBgn0016691	comp2341_c0	885	comp1982_c0	2412	gi|346470663|gb|AEO35176.1| hypothetical protein [*Amblyomma maculatum*]	5.42×10^{-57}
141	*ox*	FBgn0011227	comp2598_c0	752	comp4103_c0	648	gi|47223633|emb|CAF99242.1| unnamed protein product [*Tetraodon nigroviridis*]	1.09×10^{-13}
142	*pallidin*	FBgn0036192	comp72144_c0	442	comp44636_c0	982	gi|215502389|gb|EEC11883.1| Pallidin, putative [*Ixodes scapularis*]	6.56×10^{-23}
143	*PDCD-5*	FBgn0036580	comp4296_c0	853	comp3041_c0	853	gi|193918577|gb|EDW17444.1| GI12672 [*Drosophila mojavensis*]	3.41×10^{-18}
144	*Pdsw*	FBgn0021967	comp3913_c0	762	comp3425_c0	2772	gi|167880174|gb|EDS43557.1| NADH dehydrogenase [Culex quinquefasciatus]	1.22×10^{-42}
145	*pen-2*	FBgn0053198	comp5268_c0	508	comp7238_c0	656	gi|346470161|gb|AEO34925.1| hypothetical protein [*Amblyomma maculatum*]	8.48×10^{-38}
146	*PHGPx*	FBgn0035438	comp67505_c0	992	comp2386_c0	989	gi|77166828|gb|ABA62390.1| phospholipid-hydroperoxide glutathione peroxidase [*Rhipicephalus microplus*]	7.85×10^{-68}
147	*Rpb4*	FBgn0053520	comp9621_c0	852	comp3356_c0	899	gi|346469951|gb|AEO34820.1| hypothetical protein [*Amblyomma maculatum*]	7.25×10^{-61}

Continued ->

Table S13. (Continued):

#	Gene Symbol	FlyBase gene	*T. californicum* component	Length (bp)	*T. grallator* component	Length (bp)	Best Reciprocal BLASTX hit[1]	E-value of best hit
148	*RpII15*	FBgn0004855	comp4750_c0	591	comp12830_c0	570	gi|215499697|gb|EEC09191.1| RNA polymerase II subunit, putative [*Ixodes scapularis*]	$1.86x10^{-63}$
149	*RpL11*	FBgn0013325	comp281_c0	905	comp1262_c0	729	gi|318087194|gb|ADV40189.1| putative ribosomal protein L11 [*Latrodectus hesperus*]	5.48x10-102
150	*RpL13A*	FBgn0037351	comp843_c0	772	comp854_c0	777	gi|161661015|gb|ABX75375.1| 60S ribosomal protein L13A [*Lycosa singoriensis*]	$9.52x10^{-99}$
151	*RpL15*	FBgn0028697	comp917_c0	886	comp936_c0	792	gi|346469771|gb|AEO34730.1| hypothetical protein [*Amblyomma maculatum*]	$1.14x10^{-94}$
152	*RpL17*	FBgn0029897	comp951_c0	969	comp730_c0	1314	gi|318087202|gb|ADV40193.1| 60S ribosomal protein l17 [*Latrodectus hesperus*]	$1.73x10^{-88}$
153	*RpL19*	FBgn0002607	comp313_c0	760	comp880_c0	751	gi|318087190|gb|ADV40187.1| putative ribosomal protein L19e [*Latrodectus hesperus*]	$7.02x10^{-80}$
154	*RpL24*	FBgn0032518	comp1128_c0	647	comp1204_c0	645	gi|161669228|gb|ABX75466.1| ribosomal protein l24 [*Lycosa singoriensis*]	$1.05x10^{-48}$
155	*RpL3*	FBgn0020910	comp123999_c0	569	comp890_c0	1324	gi|66566113|ref|XP_624821.1| PREDICTED: 60S ribosomal protein L3 [*Apis mellifera*]	$1.81x10^{-95}$
156	*RpL32*	FBgn0002626	comp1081_c0	579	comp942_c0	563	gi|318087004|gb|ADV40094.1| ribosomal protein L32 isoform B [*Latrodectus hesperus*]	$2.72x10^{-65}$
157	*RpL35*	FBgn0029785	comp1008_c0	551	comp1132_c0	506	gi|161661027|gb|ABX75380.1| 60S ribosomal protein L35 [*Lycosa singoriensis*]	$1.40x10^{-41}$
158	*RpL35A*	FBgn0037328	comp517_c0	590	comp973_c0	567	gi|67084027|gb|AAY66948.1| ribosomal protein L35a [*Ixodes scapularis*]	$4.82x10^{-48}$
159	*RpL36A*	FBgn0031980	comp1261_c0	517	comp1031_c0	471	gi|114153164|gb|ABI52748.1| ribosomal protein L44 [*Argas monolakensis*]	$3.20x10^{-54}$
160	*RpL38*	FBgn0040007	comp31445_c0	412	comp1110_c0	441	gi|307763913|gb|EFO23147.1| 60S ribosomal protein L38 [*Loa loa*]	$2.43x10^{-30}$
161	*RpL9*	FBgn0015756	comp4752_c0	888	comp295_c0	696	gi|149287182|gb|ABR23490.1| 60S ribosomal protein L9 [*Ornithodoros parkeri*]	$2.35x10^{-76}$
162	*RpS10b*	FBgn0261593	comp85_c0	642	comp941_c0	638	gi|318087010|gb|ADV40097.1| ribosomal protein S10 [*Latrodectus hesperus*]	$1.91x10^{-79}$
163	*RpS11*	FBgn0033699	comp218_c0	674	comp566_c0	666	gi|332026048|gb|EGI66199.1| 40S ribosomal protein S11 [*Acromyrmex echinatior*]	$2.22x10^{-57}$

Continued ->

Table S13. (Continued):

#	Gene Symbol	FlyBase gene	*T. californicum* component	Length (bp)	*T. grallator* component	Length (bp)	Best Reciprocal BLASTX hit[1]	E-value of best hit
164	*RpS13*	FBgn0010265	comp953_c0	618	comp892_c0	827	gi|229366468|gb|ACQ58214.1| 40S ribosomal protein S13 [*Anoplopoma fimbria*]	4.09×10^{-73}
165	*RpS15*	FBgn0034138	comp1121_c0	624	comp682_c0	918	gi|161661035|gb|ABX75384.1| 40s ribosomal protein S15 [*Lycosa singoriensis*]	1.07×10^{-68}
166	*RpS15Aa*	FBgn0010198	comp567_c0	405	comp1144_c0	661	gi|318086978|gb|ADV40081.1| ribosomal protein S15 [*Latrodectus hesperus*]	2.36×10^{-57}
167	*RpS16*	FBgn0034743	comp1041_c0	611	comp1079_c0	659	gi|318086972|gb|ADV40078.1| ribosomal protein S16 [*Latrodectus hesperus*]	1.65×10^{-74}
168	*RpS18*	FBgn0010411	comp1144_c0	604	comp915_c0	619	gi|318086970|gb|ADV40077.1| ribosomal protein S18 [*Latrodectus hesperus*]	7.58×10^{-67}
169	*RpS26*	FBgn0261597	comp927_c0	535	comp1154_c0	563	gi|346465965|gb|AEO32827.1| hypothetical protein [*Amblyomma maculatum*]	1.57×10^{-52}
170	*RpS27*	FBgn0039300	comp871_c0	504	comp1128_c0	492	gi|161669268|gb|ABX75486.1| 40S ribosomal protein S27 [*Lycosa singoriensis*]	1.46×10^{-43}
171	*RpS27A*	FBgn0003942	comp893_c0	638	comp230_c0	621	gi|318086966|gb|ADV40075.1| 60S ribosomal protein L40A [*Latrodectus hesperus*]	2.73×10^{-62}
172	*RpS28b*	FBgn0030136	comp1571_c0	1220	comp1550_c0	418	gi|149286900|gb|ABR23349.1| 40S ribosomal protein S28 [*Ornithodoros parkeri*]	1.73×10^{-20}
173	*RpS29*	FBgn0261599	comp172_c0	472	comp1281_c0	445	gi|149287094|gb|ABR23446.1| 40S ribosomal protein S29 [*Ornithodoros parkeri*]	8.18×10^{-26}
174	*Sep1*	FBgn0011710	comp18493_c0	1817	comp26466_c0	1949	gi|346471777|gb|AEO35733.1| hypothetical protein [*Amblyomma maculatum*]	1.74×10^{-166}
175	*SmD2*	FBgn0261789	comp5053_c0	592	comp2048_c0	604	gi|291224549|ref|XP_002732266.1| PREDICTED: Small Nuclear Ribonucleoprotein family member (snr-4)-like [*Saccoglossus kowalevskii*]	4.41×10^{-50}
176	*SmG*	FBgn0261791	comp2695_c0	769	comp3878_c0	668	gi|62083383|gb|AAX62416.1| small nuclear ribonucleoprotein G [*Lysiphlebus testaceipes*]	1.68×10^{-28}
177	*sno*	FBgn0005410	comp5111_c0	4708	comp7268_c0	5358	gi|340717232|ref|XP_003397090.1| PREDICTED: protein strawberry notch-like [*Bombus terrestris*]	0.00
178	*snRNP-U1-C*	FBgn0261792	comp3284_c0	847	comp5144_c0	1036	gi|212509775|gb|EEB13104.1| U1 small nuclear ribonucleoprotein C, putative [*Pediculus humanus corporis*]	2.24×10^{-36}

Continued ->

Table S13. (Continued):

#	Gene Symbol	FlyBase gene	*T. californicum* component	Length (bp)	*T. grallator* component	Length (bp)	Best Reciprocal BLASTX hit[1]	E-value of best hit
179	*Spase25*	FBgn0030306	comp3339_c0	991	comp1159_c0	740	gi|346469711|gb|AEO34700.1| hypothetical protein [*Amblyomma maculatum*]	$4.20x10^{-77}$
180	*Spf45*	FBgn0086683	comp9724_c0	1611	comp5445_c0	1690	gi|215497936|gb|EEC07430.1| DNA-damage-repair/toleration protein DRT111, putative [*Ixodes scapularis*]	$4.14x10^{-58}$
181	*Srp14*	FBgn0038808	comp3680_c0	506	comp7077_c0	1605	gi|260908358|gb|ACX53900.1| signal recognition particle 14 kD protein [*Rhipicephalus sanguineus*]	$6.53x10^{-31}$
182	*Stlk*	FBgn0046692	comp7318_c0	1771	comp10058_c0	3323	gi|291237811|ref|XP_002738826.1| PREDICTED: STE20-related kinase adaptor alpha-like [*Saccoglossus kowalevskii*]	$4.23x10^{-85}$
183	*Sucb*	FBgn0029118	comp3621_c0	1670	comp10902_c0	2817	gi|215490921|gb|EEC00562.1| GTP-specific succinyl-CoA synthetase, beta subunit, putative [*Ixodes scapularis*]	$5.90x10^{-166}$
184	*Taf12*	FBgn0011290	comp6146_c0	929	comp2915_c0	803	gi|351695935|gb|EHA98853.1| Transcription initiation factor TFIID subunit 12 [*Heterocephalus glaber*]	$2.37x10^{-39}$
185	*Tim8*	FBgn0027359	comp2809_c0	496	comp9275_c0	544	gi|149287190|gb|ABR23494.1| mitochondrial import inner membrane translocase subunit Tim8-like protein [*Ornithodoros parkeri*]	$3.07x10^{-22}$
186	*Tim9a*	FBgn0030480	comp8371_c0	686	comp8859_c0	831	gi|346472045|gb|AEO35867.1| hypothetical protein [*Amblyomma maculatum*]	$4.00x10^{-28}$
187	*Tom7*	FBgn0033357	comp2313_c0	502	comp6997_c0	491	gi|307168239|gb|EFN61465.1| Mitochondrial import receptor subunit TOM7-like protein [*Camponotus floridanus*]	$2.48x10^{-14}$
188	*Tsf2*	FBgn0036299	comp91325_c0	830	comp59043_c0	1337	gi|215504246|gb|EEC13740.1| transferrin, putative [*Ixodes scapularis*]	$3.78x10^{-125}$
189	*UbcD4*	FBgn0015321	comp4743_c0	1084	comp3545_c0	1150	gi|346465869|gb|AEO32779.1| hypothetical protein [*Amblyomma maculatum*]	$6.91x10^{-92}$
190	*UbcD6*	FBgn0004436	comp6398_c0	1511	comp3616_c0	2829	gi|346469893|gb|AEO34791.1| hypothetical protein [*Amblyomma maculatum*]	$6.26x10^{-87}$
191	*ubl*	FBgn0022224	comp4686_c0	572	comp5382_c0	604	gi|161671302|gb|ABX75503.1| ubiquitin-like protein 5 [*Lycosa singoriensis*]	$5.27x10^{-34}$

Continued ->

Table S13. (Continued):

#	Gene Symbol	FlyBase gene	*T. californicum* component	Length (bp)	*T. grallator* component	Length (bp)	Best Reciprocal BLASTX hit[1]	E-value of best hit
192	*Vha14-1*	FBgn0262512	comp3184_c0	1145	comp5199_c0	1063	gi|307210307|gb|EFN86937.1| Vacuolar proton pump subunit F [*Harpegnathos saltator*]	2.25×10^{-47}
193	*Vkor*	FBgn0053544	comp9210_c0	1067	comp1971_c0	2285	gi|67083823|gb|AAY66846.1| vitamin K epoxide reductase complex subunit 1 precursor [*Ixodes scapularis*]	1.10×10^{-43}
194	*Vps36*	FBgn0086785	comp11164_c0	1817	comp13231_c0	2400	gi|346471943|gb|AEO35816.1| hypothetical protein [*Amblyomma maculatum*]	1.77×10^{-108}
195	*ymp*	FBgn0261287	comp2066_c0	829	comp2717_c0	947	gi|215500430|gb|EEC09924.1| eukaryotic translation initiation factor 4E binding protein, putative [*Ixodes scapularis*]	2.63×10^{-28}
196	*Zasp66*	FBgn0035917	comp841_c0	1666	comp3068_c0	1564	gi|215492667|gb|EEC02308.1| PDZ domain protein [*Ixodes scapularis*]	3.69×10^{-43}

[1]Only the BLASTX hit with the lowest E-value (*T. californicum* or *T. grallator* is shown).

29) Literature Cited:

1. Huson DH, Auch AF, Qi J, Schuster SC: **MEGAN analysis of metagenomic data.** *Genome Research* 2007, **17**:377-386.
2. Segata N, Waldron L, Ballarini A, Narasimhan V, Jousson O, Huttenhower C: **Metagenomic microbial community profiling using unique clade-specific marker genes**. *Nat Methods* 2012, **9**(8):811-814.
3. Tatusov RL, Fedorova ND, Jackson JD, Jacobs AR, Kiryutin B, Koonin EV, Krylov DM, Mazumder R, Mekhedov SL, Nikolskaya AN *et al*: **The COG database: an updated version includes eukaryotes.** *BMC Bioinformatics* 2003, **4**:41.
4. Parra G, Bradnam K, Korf I: **CEGMA: a pipeline to accurately annotate core genes in eukaryotic genomes**. *Bioinformatics* 2007, **23**(9):1061-1067.
5. Parra G, Bradnam K, Ning Z, Keane T, Korf I: **Assessing the gene space in draft genomes**. *Nucleic Acids Res* 2009, **37**(1):289-297.
